# Rapidly acquired HIV-1 neutralization breadth in a macaquized V2 apex mouse model after a single bolus immunization

**DOI:** 10.1126/sciimmunol.adz5064

**Published:** 2026-02-13

**Authors:** Amrit Raj Ghosh, Rumi Habib, Nitesh Mishra, Ryan S. Roark, Madhav Akauliya, Ali A. Albowaidey, Joel D. Allen, Khaled Amereh, Gabriel Avillion, Maria Bottermann, Bo Liang, Namit Chaudhary, Sean Callaghan, Jonathan Dye, Xuduo Li, Jordan R. Ellis-Pugh, Rohan Roy Chowdhury, Nicole E. James, Xiaotie Liu, Laura Maiorino, Paula M. Villavicencio, Rebecca Nedellec, Prabhgun Oberoi, Kirsten J. Sowers, Younghoon Park, Thavaleak Prum, Linette Rodriguez, Maria Ssozi, Jonathan L. Torres, Agnes A. Walsh, John E. Warner, Stephanie R. Weldon, Liling Xu, Kevin Wiehe, Max Crispin, Andrew B. Ward, Usha Nair, Beatrice H. Hahn, Dennis R. Burton, Lawrence Shapiro, Peter D. Kwong, Darrell J. Irvine, Raiees Andrabi, George M. Shaw, Facundo D. Batista

**Affiliations:** 1 Batista Lab, Ragon Institute of Mass General Brigham, MIT, and Harvard, Cambridge, MA, 02139, USA; 2 Departments of Medicine and Microbiology, Perelman School of Medicine, University of Pennsylvania, Philadelphia, PA 19104, USA; 3 Vaccine and Immunotherapy Center, The Wistar Institute, Philadelphia, PA 19104, USA; 4 Department of Immunology and Microbiology, The Scripps Research Institute, La Jolla, CA 92037, USA; 5 Consortium for HIV/AIDS Vaccine Development (CHAVD), The Scripps Research Institute, La Jolla, CA 92037, USA; 6 Aaron Diamond AIDS Research Center, Vagelos College of Physicians and Surgeons, Columbia University, New York, NY 10032, USA; 7 Department of Biochemistry and Molecular Biophysics, Columbia University, New York, NY 10027, USA; 8 Koch Institute for Integrative Cancer Research, Massachusetts Institute of Technology, Cambridge, MA 02139 USA.; 9 Howard Hughes Medical Institute, 29 Chevy Chase, MD 20815 USA; 10 School of Biological Sciences, University of Southampton, Southampton SO17 1BJ, UK.; 11 Department of Integrative Structural and Computational Biology, The Scripps Research Institute, La Jolla, CA 28 92037, USA; 12 Duke Human Vaccine Institute, Duke University School of Medicine, Durham, NC 27710, USA; 13 IAVI Neutralizing Antibody Center, The Scripps Research Institute, La Jolla, CA 92037, USA; 14 Ragon Institute of Mass General Brigham, MIT, and Harvard, Cambridge, MA, 02139, USA; 15 Department of Biological Engineering, Massachusetts Institute of Technology, Cambridge, MA 02139, United States.; 16 Department of Biology, Massachusetts Institute of Technology, Cambridge, MA 02139, United States.; 17 Department of Immunology, Harvard Medical School, Boston, MA 02115, USA

## Abstract

Current immunization strategies to elicit broadly neutralizing antibodies (bnAbs) against HIV-1 generally propose complex, multi-boost regimens. In rhesus macaques, simian-human immunodeficiency virus (SHIV) infection rapidly drives the development of some bnAb classes sharing structural similarities with those in humans. Here, we generated a knockin (KI) mouse model with B cells bearing the unmutated common ancestor (UCA) of a V2 apex-targeted bnAb lineage, V033-a. A single immunization with a germline-targeting native-like trimer, Q23-APEX-GT1, recapitulated the ontogeny of the mature rhesus bnAb in KI mice, including rare, disfavored somatic mutations. Resulting antibodies exhibited potent neutralization against a broad panel of heterologous HIV-1 viruses. Boosting with Env escape mutant trimers further improved breadth and potency, and cryo-EM analysis revealed the structural basis for heterologous neutralization breadth. Non-human primate and mouse models combined with structure can serve as a platform for identifying and validating immunogens that streamline HIV-vaccination regimens.

## Summary

A single germline-targeting trimer immunization induced neutralization breadth in a rhesus germline V2-apex knockin mouse model.

## Introduction

The diversity of circulating HIV-1 strains has proven a major challenge for vaccine development (1). Nonetheless, the discovery of broadly neutralizing antibodies (bnAbs) in a subset of people living with HIV has inspired the design of potentially effective HIV-1 vaccines (2-4). bnAbs arise following an initial priming of rare naive B cells with appropriate VDJ sequences followed by a complex coevolutionary process between the escaping HIV-1 envelope protein (Env) and antibody lineages produced by affinity-matured B cells (5-8). A common strategy to reproduce this trajectory by vaccination is similarly complex: immunization with priming immunogens to selectively activate naïve B cells with specific features critical to bnAb epitope recognition, followed by a further sequence of Env immunogens to drive breadth and potency (9-14). Priming immunogens designed for this approach are being evaluated in clinical trials, with some showing promise (NCT06033209, NCT04224701, NCT05001373, NCT05471076) (15, 16), such as eOD-GT8 60mer inducing precursors to VRC01-class bnAbs in 97% of recipients (17). However, each additional vaccination step increases both logistical challenges and the likelihood of incomplete immunization.

The expectation of extensive boosting arises from the complex developmental pathways of bnAbs to many canonical epitopes, which often involves substantial somatic hypermutation (SHM). VRC01-class bnAbs, which are specific to the CD4-binding site (CD4bs), are characterized by extreme SHM, with 20–35% nucleotide variation from germline in the heavy chain (HC) and 15–20% in the light chain (LC) producing amino acid identities of less than 50% (18). By contrast, V2 apex bnAbs, which are commonly observed in HIV-infected human cohorts (19-21) and in simian-human immunodeficiency virus (SHIV)-infected macaques (22), may require relatively low rates of SHM, develop rapidly after priming of their germline unmutated common ancestors (UCA), and can be shepherded by only a few Env escape mutations (22-25). Therefore, V2-apex bnAb UCAs may have simpler maturation pathways than bnAbs to other specificities.

Env Q23.17 (26) has been identified in several screens of large virus panels as sensitive to neutralization by germline-reverted V2-apex bnAbs, suggestive of an intrinsic germline-targeting (GT) property (27-29). This property is confirmed in an accompanying manuscript where we demonstrate that SHIV-Q23.17 rapidly elicits V2-apex bnAbs in as many as 50% of infected macaques (22). Whereas longitudinal lineage tracing of V2-apex directed antibodies from HIV-infected patients has been performed (24, 25), lineage tracing in rhesus macaques allows fine tracking of bnAb development after infection via frequent B cell sampling. One V2-apex lineage arising after SHIV-Q23.17 infection, V033-a, has been chosen for further study because of its well-described ontogeny, neutralization breadth, potency, and the short timeframe in which V033-a arose (22, 30).

Immunoglobulin knockin (KI) mouse models have been commonly deployed to examine candidate GT vaccine designs (10, 13, 31, 32). However, even in favorable KI mouse models, such vaccine strategies required complex boosting (9, 11, 14, 33-38). We asked whether we could recapitulate the short ontogeny of the V033-a lineage in a KI mouse model to expedite the elicitation of V2-apex bnAbs by vaccination. To do so, we knocked in an early precursor of the V033-a lineage (V033a-UCA I1) that differs from the phylogenetically inferred UCA by just one amino acid in the nontemplated junction between D and J (22)—generating a macaquized immunoglobulin mouse model.

Using these models, we demonstrated that native-like stabilized HIV Env Q23.17 (Q23-APEX-GT1), can bind KI V033a-UCA I1 B cells. A single bolus priming immunization of Q23-APEX-GT1 was sufficient to select for critical mutations and endow isolated antibodies with notable neutralization breadth. Furthermore, a single boost resulted in additional neutralization breadth and potency, with an escape variant boost outperforming a homologous boost. Thus, we demonstrate that some near-native Envs are capable of priming and guiding the maturation of V2 apex bnAbs, that at least a subset of V2 apex bnAb UCAs can rapidly acquire substantial breadth and potency, and that the design of boosting immunogens based on Env escape may be deployed to mature primed bnAb precursors, recapitulating B cell evolution observed in macaques after SHIV infection.

## Results

### Structure-guided design of native-like Q23-APEX-GT1 trimer for priming V2-apex bnAb B cell precursors

We previously identified a subset of primary HIV Envs that contain fewer potential N-linked glycosylation (PNG) sites in their V1V2 segments than most other wildtype Envs, enabling V2 apex bnAb inferred germline B cell receptor (BCR) binding to native-like trimers (28, 29, 39), suggesting that these native-like trimers could engage bnAb precursors at the V2 apex site without extensive protein engineering. One such Env, Q23.17, consistently induced V2 apex bnAbs in a rhesus macaque SHIV infection model (22). Accordingly, in this study we sought to design a Q23.17 Env-based trimer immunogen that could be delivered across various platforms, including mRNA lipid nanoparticles (LNPs).

**Figure 1.**
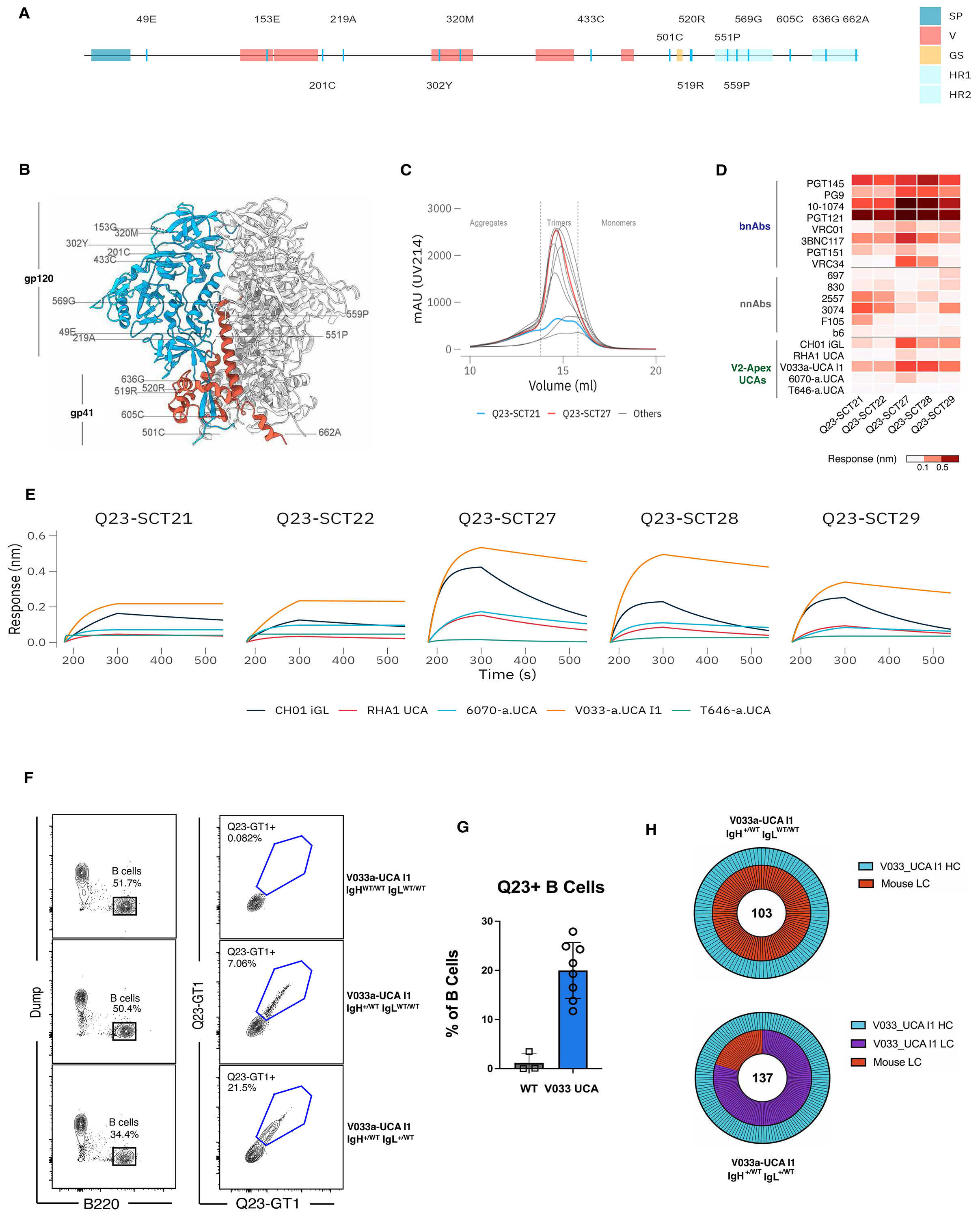
Generation and characterization of the Q23-SCT immunogen and V033a-UCA I1 mouse model. (**A**) Structure-guided mutations utilized in construct Q23-SCT27 are labelled and shown on a schematic map of HIV-1 Env. Signal peptide = blue; variable loops = red; flexible glycine-serine linker (G4S)2 = yellow; HR1 and HR2 region within gp41 = sky blue. (**B**) Amino acid substitutions in Q23-SCT27 are labelled and shown on the crystal structure of Q23 DS-SOSIP.664 trimer (PDB: 7LLK) with one protomer colored according to sub-regions (gp120 in blue and gp41 in red). (**C**) Representative size exclusion chromatography (SEC) profile of Galanthus-nivalis (GNL) purified Q23-SCTs. The fractions corresponding to aggregates, trimer and dimer/monomers are annotated. Fractions used for antigenic profiling are shown inside dotted lines. The lead candidate, Q23-SCT27, is shown in red, while the base construct is shown in blue. All remaining constructs are shown in gray. (**D**) Antigenic profile of five single chain trimers (SCTs) against a small panel of bnAbs, unmutated common ancestors (UCA), inferred germline (iGL) and non-neutralizing antibodies was performed with bio-layer interferometry (BLI). Maximum response values across five independent measurements were used for plotting heat map. (**E**) Kinetic curves from BLI for the five SCTs from panel D against the five RM V2 apex UCAs. V033 I1 showed highest stable binding against Q23-SCT27 to Q23-SCT29. (**F**) Representative FACS plots of peripheral B220^+^ B cell binding of Q23-SCT27 in C57BL6/J, V033a-UCA I1 IgH^+/WT^ IgL^WT/WT^ and V033a-UCA I1 IgH^+/WT^ IgL^+/WT^ mice. Major ticks mark log 10 scale. (**G**) Quantification of peripheral B cell binding of Q23-SCT27 in C57BL6/J and V033a-UCA I1 IgH^+/WT^ IgL^+/WT^. (**H**) Paired sequences of single-cell sorted Q23-APEX-GT1-binding naïve B cells (upper) V033a-UCA I1 IgH^+/WT^ IgL^WT/WT^ and (lower) V033a-UCA I1 IgH^+/WT^ IgL^+/WT^ mice (n=2 each). Outer circle shows HC identity; inner indicates light chain (LC). V033a-UCA I1 HC = teal; V033A-UCA I1 LC = purple; native murine LC = red.

To generate prefusion-stabilized native-like Q23 Env trimers, we combined previously described NFL (native flexibly linked) and RnS (repair-and-stabilization) designs (40, 41) with additional antibody-guided structure-based mutations. We optimized the trimer subdomains to enhance hydrophilic interactions at the apex, restrict CD4-induced conformational changes, and minimize off-target V3 epitope exposure. Stabilization targeted both gp120 (including V1V2 and V3 regions) and gp41 subunits (Fig. 1A-B and fig. S1-S2). We systematically combined stabilized subdomain variants, covalently linked them with a glycine-serine linker, and assessed trimer formation using size-exclusion chromatography (SEC). The final construct, a covalently linked gp120-gp41 trimer, is referred to as a single-chain trimer (SCT). The initial design, Q23-SCT21, incorporated SOSIP mutations (501C-605C, I559P) and a disulfide-(DS) bond (201C-433C) to reduce CD4-induced epitope exposure (42, 43). This core mutation set was retained across all ten variants (Q23-SCT21 – Q23-SCT30), with subsequent iterations introducing gp41 and gp120 stabilizing mutations (fig. S1-S2).

For gp41 stabilization, the Q23-SCT22 construct incorporated glycine substitutions at positions 569 and 636 to reduce helical flexibility (44), Q551P to rigidify gp41, and 519R-520R to decrease the fusion peptide's hydrophobicity. The Q23-SCT23 construct included seven RnS-derived mutations (535N, 556P, 588E, 589V, 651F, 655I, 658V) (40), while Q23-SCT24 combined all twelve gp41-stabilizing mutations from both Q23-SCT22 and Q23-SCT23. Lastly, Q23-SCT25 maintained these mutations but replaced the 501C-605C disulfide bond with a 501C-663C linkage (45). For gp120 stabilization, six additional mutations were introduced: 49E, 153E, 219A, 302Y, 320M, and 334S. The mutations 49E, 302Y, and 320M were derived from NFL-TD8 (46), 153E was selected based on structural analysis (PDB 7LLK) to stabilize the V2 apex, and 219A and 334S were adapted from RnS using the ADROITrimer (47). These gp120 mutations were systematically incorporated into the following constructs: Q23-SCT26 = Q23-SCT21 + gp120 mutations, Q23-SCT27 = Q23-SCT22 + gp120 mutations, Q23-SCT28 = Q23-SCT23 + gp120 mutations, Q23-SCT29 = Q23-SCT24 + gp120 mutations, and Q23-SCT30 = Q23-SCT25 + gp120 mutations. This iterative design enhanced trimer stability and antigenic properties (fig. S1-S2).

SEC profiles of *Galanthus nivalis* lectin (GNL)-purified Q23-SCT soluble proteins revealed distinct peaks for aggregates, trimers, and dimer/monomers in most designs (Fig. 1C). Four Q23-SCT variants—Q23-SCT22, Q23-SCT27, Q23-SCT28, and Q23-SCT29—were selected based on higher trimer yield, sharper trimer peaks, and reduced aggregate or monomer/dimer peaks, indicating improved trimer assembly compared to the base construct (Q23-SCT21), which contained minimal mutations (Fig. 1C and fig. S1A-B). The antigenic profile of the SCTs was evaluated using bio-layer interferometry (BLI) against a panel of HIV Env bnAbs, non-neutralizing antibodies (nnAbs), and UCAs and iGLs of V2 apex bnAbs (Fig. 1D). Q23-SCT27 demonstrated enhanced BLI binding to several bnAbs and substantially reduced binding to V2i (697-30D and 830a) and CD4bs non-nAbs (b6 and F105), suggesting improved antigenic properties (Fig. 1D). Notably, Q23-SCT27 exhibited enhanced binding to several V2-apex bnAb UCAs and iGLs, including both human (CH01 iGL and PCT64 LMCA) and rhesus (RHA1 UCA and V033a-UCA I1). A comprehensive antigenic analysis of Q23-SCT27 with a larger panel of 70 monoclonal antibodies (mAbs), divided into bnAbs and nnAbs, further confirmed its broad reactivity across different specificities (fig. S1C). This binding was consistent with the mammalian cell surface-expressed trimer version of Q23-SCT27 (with wild-type Q23.17 transmembrane domain) and neutralization of the wild-type Q23.17 virus (fig. S3). Of note, the cell surface-expressed trimer showed enhanced binding, likely due to avidity.

Negative stain electron microscopy (ns-EM) of Q23-SCT27 trimers revealed well-formed trimeric structures with distinct 2D-averaged classes, confirming structural integrity (fig. S1D). Proteomics-based site-specific glycan analysis (SSGA) of Q23-SCT27 showed a diverse glycan profile (fig. S1E). Notably, no glycan signal could be resolved at PNGS 141 and 401, suggesting inherent flexibility not amenable to protease digestion. The well-formed trimeric structure and native-like glycan profile of Q23-SCT27, notably the conservation of oligomannose-type glycans at N160, validated its suitability as a vaccine candidate.

Based on these properties, we selected Q23-SCT27, henceforth called Q23-APEX-GT1 due to its enhanced binding to V2-apex bnAb UCAs and iGLs, as our lead candidate for preclinical testing.

### Generation of KI mice expressing a precursor to a rhesus macaque bnAb

To model Q23-APEX-GT1 engagement in a stringent pre-clinical mouse model, we used our established CRISPR/Cas9-mediated KI method (48, 49) to introduce the heavy and light chains of the V033-a lineage UCA designated V033a-UCA I1. The naïve germline precursor, or UCA, was derived from analyses of early sequential IgM^+^ naïve B cell and IgG^+^ memory B cell immunoglobulin sequences from the peripheral blood of macaque V033. Phylogenetic analysis of these sequences coalesced to two HCs that differed by a single amino acid in the nontemplated D-J junction and by a single amino acid in VL (22). For construction of the KI mouse lines, we selected one of these paired sequences, V033a-UCA I1, as it exhibited a higher binding affinity and greater neutralization potency against the Q23.17 Env. We could also determine its high resolution cryoEM structure in complex with the Q23.17 Env trimer (22). The successful KI was confirmed through genotyping and is referred to as the V033a-UCA I1 mouse. Peripheral B cells from V033a-UCA I1 mice bound Q23-APEX-GT1 trimer conjugated to streptavidin probes in the presence or absence of V033A-UCA I1 KI LC (Fig. 1F); for V033a-UCA I1 IgH^+/WT^ IgL^+/WT^, ~20% of peripheral B cells bound the probe (Fig. 1G). BCR sequencing of Q23-APEX-GT1-binding B cells using 10x Genomics revealed that V033a-UCA I1 HC paired with multiple murine LC in V033a-UCA I1 IgH^+/WT^ IgL^WT/WT^ mice, whereas in V033a-UCA I1 IgH^+/WT^ IgL^+/WT^ mice, the majority of KI HCs paired with KI LCs (Fig. 1H). These results confirmed the successful knockin of macaque V033a-UCA I1 into a murine model.

### Q23-APEX-GT1 protein trimer and Q23-APEX-GT1 saRNA efficiently activate V033a-UCA I1 B cells

To determine whether the Q23-APEX-GT1 trimer could activate V033a-UCA I1 B cells in vivo, we transferred V033a-UCA I1 IgH^+/WT^ IgL^+/WT^ CD45.2^+/+^ B cells intravenously (IV) into recipient CD45.1^+/+^ C57BL/6J to establish a frequency of 8 CD45.2s in 10^6^ total B cells (48, 50). One day later, adoptively transferred animals were immunized subcutaneously (SC) in the base of the tail with 10 μg of Q23-APEX-GT1 prepared in 5 μg of saponin/MPLA nanoparticles (SMNP) adjuvant (51) (fig. S4A). The immune responses in the draining inguinal lymph nodes were analyzed at weeks 2 and 4 post-immunization (wpi). Immunized animals at 2 wpi were able to form germinal centers (GCs) (Dump^−^ B220+ CD38^lo^ CD95^+^ among live cells) and these GCs were sustained 4 wpi (fig. S4B-D). Notably, a substantial fraction of these GCs was composed of adoptively transferred CD45.2 V033a-UCA I1 B cells, with ~40% binding Q23-APEX-GT1 (fig. S4C,E), demonstrating successful activation and GC recruitment of KI B cells by Q23-APEX-GT1 protein trimer priming.

**Figure 2.**
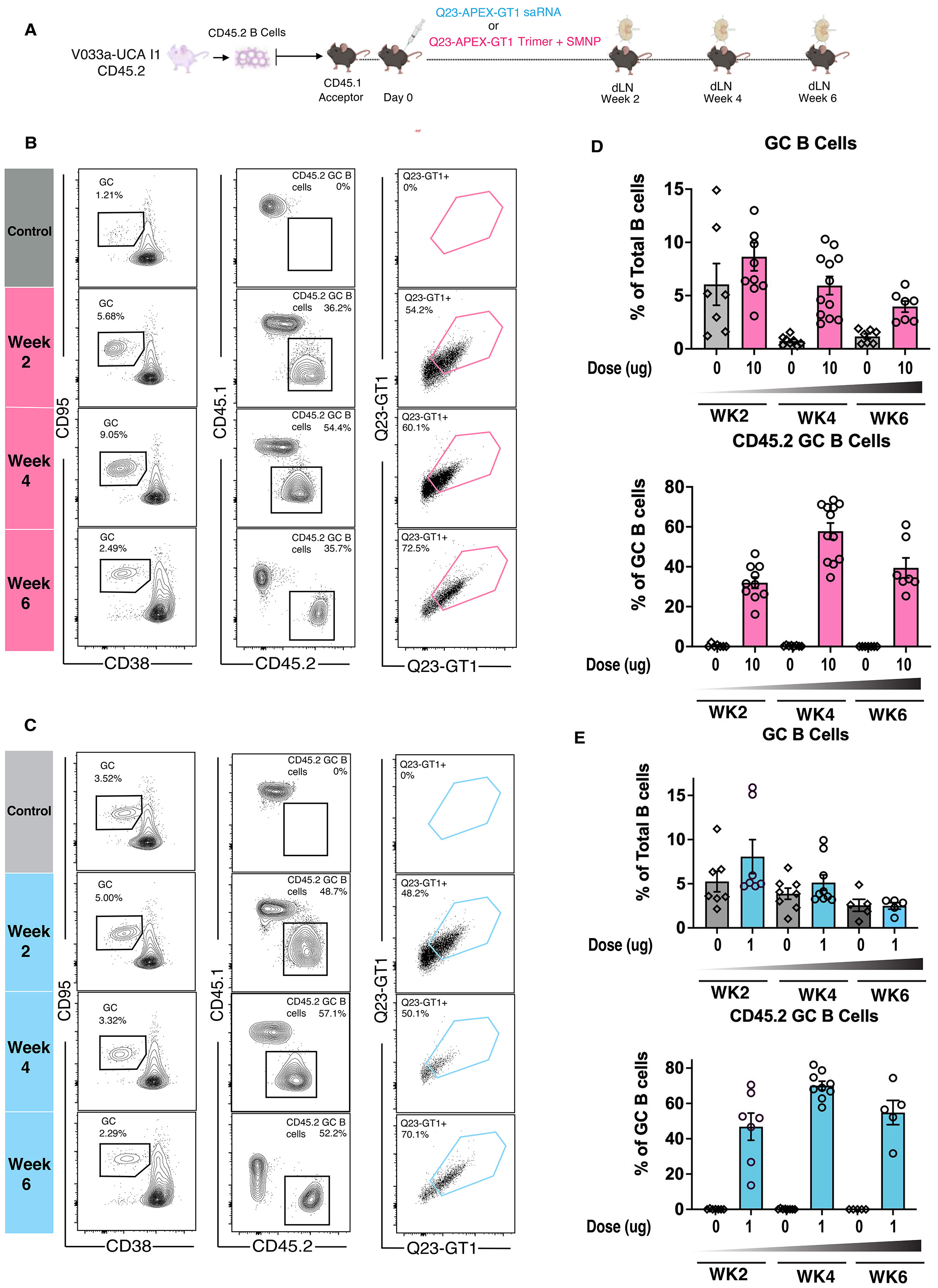
Q23-APEX-GT1 can prime V033a-UCA I1 B cells at low frequencies. (**A**) Schematic of mouse adoptive transfer and immunization experiments. WT CD45.1^+/+^ mice received V033a-UCA I1 CD45.2^+/+^ B cells through intravenous transfer one day prior to immunization with 1 μg per hindlimb (total 2 μg) of saRNA-LNP delivered intramuscularly (IM) or 10 μg of Q23-APEX-GT1 trimer adjuvanted with saponin/MPLA nanoparticles (SMNP) subcutaneously (SC). SMNP adjuvant without trimer was used as the control for protein immunizations and LNPs containing an unrelated saRNA for the Q23-APEX-GT1 saRNA LNP immunizations. Post-immunization, draining lymph nodes (dLNs) were isolated from immunized mice at indicated timepoints and were analyzed. n=7–10 mice per group; 3 independent experiments performed. (**B, C**) Representative FACS plots showing GCs, CD45.2 B cells in GCs, and their binding to Q23-APEX-GT1 during weeks 2, 4 and 6 post-immunization with Q23-APEX-GT1 trimer with (B) SMNP (B) or (C) LNPs containing Q23-APEX-GT1 saRNA. Major ticks mark log 10 scale. (**D**) Quantification of GC B cells (upper) and CD45.2 V033a-UCA I1 B cells in GCs (lower) in post-immunization by Q23-APEX-GT1 adjuvanted with SMNP (denoted as 10) or SMNP alone (0). Each group sums three independent immunizations of n=7–10/group, as described in A. WK=Week (**E**) Quantification of GC B cells (upper) and CD45.2 V033a-UCA I1 B cells in GCs (lower) in responses post Q23-APEX-GT1 saRNA LNP (denoted 1) or empty LNP immunization (0). Each group sums three independent immunizations of n=7–10/group, as described in A.

The low precursor frequency of V2-apex bnAb UCAs is a principal barrier to elicitation (22, 52-54). To recapitulate this in a model of immunization, we transferred V033a-UCA I1 IgH^+/WT^ IgL^+/WT^ CD45.2^+/+^ B cells IV into recipient CD45.1^+/+^ C57BL/6J mice (48, 50). One day later, animals were immunized with 10 μg of Q23-APEX-GT1 prepared in 5 μg of SMNP subcutaneously (Fig. 2A). Analysis of draining lymph nodes in immunized animals showed strong early GC responses (Dump^−^ B220+ CD38^lo^ CD95^+^ among live cells) with Q23-APEX-GT1 trimer, which diminished over time Fig. 2B,D). V033a-UCA I1 CD45.2 B cells resided in GCs up to 6 wpi with ~40% 2 wpi, ~60% 4 wpi, and ~40% 6 wpi (Fig. 2B,D). Immunization with Q23-APEX-GT1 trimer, therefore, elicited long-lasting precursor participation in GCs despite stringent initial frequencies.

For comparison with protein-based immunization, we developed a self-amplifying RNA (saRNA) (55), encoding a membrane-anchored form of Q23-APEX-GT1 utilizing the transmembrane domain of wild-type Q23.17 virus. To determine whether membrane-anchored Q23-APEX-GT1 delivered by saRNA could activate V033 precursors, we performed adoptive transfer as above and immunized recipients with Q23-APEX-GT1 saRNA containing LNPs intramuscularly (IM) (Fig. 2A). Activation and GC participation was similar to that obtained by trimer immunization out to week 4 (Fig. 2C,E). Thus, Q23-APEX-GT1, delivered as either a trimer protein with SMNP or saRNA LNP encoding membrane-bound trimer, effectively activates V033a-UCA I1 B cells and supports Q23-APEX-GT1-specific durable GC responses.

### A single Q23-APEX-GT1 immunization leads to substantial on-track V033-a mutations

**Figure 3.**
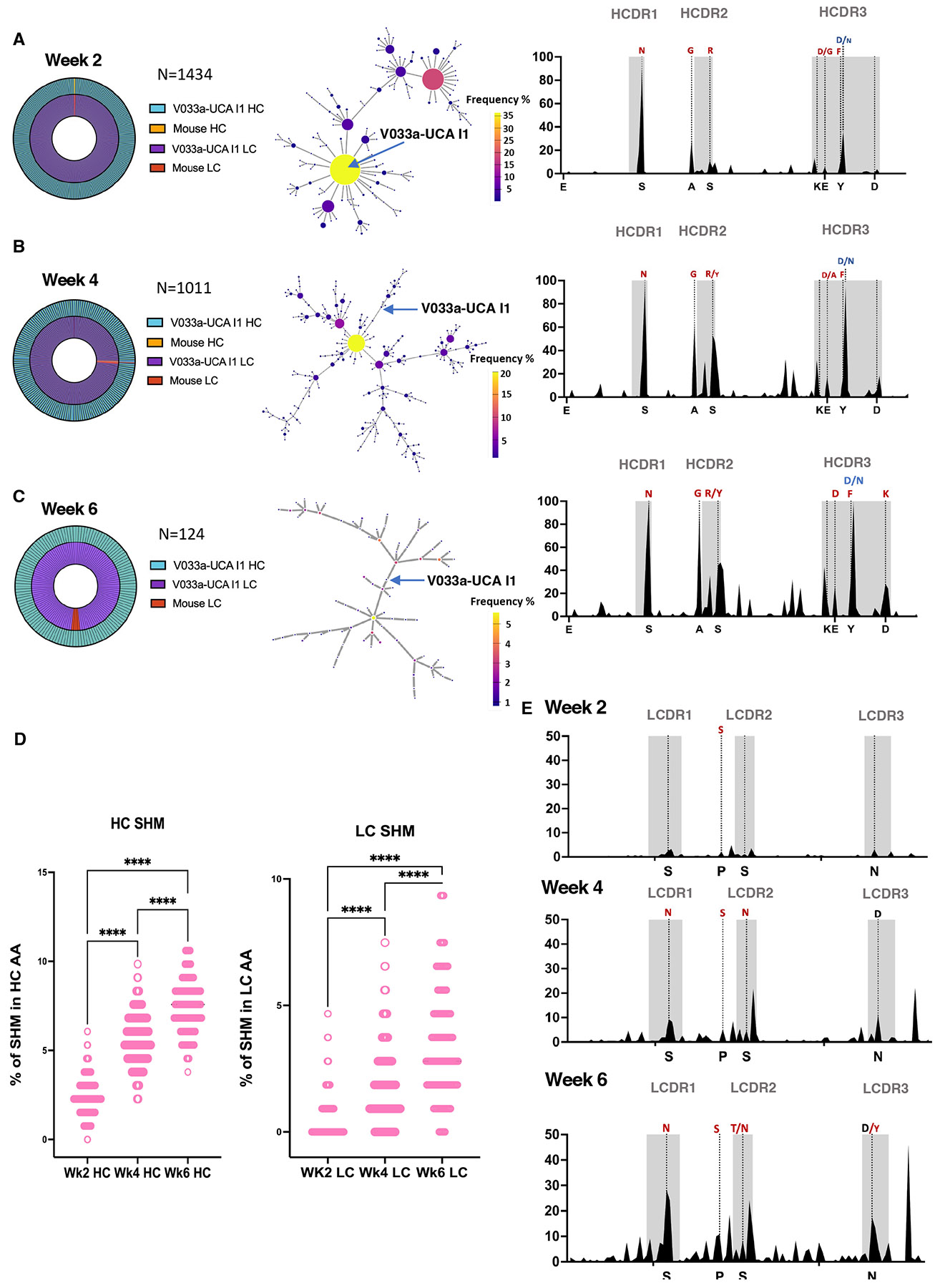
Priming leads to recapitulation of V033 ontogeny and rapid gain of neutralization breadth. (**A, B, C**) (Left, outer) Heavy chain (HC) and (left, inner) light chain (LC) usage by single cells from Q23-APEX-GT1-specific CD45.2^+^ sorted (A) 2, (B) 4, and (C) 6 weeks post-immunization with Q23-APEX-GT1 trimer adjuvanted with SMNP. (Center) Divergence of KI V033a-UCA I1 HCDR3 post-immunization represented through a cluster similarity network. (Right) HC mutation frequencies; selected mutations in V033a-UCA I1 HC present in mature V033 lineage bnAbs are represented in red and mutations found at intermediate stages of V033 lineage development (22) are marked in blue. (**D**) Total amino acid (AA) mutations in V033a-UCA I1 IGHV and IGKV at weeks 2, 4, 6 post immunization. Non-parametric one-way ANOVA (Kruska-Wallis) was performed to test statistical difference between groups. (**E**) LC mutation frequencies after 2, 4 and 6 weeks after immunization with Q23-APEX-GT1 trimer. Select mutations in present in mature V033 lineage bnAbs are represented in red; mutations found at intermediate stage of V033 lineage development is marked in blue.

We next investigated the degree to which a single immunization recapitulated the V033-a lineage ontogeny observed in macaques (fig. S6A). As we observed many antigen-positive CD45.2^+^ B cells participating in GCs after immunization, we isolated Q23-APEX-GT1^+^ CD45.2 B cells after protein trimer immunization (Fig. 2B, fig. S5) to examine clonal expansion and SHM. Analysis of V033a-UCA I1 KI BCRs in paired sequencing data revealed HC diversification (Fig. 3A). Several amino acid replacements either present in the mature rhesus macaque V033 bnAb or observed over the course of V033-a lineage maturation (22) appeared as early as 2 wpi (Fig. 3A). These “on-track” mutations accumulated over time (Fig. 3B-C). Furthermore, the HCDR3, which is important for V2-apex recognition, displayed rapid clonal divergence over time (Fig. 3A-C, **middle panel**). After immunization, the KI HC of the adoptively transferred B cells preferentially associated with the KI LC (Fig. 3A-C). Both HC and LC accumulated mutations rapidly after a single bolus priming immunization (Fig. 3D). As with HCs, KI LCs displayed on-track V033 mutations (Fig. 3E). Similar to protein trimer immunization, BCRs sequenced after saRNA immunization also demonstrated a rapid gain of SHM in V033a-UCA I1 HCs (Fig. S7A).

This rapid clonal divergence and accumulation of on-track mutations led us to express a subset of clones from 4 wpi and 6 wpi as mAbs for further characterization. All of the 4 wpi prime-derived mAbs had high affinity for Q23-APEX-GT1 (Fig. S8A). We therefore tested the efficacy of these antibodies against a limited panel of HIV-1 strains in a pseudovirus neutralization assay. All selected mAbs could neutralize the autologous Q23.17 virus with IC_50_ values ranging from 0.029–5.2 μg/ml. Some mAbs could neutralize heterologous viruses that are sensitive to V2-apex bnAb neutralization (Fig. S8B), albeit with a lesser potency than the mature V033-a.01 mAb. This subset of viruses was the first one to become sensitive to neutralization from V033 plasma in the NHP (22). Three of the selected mAbs from 4 wpi neutralized up to 43% of strains in a larger panel of heterologous tier 2 viruses (Fig. S8C)—tier 2 is both the most common circulating phenotype and considered challenging to neutralize (56). A single Q23-APEX-GT1 immunization thus drove substantial neutralization breadth.

### Neutralization breadth and potency attained late in single immunization-derived antibodies

**Figure 4.**
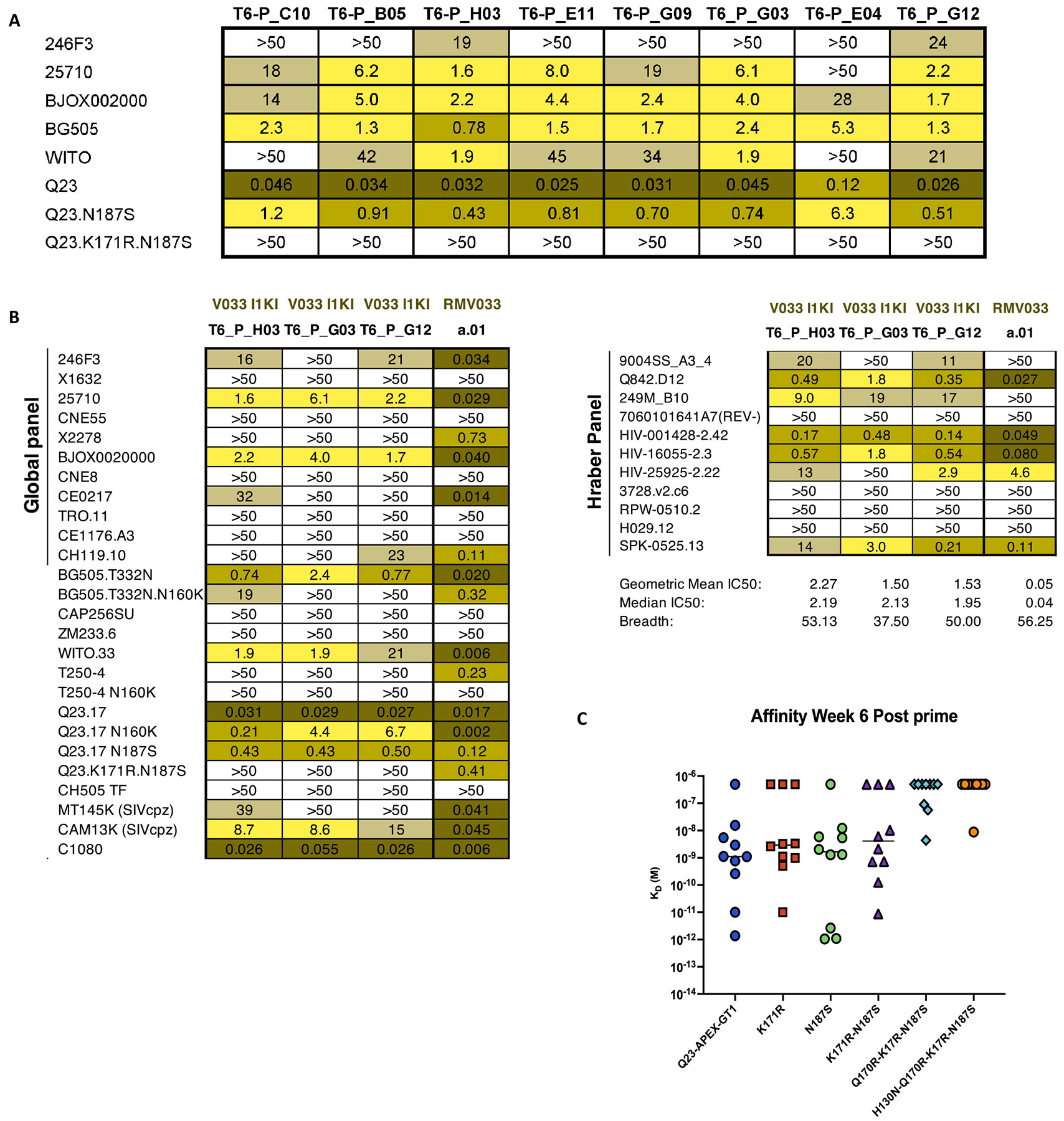
Neutralization breadth and potency of antibodies derived post-prime. (**A**) IC50 values of antibodies from week 6 post-priming by the protein trimer against autologous (Q23), escape variants (Q23.N187S, Q23.K171R.N187S) and limited panel of 5 heterologous tier-2 HIV-1 strains. Nomenclature: T = time in weeks; P = Prime; final alphanumeric triplet = mAb identity. (**B**) IC50 values of three post-prime antibodies from (A) against a 37-member panel of HIV-1 strains, including 11 from the Tier-2 Global panel and 11 from the Hraber panel (82); macaque mature V033 (RM V033-a.01) shown for comparison. (**C**) Affinities (K_D_ (M)) of antibodies (10 μg/ml) from Panel A (derived week 6 post-protein-prime) against autologous Q23-APEX-GT1 trimer and escape-variant Envs (500 nM).

Env-targeting bnAbs may be enriched for mutations that are less favored due to codon divergence and the ability to mutate when the residue is in AID “cold spots” (57). We observed that “on-track” mutations accumulated over time in V033a-UCA I1 KI BCRs, particularly in the CDRs, thus we sought to determine whether an increase in less favored mutations over the course of a longer GC residency could enhance potency and breadth. Mutation probability analysis using ARMADiLLO (57)of HC isolated from rhesus macaques post-SHIV infection predicted that rare mutations appear in the mature V033 lineage with greater frequency over time (fig. S6B). We used ARMADiLLO to select antibodies isolated from adoptively transferred mice 6 wpi which were enriched for less favored mutations (fig. S7B, C). The mAbs derived from 6 wpi were able to neutralize the autologous Q23.17 virus, as well as the first Env escape variant detected in the macaque, N187S, with higher potency (Fig. 4A) and demonstrated heterologous neutralization in a limited panel (Fig. 4A). In a larger panel of heterologous tier 2 virus strains, one mAb, T6-P_H03, showed neutralization breadth approaching that of mature V033 (Fig. 4B). Additionally, all 6 wpi-derived antibodies bound with high affinity to the escape variants of Q23 and other V2-Apex bnAb sensitive Envs (Fig. 4C). Furthermore, after priming with either protein trimer or saRNA, Q23-APEX-GT1 N187S binding was observed inside the GCs as early as 4 wpi and continued to mature through 6 wpi (fig. S9A-C). However, we observed only weak serum neutralization at later time points in the subset screened and tested after priming (4 wpi and 6 wpi), and only in animals that received Q23-APEX-GT1 trimer adjuvanted with SMNP (Fig. S10 A-B). Serum neutralization, therefore, was not extensive after a single bolus prime, but mAb affinity, neutralization, and B cell binding were nonetheless consistent with the early development of a response to the escape variant (N187S).

**Figure 5.**
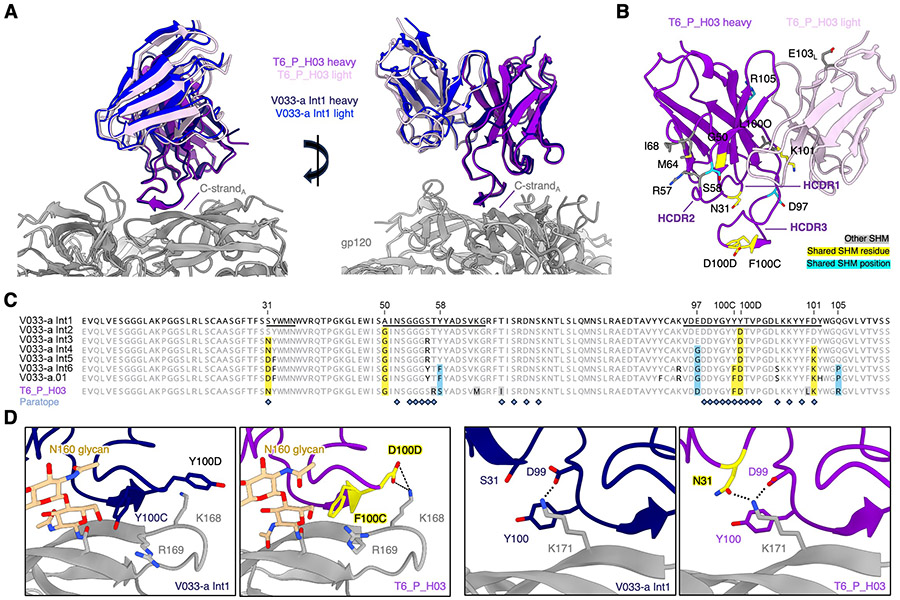
Structural basis of heterologous neutralization breadth for prime-derived antibody T6_P_H03. (**A**) Orthogonal views for the gp120 alignment of V033-a I1 and T6_P_H03 cryo-EM structures in complex with Env trimer. Only one Fab per trimer is shown for clarity. (**B**) Visualization of SHM residues in the T6_P_H03 Fab structure. Mutated residues are shown in stick representation and colored according to legend: yellow if the mutation is identical to one or more V033-a developmental intermediates; teal if only the mutated position is shared, not the resulting residue, with the V033-a developmental intermediates; and gray if the mutation if unique to T6_P_H03. The label for the site of light chain mutation is denoted with an “L” subscript, (**C**) Top; HC residue alignment of V033-a lineage developmental intermediates (I1–6), the matured broadly neutralizing antibody V033-a.01, and T6_P_H03. The V033-a I1 sequence is designated as the reference, and matching unmutated germline residues in all other sequences are depicted in light gray. The HCDRs are underlined in the I1 sequence. Conserved patterns of somatic hypermutation between T6_P_H03 and one or more of the developmental intermediates are highlighted similarly to panel B. Paratope residues from the Prime_H03 structure are designated with slate gray diamonds. (**D**) (Left) Comparison of interactions for HC paratope residues 100C and 100D in V033-a I1 and T6_P_H03. (Right) Comparison of C-strand K171 interactions for HC epitope residues 31, 99, and 100 in V033-a I1 and T6_P_H03. Yellow residues are from panel B and C.

To investigate the molecular basis for the observed heterologous neutralization breadth, we determined the structure of the T6_P_H03 antigen-binding fragment (Fab) in complex with Q23-APEX-GT1 using single particle cryogenic electron microscopy (cryo-EM) (Fig. 5, figs. S11, S15, Table S2). We previously found that, unlike many V2 apex bnAbs, the V033-a lineage can bind 3 Fabs per envelope trimer owing to (i) the ability to recognize monomeric gp120 and (ii) an HCDR3 which does not extend past the C-strand and into the middle of the envelope trimer, which would otherwise clash in the context of multiple Fabs (30, 58). Here, we obtained a 3D cryo-EM reconstruction of the T6_P_H03 complex that extended to 3.5 Å resolution and similarly revealed three Fabs bound to a single trimer, with an angle of approach and orientation nearly identical to V033a-UCA I1 (Fig. 5A, Fig. S11) (22). The elongated T6_P_H03 HCDR3 beta-strand precisely superimposed with that of V033a-UCA I1 and replicated the V033-a class-defining antiparallel strand-strand interaction with the V2 apex C-strand, resulting in the same three mainchain hydrogen bonds with the amide and carbonyl of R169 and the amide of K171. T6_P_H03 almost exclusively acquired HC SHM in response to Q23-APEX-GT1 immunization (12 HC residues vs. 1 LC residue) (Fig. 5B), which is consistent with the HC-dominated mode of recognition exhibited by the V033-a lineage (22, 30). T6_P_H03 had acquired SHM at 8 of 12 identical positions observed in the latest V033-a lineage developmental intermediate (V033-a.I6), half of which fell within the Fab interactive surface (Fig. 5C). SHM in 5 of 8 conserved positions yielded residues identical to one or more developmental intermediates (V033-a.I2-6), including 3 paratope residues that engaged the C-strand more favorably (Fig. 5D). V033a-UCA I1 utilizes two consecutive Tyr residues, Y100C and Y100D, to stabilize the extended aliphatic chains of C-strand residues R169 and K168, respectively. In contrast, T6_P_H03 acquired: F100C, which may be more sterically favorable since the hydroxyl group inserting between N156 and R169 is removed; and D100D, which forms a salt bridge with K168 (Fig. 5D, **left**). Further, both V033a-UCA I1 and T6_P_H03 engage the terminal amine group and extended aliphatic chain of C-strand residue K171 through D99 and Y100, respectively; however, T6_P_H03 acquired N31 through SHM, which forms an additional hydrogen-bond with K171 (Fig. 5D, **right)**.

Thus, we induced antibodies capable of binding autologous Env escape variants and neutralizing heterologous tier 2 viruses: a single Q23-APEX-GT1 immunization in mice recapitulated multiple immunogenetic and structural maturation pathways leading to breadth in the SHIV-elicited V033-a bnAb lineage.

### Homologous Q23-APEX-GT1 boosting increases serum neutralization breadth and on-track mutations

**Figure 6.**
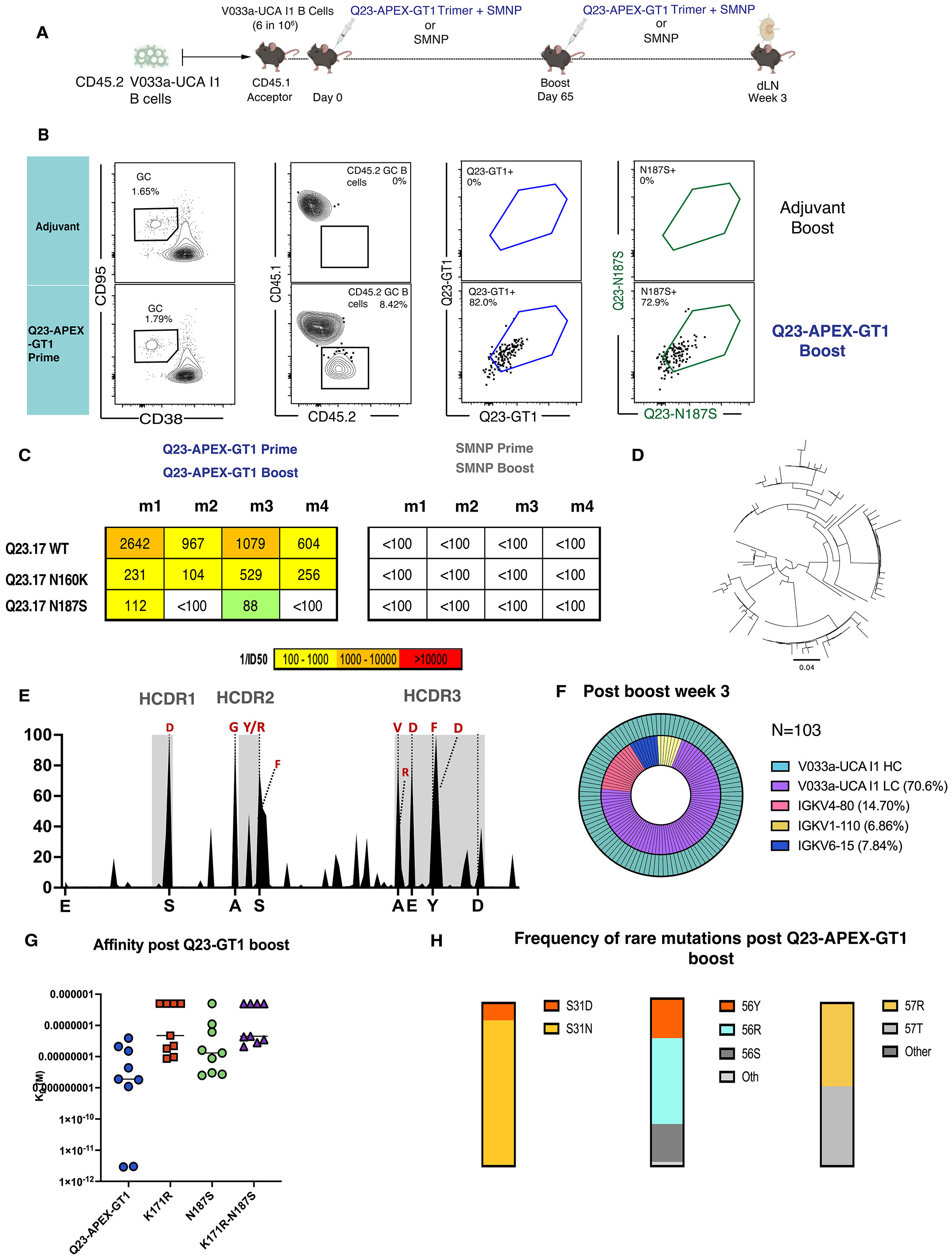
Homologous boosting leads to serum neutralization breadth and further on-track mutations. **(A**) Schematic presentation of mouse adoptive transfer and immunization experiments. WT CD45.1^+/+^ mice received V033a-UCA I1 B cells through intravenous transfer one day prior to immunization by Q23-APEX-GT1 trimer adjuvanted with SMNP. Day 65 post prime animals were boosted with Q23-APEX-GT1 adjuvanted with SMNP. Response was analyzed three weeks post immunization in dLN (n=4 mice per group, one experiment). (**B**) Representative FACS plots showing GC, CD45.2 B cells in GCs and their binding to Q23-APEX-GT1 and Q23-N187S during week three post-immunization with Q23-APEX-GT1 trimer with SMNP. Prime-boost with SMNP adjuvant without trimer was used as a control for protein immunizations, and it is shared with figure 7B. Major ticks mark log 10 scale. (**C**) Reverse ID50 of serum of animals receiving Q23-APEX-GT1 Prime and Q23-APEX-GT1 boost week 3 post immunization against Q23.17, Q23.17 N160K and Q23.17 N187S escape variant. (**D**) Divergence of KI V033a-UCA I1 HC post boost represented through phylogenetic tree to assess how heavy chains evolved after Q23-APEX-GT1 homologous boost. Tree Scale (0.04) indicates the number of substitutions per site. (**E**) HC mutation frequencies at 3 weeks post-immunization by V033a-UCA I1 KI HC. Selected mutations in V033a-UCA I1 HC present in mature V033 lineage bnAbs are represented in red. (**F**) HC (outer) and LC (inner) usage by single cells from Q23-APEX-GT1-specific CD45.2^+^ B cells sorted three weeks post-boost. (**G**) Affinities (K_D_ (M)) of antibodies (10 μg/ml) derived 6 weeks post-prime or 3 weeks post-boost against autologous Q23-APEX-GT1 Env and escape variant Envs (500 nM). (**H**) Frequency of selected rare mutations in HCDR1 and HCDR2 post Q23-APEX-GT1 trimer prime and Q23-APEX-GT1 Boost.

The broadly reactive response elicited by native-like Q23-APEX-GT1 trimer in these mice raised the possibility that a homologous boost might drive the acquisition of breadth and potency observed in the V033 macaque (22, 30), and potentially serum neutralization. We therefore delivered a homologous boost to the recipient CD45.1^+/+^ C57BL/6J (donor V033a-UCA I1 IgH^+/WT^ IgL^+/WT^ CD45.2^+/+^ B cells) mice 65 days after the initial priming with Q23-APEX-GT1; at week 3 post-boost, draining lymph nodes were analyzed (Fig. 6A). The homologous boost perpetuated the GCs and CD45.2 V033 B cells remained capable of binding both the priming immunogen as well as Q23-APEX-GT1 N187S, the next variant emerging from SHIV-Q23.17 escape in the V033-source (Fig. 6B) (22). We observed a 4.65- fold-increase in autologous Q23.17 serum neutralization and neutralization of an N160 glycan knockout variant after homologous boosting. Although we observed serum neutralization in one mouse, Q23.17N187S was not sensitive to neutralization by sera from most animals (Fig. 6C). Nonetheless, serum neutralization was enhanced relative to prime-only.

To understand the serum response, we examined SHM and affinity maturation in post-boost CD45.2^+^Ag^+^GC BCRs. As the prime-derived mAbs which could neutralize Q23.17 N187S with higher potency were overall better at neutralizing heterologous tier-2 viruses, we used Q23-APEX-GT1 N187S as a bait for sorting to enrich for potentially more potent mAbs. Sorted cells from homologous boosts showed further divergence of HC (Fig. 6D) and more on-track mutations than observed after priming alone (Fig. 6E). Sorted cells also showed a higher preference for endogenous mouse LCs than before (Fig. 6F). Relative to prime-derived mAbs, there was similar affinity maturation even after receiving a boost (Fig. 6G). Some ARMADiLLO-predicted mutations were found to increase, as well (Fig. 6H, Table S1). Thus, homologous boost-derived antibodies demonstrated broader serum neutralization and, in one instance, accommodated Q23.17 escape variants detected in the macaque, despite having never encountered them *in vivo*.

### Escape variant boosting leads to higher SHM, including on-track mutations

**Figure 7.**
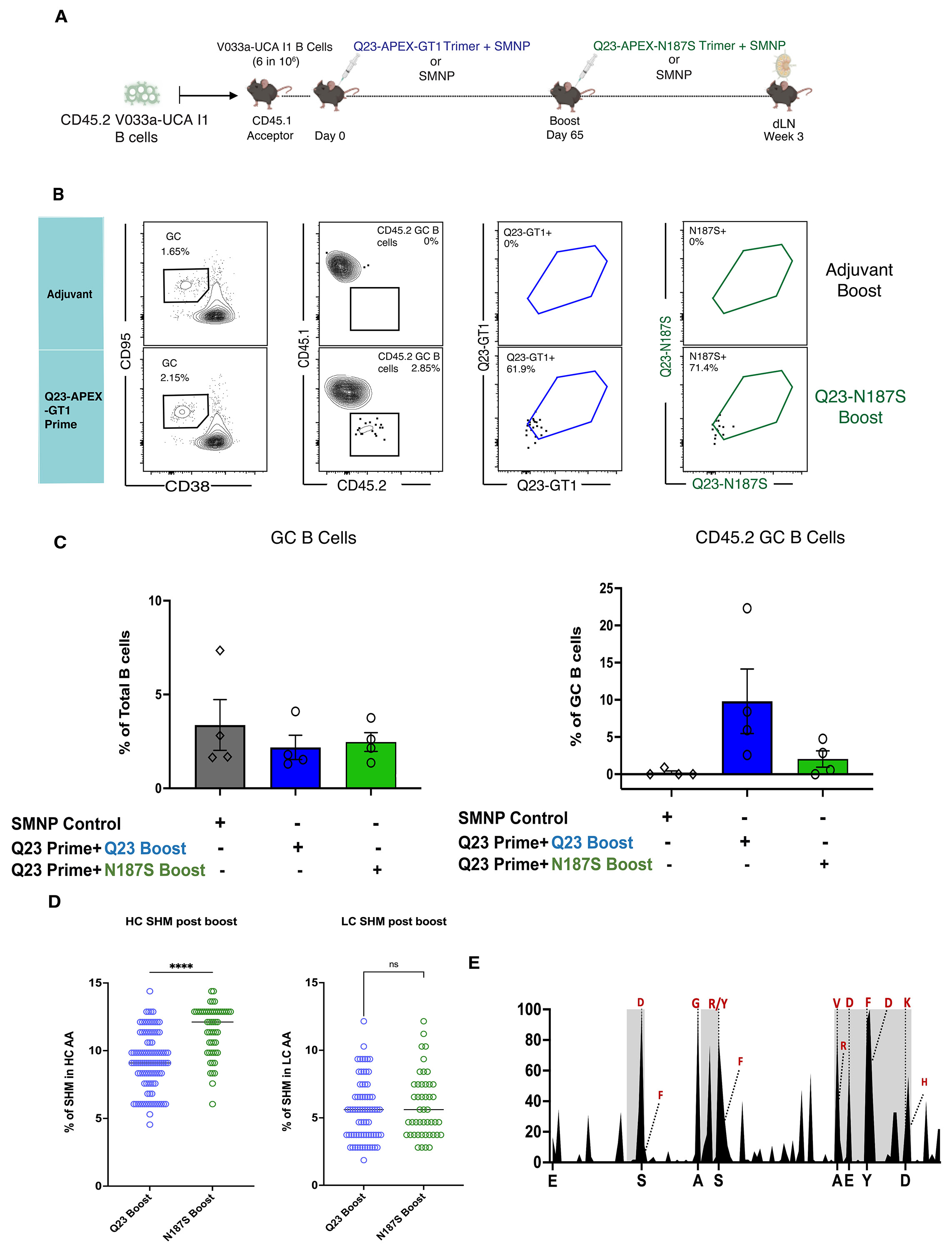
Escape-variant Env boosting leads to increased SHM and more on-track mutations. (**A**) Schematic presentation of mouse adoptive transfer and immunization experiments. Mice received V033a-UCA I1 B cells through intravenous transfer one day prior to immunization by Q23-APEX-GT1 trimer adjuvanted with SMNP. Prime-boost immunization by SMNP adjuvant without trimer was used as a control. Day 65 post-prime animals were boosted with Q23-APEX-GT1 N187S adjuvanted with SMNP. Response was analyzed 3 weeks post immunization in dLN. (**B**) Representative FACS plots showing GC, CD45.2 B cells in GCs and their binding to Q23-APEX-GT1 and Q23-APEX-GT1 N187S 3 weeks post-boost. Prime-boost with SMNP adjuvant without trimer was used as a control for protein immunizations, and it is repeated from figure 6B. Major ticks mark log 10 scale. (**C**) Quantification of GC B cells (left) and CD45.2 V033A-UCA I1 B cells in GCs (right) post-boost (n=4 mice per group, one experiment). (**D**) Total amino acid (AA) mutations in V033a-UCA I1 IGHV 3 weeks post-homologous (blue) or escape-variant (green) boost, compared with prime-only (gold) at weeks two, four, and six post-prime. Unpaired t test (two tailed) was performed. (**E**) V033a-UCA I1 KI HC mutation frequencies week three post-boost. Select mutations present in mature V033-lineage bnAbs are represented in red.

In natural infections, bnAbs emerge from coevolution between viral escape and host immune responses (5-8); we therefore investigated whether Env escape mutations identified *in vivo* could be incorporated into boosting immunogens to drive bnAb maturation (30, 58). In the post-prime GCs, V033a-UCA I1 B cells bound to the escape variant Q23-APEX-GT1 N187S from 4 wpi (fig. S9); we therefore immunized animals primed with Q23-APEX-GT1 with the escape variant Q23-APEX-GT1 N187S on day 65 (Fig. 7A). Serum neutralization of both autologous Q23.17 and escape variant N187S increased after Q23-APEX-GT1 N187S boost, whereas sensitivity to N160 glycan removal varied by animal (fig. S12A). Q23-APEX-GT1 N187S-boosted mice retained fewer CD45.2 B cells in the GC than those receiving a homologous Q23-APEX-GT1 boost (Fig. 7B-C). Cells were sorted with Q23-APEX-GT1 N187S probe to select for antibodies capable of binding the escape variants. HCs recovered after the Q23-APEX-GT1 N187S boost were more mutated than those arising from the Q23-APEX-GT1 boost (Fig. 7D). Hotspot analysis demonstrated that a rare HCDR3 lysine mutation, which was observed 6 wpi after prime, was enriched after variant-boosting but not homologous boosting (Fig. 7E, Table S1). BCRs from CD45.2^+^Ag^+^GC B cells isolated from mice post-escape variant boost used endogenous mouse LCs at low rates (fig. S12B). In LCs, the Q23-APEX-GT1 N187S boost did not increase overall mutation rates but did select for increased frequency of previously observed mutations as well as additional lineage-recapitulating mutations (fig. S12C-D). Thus, boosting with Q23-escape variants increased SHM overall, enriching on-track mutations.

### Both homologous and escape variant boosting enhance breadth and potency

**Figure 8.**
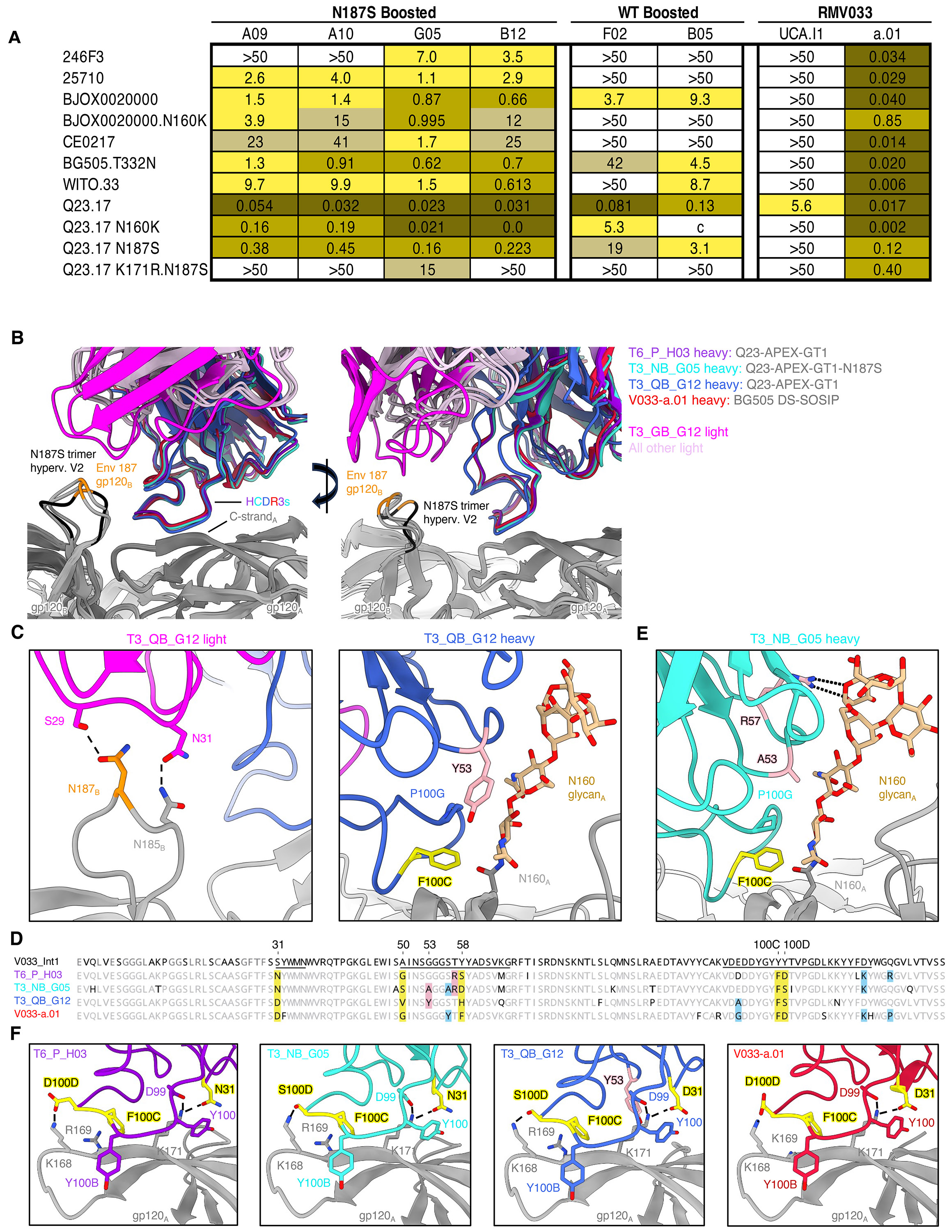
Neutralization breadth and potency of antibodies derived after homologous (Q23-APEX-GT1) or escape-variant (Q23-APEX-GT1 N187S) Env boost and its structural basis. (**A**) IC50 values of selected antibodies from 3 weeks post-boost and macaque mature V033 (RM V033-a.01) against autologous (Q23.17), Q23.17 N160K and escape variants (Q23.N187S, Q23.K171R.N187S Q23.R169E) and limited panel of 5 heterologous tier-2 HIV-1 strains. (**B**) Orthogonal views for the gp120 alignment of T6_P_H03, T3_NB_G05, T3_QB_G12, and V033-a.01 cryo-EM structures in complex with Env SOSIP trimers. Only one Fab per trimer is shown for clarity, and HCDR3 secondary structure removed for clarity. The position of hypervariable V2 loop residue 187 is highlighted in orange on the neighboring protomer_B_ for each structure. This loop is colored black in the mutant N187S trimer in complex with T3_NB_G05 to highlight its distinct conformation. (**C**) Unique T3_QB_G12 interactions with Env. (Left) The rotation of T3_QB_G12 positions its light chain proximal to hypervariable V2 loop on the adjacent protomer, resulting in interactions via LCDR1. T6_P_H03, T3_NB_G05, and V033-a.01 do not recognize this loop in their respective structures. (Right) Proposed explanation for the modest rotation of T3_QB_G12 relative to all other Fabs. The rotation of T3_QB_G12 Fab results from accommodation of the G53Y mutation in HCDR2 (pink) so it does not clash with N160 glycan on protomer_A_, which is larger than Gly or Ala residues present in all other Fabs. Select paratope residues are shown in stick representation and are colored according to panel C if they are a result of somatic hypermutation. Residues are labeled by Kabat numbering. (**D**) HC amino acid sequences of T6_P_H03, T3_NB_G05, T3_QB_G12, and V033-a.01 are aligned to the reference V033a UCA I1. Residues matching the reference are depicted in light gray and nonmatching residues are depicted in black. The HCDRs are underlined in the I1 sequence. Conserved positions of somatic hypermutation shared by all four antibodies are highlighted in yellow and mutated positions identical to V033-a.01, but only appearing in one or two murine antibodies, are highlighted in light blue. The unique mutated residue Y53 in T3_QB_G12 indicated in panel B is highlighted in pink. (**E**) Unique T3_NB_G05 interactions with N160 glycan. Select paratope residues are shown in stick representation and are colored according to panel C if they are a result of somatic hypermutation. (**F**) Comparison of C-strand interactions for each Fab. Interacting residues are shown in stick representation and are colored yellow or pink according to the Top alignment. HCDR3 secondary structure is removed to facilitate visualization. Residues are labeled by Kabat numbering.

To determine whether this overall increase in SHM was associated with a change in functionality, we expressed mAbs from animals receiving either homologous or variant boosts for pseudovirus neutralization assays. mAbs were selected based on either: 1) mutation frequency, 2) structure (CDRH3 stabilization), or 3) the presence of rare mutations. All boost-derived mAbs neutralized autologous Q23.17 virus and more potently neutralized Q23.17-N187S than mAbs derived post-prime (Fig. 8A). The neutralization ability of these antibodies was variably dependent on N160 glycan removal, but the antibodies were substantially more sensitive to C-strand mutations. Similarly, in a larger heterologous virus panel, Q23-APEX-GT1-boosted antibodies neutralized WT Q23.17 and a large panel of heterologous viruses with a higher potency than prime-derived antibodies (Fig. S13A). The Q23-APEX-GT1 N187S variant boost-derived G05 antibody showed slightly greater neutralization breadth but also greater potency (Fig. S13A). Boost-derived antibodies also demonstrated enhanced binding to Q23 escape variants, with the G05 mAb derived from the N187S boost being the most potent in both neutralization and binding (Fig. S13A-B). Thus, some mAbs derived post-boost displayed improved neutralization and trimer binding, and boosted animals displayed far higher serum neutralization than prime-only.

To provide molecular characterization of boost-derived antibodies, we determined the cryo-EM structures of homologous-boosted T3_QB_G12 Fab and variant-boosted T3_NB_G05 Fab in complex with their respective boosting Env trimers (Fig. 8B-F, fig. S14-15, Table S2). The 3D reconstruction densities for the T3_QB_G12 and T3_NB_G05 Fab-trimer complexes extended to 3.8 Å and 3.1 Å resolution, respectively, and revealed both boost-derived antibodies to bind 3 Fabs per trimer like other V033-a lineage members and variants (fig. S14) (30). While T3_NB_G05 recognized the V2-apex with an angle of approach similar to prime-derived T6_P_H03 and mature V033-a.01, T3_QB_G12 was modestly rotated and largely did not superimpose with the other Fab structures (Fig. 8B). As a result of this rotation, the T3_QB_G12 LC was positioned in close proximity to adjacent protomer B and recognized the hypervariable V2 loop through LCDR1 (Fig. 8C, **left**). We propose that T3_QB_G12 is rotated to prevent clashes between N160 glycan and SHM Y53 in HCDR2, which is larger than Gly or Ala residues present in all other Fabs and would provide stronger interactions by stacking against the first two *N*-acetylglucosamine residues of N160 glycanA (Fig. 8C, **right,** 8D). Despite this rotation of the T3_QB_G12 Fab body relative to others, the extended HCDR3 tips of each antibody aligned at the C-strand, likely owing to the conformational restraints imposed by the V033-a-class defining antiparallel mainchain hydrogen bonds with the C-strand, which were maintained in the boost-derived antibodies (Fig. 8B, fig. S14).

Similar to T3_QB_G12 Fab, T3_NB_G05 also acquired SHM within HCDR2 to increase interactions with N160 glycan_A_ (Fig. 8D-E). SHM residue A53 increases the hydrophobic interactive surface of the HCDR2 paratope over germline G53, while SHM R57 extends further than germline T53 and forms multiple hydrogen bonds with a terminal mannose residue. Boosting resulted in an increased frequency of antibodies bearing the improbable SHM residue K101 at the HCDR3 base, which is shared between V033-a.01, T3_NB_G05, and prime-derived antibody T6_P_H03; cryo-EM structures for each of these antibodies reveal K101 to interact with a terminal mannose residue on N156 glycan_A_ (Fig. S14). Despite the variations in glycan interactive surfaces for each Fab, conformations of apical glycans were nearly identical between V033-a.01 and each of the murine antibodies (Fig. S14). The remaining sites of HC SHM within the boost-derived antibody paratopes closely mirrored the pattern of maturation described for prime-derived antibody T6_P_H03 and was conserved with the mature rhesus bnAb V033-a.01, resulting in stronger interactions with C-strand residues K168 and K171 through conserved structural mechanisms (Fig. 8D, 8F). T3_QB_G12 and T3_NB_G05 each acquired LC SHM at just 2 of 7 identical positions acquired by V033-a.01, none of which fell within their respective interactive surfaces (fig. S14). However, in the context of V033a-UCA I1, introduction of each of these LC mutations individually improves neutralization of the autologous Q23.17 virus 2 to 3-fold (22), suggesting a role for stabilizing structural components of the Fab itself that may synergize with HC maturation to yield the modest increases in breadth and potency.

Together, these data suggest a single prime followed by a lineage-based boost mirroring natural co-evolution can elicit and shepherd the V033a-UCA I1 toward V033-like bnAbs, with several murine variants achieving neutralization breadth equal to or greater than the mature V033-a.01 antibody via reproducible structural solutions for improved V2-apex recognition.

## Discussion

BnAb induction remains a central goal for an effective HIV vaccine. Here, we leveraged a KI mouse model expressing a macaque bnAb precursor (V033a-UCA I1), isolated following SHIV-Q23.17 infection, to demonstrate that a stabilized germline-targeted Env trimer immunogen closely mimicking the native Env structure can effectively engage macaque BCRs. Strikingly, this near-germline V033-a B cell precursor required only a short maturation pathway to develop into a bona fide bnAb in macaques. A single immunization with adjuvanted Q23-APEX-GT1 protein trimer or Q23-APEX-GT1 saRNA encoding the membrane-bound trimer was sufficient to initiate on-track mutations in the V033a-UCA I1 lineage, closely mirroring those in the rhesus V033-a bnAb lineage, but in a shorter timeframe, and correlating with the emergence of neutralization breadth. Furthermore, boosting with a Q23 Env lineage-based escape variant differing by a single N187S mutation resulted in enhanced selection of breadth-conferring on-track mutations and further neutralization breadth and potency.

Inferred germline (iGL) or bnAb precursor KI models have been tremendously helpful in testing immunogens that target very rare human precursors (10, 13, 31, 60, 61). While knocking in unrearranged human immunoglobulin loci to allow for recombination is an attractive option (36, 62), the low number of total B cells in a mouse can make targeting desired precursor B cells extremely difficult (63). Knocking in a pre-rearranged BCR, in contrast, allows for a controlled titration of precursor frequency through adoptive transfer, as well as the opportunity to observe any recapitulation of known bnAb developmental pathways (48, 49, 61, 64). V033a-UCA I1 bridges murine and macaque preclinical models for HIV-1 vaccinology. Recent work (58) has demonstrated that bnAb induction may be genuinely translatable across these two model systems: there, using an immunogen similar to Q23-APEX-GT1 (two amino acid residues in the gp120 were modified to expand binding affinity for additional rhesus and human V2-apex bNAb UCAs), which consistently elicited V2-apex-epitope immunofocused antibody responses in outbred rhesus macaques. Importantly, priming of multiple diverse rhesus V2-apex-specific B cell lineages with long CDRH3-loops and desirable paratope properties was demonstrated. mAbs isolated from immunized macaques exhibited broad heterologous HIV trimer binding, similar to the observations made in our KI mice here. Furthermore, neutralization breadth was detected in plasma, and like our findings in V033 UCA KI mice, neutralization breadth in monkeys developed after a single homologous prime and boost immunization.

Compared to bnAbs targeting epitopes in HIV-1 Env such as CD4bs, V2-apex targeting bnAbs such as V033 do not require very high levels of SHM, but they do require a long HCDR3 loop (generated via rare VDJ recombination events) with a preference for C-strand anionic CDRH3 residues (22, 30, 39). We previously demonstrated that certain HIV Envs containing a naturally occurring glycan-sparse apex can readily bind to unmutated precursor versions of V2-apex bnAbs when presented in a near-native-like Env trimer configuration (28, 29, 39). Using macaque SHIV infection carrying HIV-1 transmitted/founder envelopes and other glycan-sparse Env, our group found that particular Env properties select for classes of V2 apex bnAb precursors which share binding approaches with human V2-apex targeting bnAbs (22, 30). In one of those studies, we observed that certain envelopes, such as Q23.17, have a greater propensity to induce V2 apex targeting bnAbs (22). Lineage tracing during macaque SHIV infection, therefore, informed both B cell lineage targeting and Env selection for immunogen design in this study.

Faithful preservation of structural and antigenic features of an epitope on target Env glycoproteins is essential to generate protective antibody responses (65). To design the Q23 Env lineage-based immunogens for evaluation in the V033a-UCA I1 KI mouse model, we utilized antibody-guided structure-based design to generate a prefusion-stabilized, native-like Q23 HIV Env trimer—Q23-APEX-GT1—with enhanced capacity to engage V2-apex bnAb precursors. In the absence of SHIV infection, which provides a constant source of evolving immunogen, escalating or interval immunizations can sustain GCs (66). The rapid maturation of macaque precursor V033a-UCA I1 in the absence of a replicating virus with a single immunization suggests that the features present in the early V033 lineage present a low barrier to developing into a bnAb. Notably, the rhesus line is characterized by the use of a D gene (D3-15*01); while this D gene lacks a human orthologue, it encodes an ‘EDDYG motif’ similar to the germline encoded YYD motif in several human V2-apex bnAbs, likely aiding in the development towards the bnAb (22, 58). While these germline-encoded EDDYG motifs are not uncommon in humans they are less common than the macaque equivalent (58, 67), and their potential contribution in human bnAb development remains underexplored. Furthermore, V033 is a less common V2-apex targeting antibody, in that it is trimer-preferring rather than trimer-specific. The binding of three Fabs to one trimer, as observed in the cryo-EM structure here, can enhance tensile strength during antigen extraction due to higher avidity during immunological synapse formation (68). Targeting similar precursors in humans may provide a route to a more tractable vaccination regimen.

Together, our data highlight that effective priming with a near native-like trimer may be the most critical step in eliciting V2 apex bnAbs. One caveat is that the D gene repertoire in humans differs from that of rhesus macaques. Overall, however, we provide proof-of-concept that using a near-native HIV Env-derived germline-targeting priming immunogen alongside lineage-guided variants could serve as a promising vaccine strategy for bnAb induction.

## Materials and Methods

### Study Design

The overarching concept of this study was to build on the extensive literature of human KI immunoglobulin mouse lines for HIV immunogen testing outlined in the Introduction by bringing strategies developed in NHP studies of SHIV infection to pre-clinical testing in a small animal model. The testing strategy is thus closely aligned to the existing approaches in Ig KI mice. Sample sizes were developed in reference to that field and particularly to prior work on V2-apex immunogens in Ig-KI mice (50, 69). Mice were assigned to groups randomly, and blinding was not performed. Replicates are listed in legends for the related experiments. ARRIVE guidelines consulted for animal reporting.

### Immunogen Design

To develop prefusion-stabilized, native-like Q23 Env trimers, a structure-guided engineering strategy was employed. Initial designs combined elements from previously described native flexibly linked (NFL) and repair-and-stabilization (RnS) scaffolds (40, 41), supplemented with additional gp120 and gp41 stabilizing mutations targeting conformational hotspots. Mutations were introduced to enhance apex hydrophilic packing, restrict CD4-induced rearrangements, and minimize exposure of non-neutralizing V3 epitopes. Constructs were designed as covalently linked single-chain trimers (SCTs) using a glycine-serine linker between gp120 and gp41. Core stabilizing mutations—including SOSIP (501C–605C, I559P) and a 201C–433C disulfide bond—were retained across all ten designs (Q23-SCT21 to Q23-SCT30). Subsequent variants introduced glycine substitutions (positions 569, 636), rigidity-enhancing mutations (Q551P), fusion peptide modifications (519R–520R), and RnS-derived residues to reinforce gp41. For gp120, select mutations (49E, 153E, 219A, 302Y, 320M, 334S) were incorporated based on structural analysis and computational modeling (ADROITrimer pipeline). Synthetic genes encoding the amino acid sequences for SCT21-30 (fig. S2) were ordered as gBlocks (IDT) and cloned in phCMV3 vector in-frame with tPA signal peptide with Gibson assembly.

### Stabilized Env Expression and Purification

Stabilized HIV Env trimers (SCT21–SCT30, fig. S2, and Q23-APEX-GT1 epitope mutants, fig. 4C, 6G, 8C) were expressed in HEK293F (Gibco, Cat# R79007) transfected with Env-encoding plasmids using PEI-MAX 4000 (Polysciences, Inc.). Four days post-transfection, supernatants were harvested and trimers purified via affinity chromatography using either GNL-agarose or PGT145-conjugated CNBr-activated Sepharose 4B beads (GE Healthcare). Eluates were further purified by size-exclusion chromatography (SEC) on a Superdex 200 10/300 GL column (GE Healthcare) and eluted in PBS or TBS.

### Biolayer Interferometry (BLI)

For antigenic screening of Q23 SCT immunogens, BLI was performed with Protein-A purified monoclonal antibodies (10 μg/ml)—that is, bnAb, nnAb panel from fig. S3 and all other antibodies isolated in this study. For assessing the polyclonal serum IgG response in immunized animals, purified polyclonal serum IgG (10 μg/ml) was used. IgGs were immobilized on ProA sensors (Sartorius) to a signal of 1.0 nM using an Octet Red96 instrument (ForteBio). The immobilized IgGs were then dipped in the running buffer (PBS, 0.1% BSA, 0.05% Tween20, pH 7.4) followed by 500 nm of redesigned Q23 trimers or running buffer. Following a 120 s association period, the tips were dipped into the running buffer and dissociation was measured for 240 s.

### Cell Surface Binding Assay

HEK293T cells (ATCC: CRL-3216) were seeded in T75 flasks and transfected with plasmid encoding Q23-APEX-GT1 using Lipofectamine 2000 (Thermo Fisher Scientific, Cat. #11668500), according to the manufacturer’s instructions. Forty-eight hours post-transfection, cells were harvested using FACS buffer (PBS supplemented with 2% FBS and 5 mM EDTA; Invitrogen, Cat. #15575-038), washed twice, and resuspended at 1 × 10^6^ cells/mL.

Cells were incubated with primary antibodies of interest (bnAbs, nnAbs in fig. S3) at a final concentration of 10 μg/mL for 1 hour at 4°C. After three washes with FACS buffer, cells were stained with PE-conjugated mouse anti-human IgG Fc secondary antibody (SouthernBiotech, Cat. #9040-09) for 1 hour at 4°C in the dark. Following additional washes, cells were resuspended in FACS buffer and analyzed on a Bio-Rad ZE5 flow cytometer. Data were processed using FlowJo software (BD Biosciences) (fig. S3E).

### Replicon RNA Synthesis and LNP Formulation

Env trimer coding sequences (Q23-APEX-GT1 and Q23-APEX-GT1 N187S) were cloned into a VEE-based replicon backbone. For replicon RNA synthesis, plasmid constructs were linearized via endonuclease digestion and purified with PureLink PCR Purification columns (ThermoFisher). Linearized plasmid DNA was transcribed via *in vitro* transcription using HiScribe T7 High Yield RNA Synthesis Kit (NEB), purified using PureLink RNA Mini columns (ThermoFisher), post-transcriptionally capped and methylated using ScriptCap Cap 1 Capping System (CellScript), and purified again using RNA Mini columns. To encapsulate replicons in LNPs, lipids including Heptadecan-9-yl 8-((2-hydroxyethyl)(6-oxo-6-(undecyloxy)hexyl)amino)octanoate (SM-102, Broadpharm), 1,2-dioctadecanoyl-sn-glycero-3-phosphocholine (DSPC, Avanti Polar Lipids), cholesterol (Chol, Avanti Polar Lipids), and 1,2-dimyristoyl-rac-glycero-3-methoxypolyethylene glycol-2000(PEG-DMG, Avanti Polar Lipids) were dissolved in ethanol at a 50:10:38.5:1.5 SM-102:DSPC:Chol:PEG-DMG molar ratio. RNA was dissolved in 10 mM citrate buffer pH 3, and then combined with the lipid solution at an N:P ratio of 6:1 (N, number of nitrogens on SM-102 ionizable lipid; P, number of phosphate groups on RNA) using an Ignite microfluidic mixing system (Precision Nanosystems) at a flow rate of 12 ml/min and a lipid:mRNA volume ratio of 3:1. The resulting replicon-loaded LNPs were dialyzed in 20 mM Tris-acetate and 8% (w/v) RNAse-free sucrose (VWR) using 3500 MWCO Slide-A-Lyzer cassettes (ThermoFisher) and stored at −80 °C till use.

SMNP adjuvant was prepared as previously described (51).

### Animals and Immunizations

10–12-week-old male CD45.1^+/+^ mice (B6.SJL-Ptprc^a^ Pepc^b^/BoyJ) were purchased from the Jackson Laboratory (Bar Harbor ME). Mice with B cells expressing a macaquized V033a-UCA I1 BCR were generated using the same CRISPR/Cas9 approach previously described for human KIs (48, 49). Mice were housed in a 12-hr light-dark cycle with ad libitum access to water and standard laboratory chow. All animal procedures were conducted in accordance with protocols 2016N000022 and 2016N000286 approved by the Institutional Animal Care and Use Committee (IACUC) at Mass General and conducted in accordance with the regulations of the Association for Assessment and Accreditation of Laboratory Animal Care (AAALAC) International. B cells from donor V033a-UCA I1 IgH^+/WT^ IgL^+/WT^ were isolated using a Pan B cell isolation kit II (Miltenyi Biotec, Bergisch Gladbach, Germany) and were adoptively transferred through tail vein injection into acceptor B6.SJL-Ptprca Pepcb/BoyJ CD45.1^+/+^ mice (Jackson Laboratory, Bar Harbor, ME, as above) at frequencies indicated in the text. Mice were immunized 24 hours post adoptive transfer. Groups of mice were injected bilaterally in the hind leg with Q23-APEX-GT1 LNPs at 2 μg total replicon dose (1 ug per injection site) or subcutaneously at the base of tail with 10 μg protein trimer antigen and 5 μg SMNP total (administered bilaterally with dose split equally between the two sites).

### Flow Cytometry

Lymph nodes were harvested from mice at weeks 2, 4, and 6 post-immunization or 3 weeks post boost as described in related figure legends. Draining inguinal lymph nodes were harvested from animals which had received SC immunizations. Iliac and popliteal lymph nodes were harvested from mice which received IM immunizations. Single-cell suspensions were prepared by gently crushing the draining lymph nodes from a single mouse together and passing them through a 70 μM strainer. Incubation with PBS containing Live/Dead Blue (Thermo Scientific, Waltham MA) diluted 500-fold and FcR Blocking reagent (Purified Rat anti-mouse CD16/CD32, BD Biosciences) diluted 200-fold was done for 20 mins at 4°C. After washing, BCR antigen staining was performed using biotinylated Q23-APEX-GT1 trimer conjugated to either streptavidin-BV510 (BioLegend, San Diego CA) or strepatvidin-Alexa647 (BioLegend) along with anti-mouse IgG1, IgG2a/2b and IgG3 (BUV805 clone X56, 2-40, R40-82) antibodies for 30 mins at 4°C. Excess antigen was washed off and rest of the cell surface staining was performed with an antibody cocktail containing CD4, CD8, F4/80, GR-1, NK1.1 (APC-eFluor 780, eBioscience, clone RM4-5, 53-6.7, BM8, RB6-8C5, PK136 respectively), B220 (BUV395, BD Bioscience, clone RA3-6B2), CD38 (BUV563, BD Biosciences, clone 90), CD95 (PE-Cy7, BioLegend, clone L138D7), CD45.1 (BV605, BioLegend, clone A20), CD45.2 (PE, BD Biosciences, clone 104), CD138 (BV650, BD Biosciences, clone 281-2), IgD (Alexa 594, Biolegend, 11-26c.2a clone) and IgM (BV750, II/41 clone) for 30 mins at 4°C. Flow cytometry data was acquired using BD FACS Symphony A5 cell analyzer. For cell sorting, Live/Dead stain was replaced with SYTOX Green (Thermo Fisher Scientific)/DAPI. The antibodies used for sorting were CD4, CD8, F4/80, GR-1 C NK1.1 (APC-eFluor 780, eBioscience, clone RM4-5, 53-6.7, BM8, RB6-8C5, PK136 respectively), B220 (Alex Fluor594 BioLegend, clone RA3-6B2), CD38 (BB700, BD Biosciences, clone 90), CD95 (PE-Cy7, BioLegend, clone L138D7), CD45.1 (APC R700, BD Biosciences, clone A20), CD45.2 (PE, BD Biosciences, clone 104), IgD (BV605, BioLegend, clone 11-26c.2a), CD138 (BV650, BD Biosciences, clone 281-2). Cells from each individual mouse were barcoded with TotalSeq^™^-C anti-mouse Hashtag Antibodies. A total of 10 hashtags were used. All fluorochrome labelled antibodies were used at a 1:200 dilution and hashtag antibodies were used at a 1:100 dilution. Cells were washed 3 times to remove any excess antibodies.

### Cell Sorting and Paired BCR Sequencing

Cells were sorted using BD FACSymphony S6 (BD Franklin Lakes, NJ) using 85 μM nozzle. Samples were sorted into PCR tubes containing PBS with 10% FBS. Encapsulation of sorted cells and NGS library preparation was performed following the 10x Genomics Chromium Next GEM Single Cell 5' Reagent Kits v2 protocol (10x Genomics). TapeStation Systems D5000 high sensitivity Screen Tape assay (Agilent, Santa Clara, CA) was used to measure library size. After quantifying the libraries through Qubit dsDNA High Sensitivity (Invitrogen, Waltham MA), they were pooled and were run on NextSeq 550 System (Illumina, San Diego, CA). Analysis was performed using the Cell Ranger v.6 software pipeline (10x Genomics, Pleasanton, CA) with a customized reference database. Sequencing data was analyzed using Geneious Prime and Biologics software (Geneious, Auckland, New Zealand) and IMGT/V-Quest (70-72). When PCR sequencing were not paired, that data was excluded from paired analysis.

### Cluster Analysis of Immunoglobulin Genes

Cluster similarity network was generated using Geneious Biologics (Geneious, Auckland, New Zealand). KI BCR has been indicated in the figures and shows the evolution of HCDR3 over time after a single bolus priming immunization. For boost with Q23-APEX-GT1, phylogenetic trees were generated for heavy chains in Geneious Prime by first aligning the sequences using Clustal Omega (73) and followed by tree building using RAxML (74) (using rapid hill climbing algorithm).

### Monoclonal Antibody Expression and Purification

Paired heavy and light chain plasmids for monoclonal antibodies cloned from immunized animals were co-transfected in Expi293 cells (Thermo Fisher Scientific, Waltham MA) in a 1:1 ratio using FectoPRO transfection reagent. Monoclonal IgGs were purified from the culture supernatant five days post-transfection with Protein-A Sepharose beads (Cytiva, Marlborough MA) per manufacturer’s instructions. After elution with IgG elution buffer (Thermo Fisher Scientific), antibodies were buffer exchanged into Tris buffered saline (TBS).

### Monoclonal Fab preparation for Cryo-EM structural studies

Monoclonal IgG Fab HC constructs were engineered by inserting a His-Avi tag followed by a stop codon upstream of the disulfide bond in the Fc region. Paired plasmids encoding the truncated heavy and light chains were co-transfected into Expi293 cells (Thermo Fisher Scientific, Cat# A14527) at a 1:1 ratio using FectoPRO transfection reagent (Polyplus, Cat# 116-001). At 24 hours post-transfection, cells were supplemented with 0.3 M valproic acid (Sigma, Cat# P4543-100G) and 40% glucose (Gibco, Cat# A2494001).

Five days after transfection, Fabs were purified from the culture supernatant using Ni Sepharose 6 Fast Flow resin (Cytiva, Cat# 17531802) following the manufacturer’s protocol. Eluted Fabs were buffer-exchanged into PBS and concentrated using a 10 kDa molecular weight cutoff centrifugal filter unit (Millipore, Cat# UFC905024). The concentrated Fab proteins were further purified by size exclusion chromatography on a Superdex 200 Increase 10/300 GL column (Sigma-Aldrich, Cat# GE28-9909-44). Fractions corresponding to the monomeric Fab peak were pooled, reconcentrated, and used for downstream Cryo-EM studies.

### Site-specific Glycosylation Analysis

To analyze glycosylation of the Q23-APEX-GT1 trimer, 100 μg of protein was denatured in 50 mM Tris/HCl (pH 8.0) with 6 M urea and 5 mM DTT for 1 hour, then alkylated with 20 mM iodoacetamide (IAA) in the dark for 1 hour. Residual IAA was quenched with 20 mM DTT. After buffer exchange into 50 mM Tris/HCl, the sample was split into three ~33 μg aliquots for separate overnight digestions with Trypsin, Chymotrypsin, or Alpha-lytic protease (1:30 w/w) at 37 °C. Peptides were desalted using Oasis HLB μElution plates and analyzed by nanoLC-ESI-MS using an Easy nLC 1200 coupled to an Orbitrap Eclipse with stepped HCD. Peptides were separated on an EasySpray PepMap RSLC C18 column with a 280-minute gradient. Key MS settings included a scan range of 300–2000 m/z, HCD energies of 15/25/45%, MS1 resolution of 120,000, and MS2 at 30,000. Glycopeptide data were processed in Byos (v5.5). True positives were confirmed by oxonium ions and matching b/y fragments. Searches used the Protein Metrics 305 N-glycan library with added sulfated glycans. Glycan abundance was calculated by chromatographic area comparison of glycoforms with identical peptide sequences. A 1% FDR was applied (4 ppm precursor, 10 ppm fragments), and all charge states were summed. Glycans were classified by composition: HexNAc(2)Hex(9–3) as M9–M3; HexNAc(2)Hex(10+) as M9Glc; fucosylated variants as FM; Hybrid types included HexNAc(3)Hex(5–6)X and Fhybrid; complex types were based on HexNAc count and fucosylation. Core glycans were <M3, and M9Glc–M4 were oligomannose. High-mannose included both oligomannose and hybrid types.

### Neutralization Assays

Neutralizing antibody assays were performed using TZM-bl indicator cells (NIH AIDS Reagent Program, Cat# 8129), as previously described (59). Briefly, TZM-bl cells were grown in cell culture medium (DMEM supplemented with 10% FBS and 1% penicillin/streptomycin), were plated in 96 well plates at a concentration of 1.5x104 cells/well and incubated at 37°C overnight. Serum samples were heat inactivated at 56°C for one hour, and serial 5-fold dilutions, starting at a dilution of 1:100, were made in cell culture media supplemented with 1% normal mouse serum (Avantor, Cat# 76226-474; supplied by Rockland Immunochemicals, original item# D108-00-0100). Normal mouse serum was included in the diluent to keep the concentration of test serum constant across wells. For monoclonal antibody neutralization assays, mAbs were serially diluted starting at a concentration of 50 μg/ml in cell culture media lacking normal mouse serum. Viruses were diluted to achieve a multiplicity of infection (MOI) of 0.3 upon addition to TZM-bl cells. Viruses were incubated with serum or antibody dilutions at 37°C for one hour, after which the virus-serum or virus-antibody mixtures were plated onto adherent TZM-bl cells and incubated at 37°C for 48 hours. Following incubation, cells were lysed with 0.5% Triton-X 100 in PBS and luciferase activity levels measured using the Promega luciferase assay system (Promega Cat# E1501) on a BioTek Synergy Neo2 plate reader (Agilent Technologies). Neutralization IC_50_ and ID_50_ values were calculated using Prism 10 software.

### Cryo-EM sample preparation and data collection

The structures of murine V033 variant Fabs in complex with envelope trimer were determined using single-particle cryo-EM. Samples were prepared by mixing each Fab with the cognate trimer used for terminal immunizations (T6_P_H03 with Q23-APEX-GT1; T3_NB_G05 with Q23-APEX-GT1 N187S; and T3_QB_HG12 with Q23-APEX-GT1) at a 2:1 Fab:protomer ratio and incubating on ice for 30 minutes. Each sample was then supplemented with PBS and the detergent Dodecyl β-D-maltoside (DDM; Anatrace) to achieve a final trimer concentration of ~2.5 mg/mL and final DDM concentration of 0.005% (w/v). 3 uL of each sample was then applied to a freshly glow-discharged (20 seconds at 20 mA) copper C-flat Holey carbon-coated grid (CF-1.2/1.3 300 mesh; EMS) within the chamber of a Vitrobot Mark IV at room temperature with 100% humidity. After incubating for 30 seconds, grids were blotted for 3 seconds prior to immediate plunge freezing in liquid ethane. Single-particle cryo-EM data were collected on a FEI Titan Krios cryo-transmission electron microscope operating at 300 kV and equipped with a Gatan K3 detector. Automated data collection was carried out using Leginon (75) in counting mode with a 105,000x magnification and a pixel size of 0.83 Å. The total dose of 58 e^−^/Å^2^ was fractionated over 50 raw frames, with defocus values set to cycle between −0.80 and −2.0 μm. Initial data collection attempts revealed prohibitive preferred orientation of the 3:1 Fab:trimer-bound complexes, therefore a 30-degree tilt was applied during collection of the final datasets used for each of the respective reconstructions.

### Cryo-EM data processing and model building

All processing was done in cryoSPARC v3.4 (76), including micrograph curation, motion correction, CTF estimation, particle picking, 2D classification, *ab initio* modeling, and 3D refinements. Particle picking was performed with non-templated blob picker. Multiple rounds of 2D classification, followed by iterative *ab initio* modeling and heterogenous refinements, were used to curate the particle datasets used in the final 3D reconstructions for each sample. All *ab initio* modeling and homogenous 3D refinements were performed using C1 symmetry, while all non-uniform 3D refinements of were performed using C3 symmetry. The initial coordinates for each murine V033 variant complex were obtained by docking the V033-a.01 Fab from PDB-9BNP and the Q23-APEX-GT2 trimer from PDB-9NVV into the present cryo-EM density using UCSF ChimeraX (77). Sequence corrections from the initial models to match each respective sample Fab and trimer were done manually in Coot (78). Each of the atomic models were solved by iterative real-space refinement in Phenix (79) and manual rebuilding in Coot. Overall structure quality was assessed using MolProbity (80) and EMRinger (81). Structural superimpositions of complexes were performed in UCSF ChimeraX. Final model statistics and validations are provided in fig. S15 and Table s2.

### Statistics

Significant differences between more than two groups were calculated using Kruskal-Wallis test and an unpaired t test was preformed to measure statistical differences between two groups. Statistics denoted throughout as no significant difference P > 0.05, *P < 0.05, **P < 0.01, ***P < 0.001, ****P < 0.0001. All P value analyses were calculated using Graphpad Prism Version 10.6.0 for statistical analysis.

## Supplementary Material

Supplemental Material **Supplementary Figure 1.** Further characterization of Q23-SCT immunogens, related to Figure 1.
**Supplementary Figure 2.** Construct sequences, related to Figure 1.
**Supplementary Figure 3.** Further characterization of Q23-SCT27 antigenicity and expression, related to Figure 1.
**Supplementary Figure 4.** Immunization with Q23-SCT after higher frequency of adoptive transfer leads to recruitment and activation of V033a-UCA I1 B cells, related to Figure 2.
**Supplementary Figure 5.** Sorting strategy of Ag+ V033a UCA I1 B cells post immunization. After gating on lymphocytes and gating out possible doublets, related to figures 3, 6, and 7.
**Supplementary Figure 6.** Macaque antibody features, related to Figure 4.
**Supplementary Figure 7.** Extended SHM analysis in immunized murine models, related to Figure 3.
**Supplementary Figure 8.** Affinity and neutralization breadth of week 4 prime derived antibodies, related to Figure 4.
**Supplementary Figure 9.** Q23-APEX-GT1 primed V033a-UCA I1 B cells can bind N187S escape variant, related to Figures 4 and 7.
**Supplementary Figure 10.** Priming leads to minimal serum neutralization, related to Figures 4 and 7.
**Supplementary Figure 11.** Select comparative structural features of V033a-UCA I1 and prime-derived antibody T6_P_H03
**Supplementary Figure 12.** Heterologous boost leads to expanded serum neutralization and LC usage in homologous vs N187S boost, related to Figures 6 and 7.
**Supplementary Figure 13.** Neutralization of Q23 and N187S boost-derived antibodies, related to Figures 6, 7, and 8.
**Supplementary Figure 14.** Select comparative structural features of V033-a lineage variants, related to Figures 5 and 8.
**Supplementary Figure 15.** Single-particle cryo-EM validation for murine V033-a antibodies in complex with HIV envelope, related to Figure 5.
**Supplementary Table 1.** Post-boost mutation frequencies.
**Supplementary Table 2.** Cryo-EM statistics.
**Supplementary Table 3.** Reagents used.

MDAR

Supplemental_Data_File_S1

## References

[1] Hemelaar J, The origin and diversity of the HIV-1 pandemic. Trends Mol. Med 18, 182–192 (2012). 22240486 10.1016/j.molmed.2011.12.001

[2] Burton DR, Poignard P, Stanfield RL, Wilson IA, Broadly neutralizing antibodies suggest new prospects to counter highly antigenically diverse viruses. Science 337, 183–186 (2012). 22798606 10.1126/science.1225416 PMC3600854

[3] Burton DR, Hangartner L, Broadly Neutralizing Antibodies to HIV and Their Role in Vaccine Design. Annu. Rev. Immunol 34, 635–659 (2016). 27168247 10.1146/annurev-immunol-041015-055515 PMC6034635

[4] Kwong PD, Mascola JR, HIV-1 Vaccines Based on Antibody Identification, B Cell Ontogeny, and Epitope Structure. Immunity 48, 855–871 (2018). 29768174 10.1016/j.immuni.2018.04.029

[5] Doria-Rose NA, Landais E, Coevolution of HIV-1 and broadly neutralizing antibodies. Curr. Opin. HIV AIDS 14, 286–293 (2019). 30994504 10.1097/COH.0000000000000550 PMC7553136

[6] Liao H-X, Lynch R, Zhou T, Gao F, Alam SM, Boyd SD, Fire AZ, Roskin KM, Schramm CA, Zhang Z, Zhu J, Shapiro L, Mullikin JC, Gnanakaran S, Hraber P, Wiehe K, Kelsoe G, Yang G, Xia S-M, Montefiori DC, Parks R, Lloyd KE, Scearce RM, Soderberg KA, Cohen M, Kamanga G, Louder MK, Tran LM, Chen Y, Cai F, Chen S, Moquin S, Du X, Joyce MG, Srivatsan S, Zhang B, Zheng A, Shaw GM, Hahn BH, Kepler TB, Korber BTM, Kwong PD, Mascola JR, Haynes BF, Co-evolution of a broadly neutralizing HIV-1 antibody and founder virus. Nature 4G6, 469–476 (2013). 10.1038/nature12053 PMC3637846 23552890

[7] Rantalainen K, Berndsen ZT, Murrell S, Cao L, Omorodion O, Torres JL, Wu M, Umotoy J, Copps J, Poignard P, Landais E, Paulson JC, Wilson IA, Ward AB, Co-evolution of HIV Envelope and Apex-Targeting Neutralizing Antibody Lineage Provides Benchmarks for Vaccine Design. Cell Rep. 23, 3249–3261 (2018). 29898396 10.1016/j.celrep.2018.05.046 PMC6019700

[8] Xiao X, Chen W, Feng Y, Zhu Z, Prabakaran P, Wang Y, Zhang M-Y, Longo NS, Dimitrov DS, Germline-like predecessors of broadly neutralizing antibodies lack measurable binding to HIV-1 envelope glycoproteins: Implications for evasion of immune responses and design of vaccine immunogens. Biochem. Biophys. Res. Commun 3G0, 404–409 (2009). 10.1016/j.bbrc.2009.09.029 PMC2787893 19748484

[9] Briney B, Sok D, Jardine JG, Kulp DW, Skog P, Menis S, Jacak R, Kalyuzhniy O, de Val N, Sesterhenn F, Le KM, Ramos A, Jones M, Saye-Francisco KL, Blane TR, Spencer S, Georgeson E, Hu X, Ozorowski G, Adachi Y, Kubitz M, Sarkar A, Wilson IA, Ward AB, Nemazee D, Burton DR, Schief WR, Tailored Immunogens Direct Affinity Maturation toward HIV Neutralizing Antibodies. Cell 166, 1459–1470.e11 (2016). 27610570 10.1016/j.cell.2016.08.005 PMC5018249

[10] Dosenovic P, von Boehmer L, Escolano A, Jardine J, Freund NT, Gitlin AD, McGuire AT, Kulp DW, Oliveira T, Scharf L, Pietzsch J, Gray MD, Cupo A, van Gils MJ, Yao K-H, Liu C, Gazumyan A, Seaman MS, Björkman PJ, Sanders RW, Moore JP, Stamatatos L, Schief WR, Nussenzweig MC, Immunization for HIV-1 Broadly Neutralizing Antibodies in Human Ig Knock-In Mice. Cell 161, 1505 (2015). 26091035 10.1016/j.cell.2015.06.003 PMC4604566

[11] Escolano A, Steichen JM, Dosenovic P, Kulp DW, Golijanin J, Sok D, Freund NT, Gitlin AD, Oliveira T, Araki T, Lowe S, Chen ST, Heinemann J, Yao K-H, Georgeson E, Saye-Francisco KL, Gazumyan A, Adachi Y, Kubitz M, Burton DR, Schief WR, Nussenzweig MC, Sequential Immunization Elicits Broadly Neutralizing Anti-HIV-1 Antibodies in Ig Knockin Mice. Cell 166, 1445–1458.e12 (2016). 27610569 10.1016/j.cell.2016.07.030 PMC5019122

[12] Jardine JG, Julien J-P, Menis S, Ota T, Kalyuzhniy O, McGuire A, Sok D, Huang P-S, MacPherson S, Jones M, Nieusma T, Mathison J, Baker D, Ward AB, Burton DR, Stamatatos L, Nemazee D, Wilson IA, Schief WR, Rational HIV Immunogen Design to Target Specific Germline B Cell Receptors. Science 340, 711–716 (2013). 23539181 10.1126/science.1234150 PMC3689846

[13] Jardine JG, Ota T, Sok D, Pauthner M, Kulp DW, Kalyuzhniy O, Skog PD, Thinnes TC, Bhullar D, Briney B, Menis S, Jones M, Kubitz M, Spencer S, Adachi Y, Burton DR, Schief WR, Nemazee D, Priming a broadly neutralizing antibody response to HIV-1 using a germline-targeting immunogen. Science 34G, 156–161 (2015). 10.1126/science.aac5894 PMC4669217 26089355

[14] Steichen JM, Kulp DW, Tokatlian T, Escolano A, Dosenovic P, Stanfield RL, McCoy LE, Ozorowski G, Hu X, Kalyuzhniy O, Briney B, Schiffner T, Garces F, Freund NT, Gitlin AD, Menis S, Georgeson E, Kubitz M, Adachi Y, Jones M, Mutafyan AA, Yun DS, Mayer CT, Ward AB, Burton DR, Wilson IA, Irvine DJ, Nussenzweig MC, Schief WR, HIV Vaccine Design to Target Germline Precursors of Glycan-Dependent Broadly Neutralizing Antibodies. Immunity 45, 483–496 (2016). 27617678 10.1016/j.immuni.2016.08.016 PMC5040827

[15] Caniels TG, Prabhakaran M, Ozorowski G, MacPhee KJ, Wu W, van der Straten K, Agrawal S, Derking R, Reiss EIMM, Millard K, Turroja M, Desrosiers A, Bethony J, Malkin E, Liesdek MH, van der Veen A, Klouwens M, Snitselaar JL, Bouhuijs JH, Bronson R, Jean-Baptiste J, Gajjala S, Rikhtegaran Tehrani Z, Benner A, Ramaswami M, Duff MO, Liu Y-W, Sato AH, Kim JY, Baken IJL, Mendes Silva C, Bijl TPL, van Rijswijk J, Burger JA, Cupo A, Yasmeen A, Phulera S, Lee W-H, Randall KN, Zhang S, Corcoran MM, Regadas I, Sullivan AC, Brown DM, Bohl JA, Greene KM, Gao H, Yates NL, Sawant S, Prins JM, Kootstra NA, Kaminsky SM, Barin B, Rahaman F, Meller M, Philiponis V, Laufer DS, Lombardo A, Mwoga L, Shotorbani S, Holman D, Koup RA, Klasse PJ, Karlsson Hedestam GB, Tomaras GD, van Gils MJ, Montefiori DC, McDermott AB, Hyrien O, Moore JP, Wilson IA, Ward AB, Diemert DJ, de Bree GJ, Andrews SF, Caskey M, Sanders RW, Precise targeting of HIV broadly neutralizing antibody precursors in humans. Science 0, eadv5572 (2025). 10.1126/science.adv5572 PMC12313413 40373114

[16] Willis JR, Prabhakaran M, Muthui M, Naidoo A, Sincomb T, Wu W, Cottrell CA, Landais E, deCamp AC, Keshavarzi NR, Kalyuzhniy O, Lee JH, Murungi LM, Ogonda WA, Yates NL, Corcoran MM, Phulera S, Musando J, Tsai A, Lemire G, Sein Y, Muteti M, Alamuri P, Bohl JA, Holman D, Himansu S, Leav B, Reuter C, Lin L-A, Ding B, He C, Straus WL, MacPhee KJ, Regadas I, Nyabundi DV, Chirchir R, Anzala O, Kimotho JN, Kibet C, Greene K, Gao H, Beatman E, Benson K, Laddy D, Brown DM, Bronson R, Jean-Baptiste J, Gajjala S, Rikhtegaran-Tehrani Z, Benner A, Ramaswami M, Lu D, Alavi N, Amirzehni S, Kubitz M, Tingle R, Georgeson E, Phelps N, Adachi Y, Liguori A, Flynn C, McKenney K, Zhou X, Owuor DC, Owuor SA, Kim S-Y, Duff M, Kim JY, Gibson G, Baboo S, Diedrich J, Schiffner T, Shields M, Matsoso M, Santos J, Syvertsen K, Kennedy A, Schroeter M, Vekemans J, Yates JR, Paulson JC, Hyrien O, McDermott AB, Maenetje P, Nyombayire J, Karita E, Ingabire R, Edward V, Muturi-Kioi V, Maenza J, Shapiro AE, McElrath MJ, Edupuganti S, Taylor BS, Diemert D, Ozorowski G, Koup RA, Montefiori D, Ward AB, Hedestam GBK, Tomaras G, Hunt DJ, Muema D, Sok D, Laufer DS, Andrews SF, Nduati EW, Schief WR, Vaccination with mRNA-encoded nanoparticles drives early maturation of HIV bnAb precursors in humans. Science 0, eadr8382 (2025). 10.1126/science.adr8382 PMC13164876 40373112

[17] Leggat DJ, Cohen KW, Willis JR, Fulp WJ, deCamp AC, Kalyuzhniy O, Cottrell CA, Menis S, Finak G, Ballweber-Fleming L, Srikanth A, Plyler JR, Schiffner T, Liguori A, Rahaman F, Lombardo A, Philiponis V, Whaley RE, Seese A, Brand J, Ruppel AM, Hoyland W, Yates NL, Williams LD, Greene K, Gao H, Mahoney CR, Corcoran MM, Cagigi A, Taylor A, Brown DM, Ambrozak DR, Sincomb T, Hu X, Tingle R, Georgeson E, Eskandarzadeh S, Alavi N, Lu D, Mullen T-M, Kubitz M, Groschel B, Maenza J, Kolokythas O, Khati N, Bethony J, Crotty S, Roederer M, Karlsson Hedestam GB, Tomaras GD, Montefiori D, Diemert D, Koup RA, Laufer DS, McElrath MJ, McDermott AB, Schief WR, Vaccination induces HIV broadly neutralizing antibody precursors in humans. Science 378, eadd6502 (2022). 36454825 10.1126/science.add6502 PMC11103259

[18] Zhou T, Zhu J, Wu X, Moquin S, Zhang B, Acharya P, Georgiev IS, Altae-Tran HR, Chuang G-Y, Joyce MG, Kwon YD, Longo NS, Louder MK, Luongo T, McKee K, Schramm CA, Skinner J, Yang Y, Yang Z, Zhang Z, Zheng A, Bonsignori M, Haynes BF, Scheid JF, Nussenzweig MC, Simek M, Burton DR, Koff WC, Mullikin JC, Connors M, Shapiro L, Nabel GJ, Mascola JR, Kwong PD, Multi-donor Analysis Reveals Structural Elements, Genetic Determinants, and Maturation Pathway for Effective HIV-1 Neutralization by VRCO1-class Antibodies. Immunity 3G, 245–258 (2013). 10.1016/j.immuni.2013.04.012 PMC3985390 23911655

[19] Landais E, Huang X, Havenar-Daughton C, Murrell B, Price MA, Wickramasinghe L, Ramos A, Bian CB, Simek M, Allen S, Karita E, Kilembe W, Lakhi S, Inambao M, Kamali A, Sanders EJ, Anzala O, Edward V, Bekker L-G, Tang J, Gilmour J, Kosakovsky-Pond SL, Phung P, Wrin T, Crotty S, Godzik A, Poignard P, Broadly Neutralizing Antibody Responses in a Large Longitudinal Sub-Saharan HIV Primary Infection Cohort. PLOS Pathog. 12, e1005369 (2016). 26766578 10.1371/journal.ppat.1005369 PMC4713061

[20] Rusert P, Kouyos RD, Kadelka C, Ebner H, Schanz M, Huber M, Braun DL, Hozé N, Scherrer A, Magnus C, Weber J, Uhr T, Cippa V, Thorball CW, Kuster H, Cavassini M, Bernasconi E, Hoffmann M, Calmy A, Battegay M, Rauch A, Yerly S, Aubert V, Klimkait T, Böni J, Fellay J, Regoes RR, Günthard HF, Trkola A, Swiss HIV Cohort Study, Determinants of HIV-1 broadly neutralizing antibody induction. Nat. Med 22, 1260–1267 (2016). 27668936 10.1038/nm.4187

[21] Walker LM, Simek MD, Priddy F, Gach JS, Wagner D, Zwick MB, Phogat SK, Poignard P, Burton DR, A limited number of antibody specificities mediate broad and potent serum neutralization in selected HIV-1 infected individuals. PLoS Pathog. 6, e1001028 (2010). 20700449 10.1371/journal.ppat.1001028 PMC2916884

[22] Habib R, Roark RS, Li H, Connell AJ, Hogarty MP, Wagh K, Wang S, Marchitto L, Skelly AN, Carey JW, Sowers KJ, Ayyanathan K, Plante SJ, Bibollet-Ruche F, Park Y, Agostino CJ, Singh A, Martella CL, Lewis E, Lora J, Ding W, Campion MS, Zhao C, Liu W, Li Y, Li X, Liang B, Chowdhury RR, Amereh K, Itallie EV, Sheng Z, Ghosh AR, Bar KJ, Williams WB, Wiehe K, Saunders KO, Edwards RJ, Cain DW, Lewis M, Batista FD, Burton DR, Andrabi R, Kulp DW, Haynes BF, Korber B, Shapiro L, Kwong PD, Hahn BH, Shaw GM, Env-antibody coevolution identifies B cell priming as the principal bottleneck to HIV-1 V2 apex broadly neutralizing antibody development. Sci. Immunol (unpublished). 10.1126/sciimmunol.adz3933 PMC13015429 41686912

[23] Bhiman JN, Anthony C, Doria-Rose NA, Karimanzira O, Schramm CA, Khoza T, Kitchin D, Botha G, Gorman J, Garrett NJ, Abdool Karim SS, Shapiro L, Williamson C, Kwong PD, Mascola JR, Morris L, Moore PL, Viral variants that initiate and drive maturation of V1V2-directed HIV-1 broadly neutralizing antibodies. Nat. Med 21, 1332–1336 (2015). 26457756 10.1038/nm.3963 PMC4637988

[24] Doria-Rose NA, Schramm CA, Gorman J, Moore PL, Bhiman JN, DeKosky BJ, Ernandes MJ, Georgiev IS, Kim HJ, Pancera M, Staupe RP, Altae-Tran HR, Bailer RT, Crooks ET, Cupo A, Druz A, Garrett NJ, Hoi KH, Kong R, Louder MK, Longo NS, McKee K, Nonyane M, O’Dell S, Roark RS, Rudicell RS, Schmidt SD, Sheward DJ, Soto C, Wibmer CK, Yang Y, Zhang Z, Mullikin JC, Binley JM, Sanders RW, Wilson IA, Moore JP, Ward AB, Georgiou G, Williamson C, Karim SSA, Morris L, Kwong PD, Shapiro L, Mascola JR, Developmental pathway for potent V1V2-directed HIV-neutralizing antibodies. Nature 50G, 55–62 (2014). 10.1038/nature13036 PMC4395007 24590074

[25] Landais E, Murrell B, Briney B, Murrell S, Rantalainen K, Berndsen ZT, Ramos A, Wickramasinghe L, Smith ML, Eren K, de Val N, Wu M, Cappelletti A, Umotoy J, Lie Y, Wrin T, Algate P, Chan-Hui P-Y, Karita E, IAVI Protocol C Investigators, IAVI African HIV Research Network, Ward AB, Wilson IA, Burton DR, Smith D, Pond SLK, Poignard P, HIV Envelope Glycoform Heterogeneity and Localized Diversity Govern the Initiation and Maturation of a V2 Apex Broadly Neutralizing Antibody Lineage. Immunity 47, 990–1003.e9 (2017). 29166592 10.1016/j.immuni.2017.11.002 PMC5736302

[26] Poss M, Overbaugh J, Variants from the diverse virus population identified at seroconversion of a clade A human immunodeficiency virus type 1-infected woman have distinct biological properties. J. Virol 73, 5255–5264 (1999). 10364271 10.1128/jvi.73.7.5255-5264.1999 PMC112580

[27] Bonsignori M, Hwang K-K, Chen X, Tsao C-Y, Morris L, Gray E, Marshall DJ, Crump JA, Kapiga SH, Sam NE, Sinangil F, Pancera M, Yongping Y, Zhang B, Zhu J, Kwong PD, O’Dell S, Mascola JR, Wu L, Nabel GJ, Phogat S, Seaman MS, Whitesides JF, Moody MA, Kelsoe G, Yang X, Sodroski J, Shaw GM, Montefiori DC, Kepler TB, Tomaras GD, Alam SM, Liao H-X, Haynes BF, Analysis of a Clonal Lineage of HIV-1 Envelope V2/V3 Conformational Epitope-Specific Broadly Neutralizing Antibodies and Their Inferred Unmutated Common Ancestors. J. Virol 85, 9998–10009 (2011). 21795340 10.1128/JVI.05045-11 PMC3196428

[28] Gorman J, Soto C, Yang MM, Davenport TM, Guttman M, Bailer RT, Chambers M, Chuang G-Y, DeKosky BJ, Doria-Rose NA, Druz A, Ernandes MJ, Georgiev IS, Jarosinski MC, Joyce MG, Lemmin TM, Leung S, Louder MK, McDaniel JR, Narpala S, Pancera M, Stuckey J, Wu X, Yang Y, Zhang B, Zhou T, Program NCS, Mullikin JC, Baxa U, Georgiou G, McDermott AB, Bonsignori M, Haynes BF, Moore PL, Morris L, Lee KK, Shapiro L, Mascola JR, Kwong PD, Structures of HIV-1 Env V1V2 with broadly neutralizing antibodies reveal commonalities that enable vaccine design. Nat. Struct. Mol. Biol 23, 81–90 (2016). 26689967 10.1038/nsmb.3144 PMC4833398

[29] Voss JE, Andrabi R, McCoy LE, De Val N, Fuller RP, Messmer T, Su C-Y, Sok D, Khan SN, Garces F, Pritchard LK, Wyatt RT, Ward AB, Crispin M, Wilson IA, Burton DR, Elicitation of Neutralizing Antibodies Targeting the V2 Apex of the HIV Envelope Trimer in a Wild-Type Animal Model. Cell Rep. 21, 222–235 (2017). 28978475 10.1016/j.celrep.2017.09.024 PMC5640805

[30] Roark RS, Habib R, Gorman J, Li H, Connell AJ, Bonsignori M, Guo Y, Hogarty MP, Olia AS, Sowers K, Zhang B, Bibollet-Ruche F, Callaghan S, Carey JW, Cerutti G, Harris DR, He W, Lewis E, Liu T, Mason RD, Park Y, Rando JM, Singh A, Wolff J, Lei QP, Louder MK, Doria-Rose NA, Andrabi R, Saunders KO, Seaman MS, Haynes BF, Kulp DW, Mascola JR, Roederer M, Sheng Z, Hahn BH, Shaw GM, Kwong PD, Shapiro L, HIV-1 neutralizing antibodies in SHIV-infected macaques recapitulate structurally divergent modes of human V2 apex recognition with a single D gene. bioRxiv [Preprint] (2024). 10.1101/2024.06.11.598384.

[31] Huang D, Abbott RK, Havenar-Daughton C, Skog PD, Al-Kolla R, Groschel B, Blane TR, Menis S, Tran JT, Thinnes TC, Volpi SA, Liguori A, Schiffner T, Villegas SM, Kalyuzhniy O, Pintea M, Voss JE, Phelps N, Tingle R, Rodriguez AR, Martin G, Kupryianov S, deCamp A, Schief WR, Nemazee D, Crotty S, B cells expressing authentic naive human VRC01-class BCRs can be recruited to germinal centers and affinity mature in multiple independent mouse models. Proc. Natl. Acad. Sci 117, 22920–22931 (2020). 32873644 10.1073/pnas.2004489117 PMC7502816

[32] Ray R, Schiffner T, Wang X, Yan Y, Rantalainen K, Lee C-CD, Parikh S, Reyes RA, Dale GA, Lin Y-C, Pecetta S, Giguere S, Swanson O, Kratochvil S, Melzi E, Phung I, Madungwe L, Kalyuzhniy O, Warner J, Weldon SR, Tingle R, Lamperti E, Kirsch KH, Phelps N, Georgeson E, Adachi Y, Kubitz M, Nair U, Crotty S, Wilson IA, Schief WR, Batista FD, Affinity gaps among B cells in germinal centers drive the selection of MPER precursors. Nat. Immunol, 1–14 (2024). 10.1038/s41590-024-01844-7 PMC11147770 38816616

[33] Chen X, Zhou T, Schmidt SD, Duan H, Cheng C, Chuang G-Y, Gu Y, Louder MK, Lin BC, Shen C-H, Vaccination induces maturation in a mouse model of diverse unmutated VRC01-class precursors to HIV-neutralizing antibodies with> 50% breadth. Immunity 54, 324–339. e8 (2021). 33453152 10.1016/j.immuni.2020.12.014 PMC8020832

[34] Cottrell CA, Hu X, Lee JH, Skog P, Luo S, Flynn CT, McKenney KR, Hurtado J, Kalyuzhniy O, Liguori A, Willis JR, Landais E, Raemisch S, Chen X, Baboo S, Himansu S, Diedrich JK, Duan H, Cheng C, Schiffner T, Bader DLV, Kulp DW, Tingle R, Georgeson E, Eskandarzadeh S, Alavi N, Lu D, Sincomb T, Kubitz M, Mullen T-M, Yates JR, Paulson JC, Mascola JR, Alt FW, Briney B, Sok D, Schief WR, Heterologous prime-boost vaccination drives early maturation of HIV broadly neutralizing antibody precursors in humanized mice. Sci. Transl. Med 16, eadn0223 (2024). 38753806 10.1126/scitranslmed.adn0223 PMC11233128

[35] Haynes BF, Wiehe K, Borrow P, Saunders KO, Korber B, Wagh K, McMichael AJ, Kelsoe G, Hahn BH, Alt F, Shaw GM, Strategies for HIV-1 vaccines that induce broadly neutralizing antibodies. Nat. Rev. Immunol 23, 142–158 (2023). 35962033 10.1038/s41577-022-00753-w PMC9372928

[36] Tian M, Cheng C, Chen X, Duan H, Cheng H-L, Dao M, Sheng Z, Kimble M, Wang L, Lin S, Schmidt SD, Du Z, Joyce MG, Chen Y, DeKosky BJ, Chen Y, Normandin E, Cantor E, Chen RE, Doria-Rose NA, Zhang Y, Shi W, Kong W-P, Choe M, Henry AR, Laboune F, Georgiev IS, Huang P-Y, Jain S, McGuire AT, Georgeson E, Menis S, Douek DC, Schief WR, Stamatatos L, Kwong PD, Shapiro L, Haynes BF, Mascola JR, Alt FW, Induction of HIV Neutralizing Antibody Lineages in Mice with Diverse Precursor Repertoires. Cell 166, 1471–1484.e18 (2016). 27610571 10.1016/j.cell.2016.07.029 PMC5103708

[37] Wang X, Cottrell CA, Hu X, Ray R, Bottermann M, Villavicencio PM, Yan Y, Xie Z, Warner JE, Ellis-Pugh JR, mRNA-LNP prime boost evolves precursors toward VRC01-like broadly neutralizing antibodies in preclinical humanized mouse models. Sci. Immunol 9, eadn0622 (2024). 38753808 10.1126/sciimmunol.adn0622 PMC11488661

[38] Xie Z, Lin Y-C, Steichen JM, Ozorowski G, Kratochvil S, Ray R, Torres JL, Liguori A, Kalyuzhniy O, Wang X, Warner JE, Weldon SR, Dale GA, Kirsch KH, Nair U, Baboo S, Georgeson E, Adachi Y, Kubitz M, Jackson AM, Richey ST, Volk RM, Lee JH, Diedrich JK, Prum T, Falcone S, Himansu S, Carfi A, Yates JR 3rd, Paulson JC, Sok D, Ward AB, Schief WR, Batista FD, mRNA-LNP HIV-1 trimer boosters elicit precursors to broad neutralizing antibodies. Science 384, eadk0582 (2024). 38753770 10.1126/science.adk0582 PMC11488660

[39] Andrabi R, Voss JE, Liang C-H, Briney B, McCoy LE, Wu C-Y, Wong C-H, Poignard P, Burton DR, Identification of common features in prototype broadly neutralizing antibodies to HIV envelope V2 apex to facilitate vaccine design. Immunity 43, 959–973 (2015). 26588781 10.1016/j.immuni.2015.10.014 PMC4654981

[40] Rutten L, Lai Y-T, Blokland S, Truan D, Bisschop IJM, Strokappe NM, Koornneef A, van Manen D, Chuang G-Y, Farney SK, Schuitemaker H, Kwong PD, Langedijk JPM, A Universal Approach to Optimize the Folding and Stability of Prefusion-Closed HIV-1 Envelope Trimers. Cell Rep. 23, 584–595 (2018). 29642014 10.1016/j.celrep.2018.03.061 PMC6010203

[41] Sharma SK, de Val N, Bale S, Guenaga J, Tran K, Feng Y, Dubrovskaya V, Ward AB, Wyatt RT, Cleavage-independent HIV-1 Env trimers engineered as soluble native spike mimetics for vaccine design. Cell Rep. 11, 539–550 (2015). 25892233 10.1016/j.celrep.2015.03.047 PMC4637274

[42] Sanders RW, Derking R, Cupo A, Julien J-P, Yasmeen A, de Val N, Kim HJ, Blattner C, de la Peña AT, Korzun J, Golabek M, de Los Reyes K, Ketas TJ, van Gils MJ, King CR, Wilson IA, Ward AB, Klasse PJ, Moore JP, A next-generation cleaved, soluble HIV-1 Env trimer, BG505 SOSIP.664 gp140, expresses multiple epitopes for broadly neutralizing but not non-neutralizing antibodies. PLoS Pathog. 9, e1003618 (2013). 24068931 10.1371/journal.ppat.1003618 PMC3777863

[43] Zhang P, Gorman J, Geng H, Liu Q, Lin Y, Tsybovsky Y, Go EP, Dey B, Andine T, Kwon A, Patel M, Gururani D, Uddin F, Guzzo C, Cimbro R, Miao H, McKee K, Chuang G-Y, Martin L, Sironi F, Malnati MS, Desaire H, Berger EA, Mascola JR, Dolan MA, Kwong PD, Lusso P, Interdomain Stabilization Impairs CD4 Binding and Improves Immunogenicity of the HIV-1 Envelope Trimer. Cell Host Microbe 23, 832–844.e6 (2018). 29902444 10.1016/j.chom.2018.05.002 PMC6007033

[44] Guenaga J, Garces F, de Val N, Stanfield RL, Dubrovskaya V, Higgins B, Carrette B, Ward AB, Wilson IA, Wyatt RT, Glycine Substitution at Helix-to-Coil Transitions Facilitates the Structural Determination of a Stabilized Subtype C HIV Envelope Glycoprotein. Immunity 46, 792–803.e3 (2017). 28514686 10.1016/j.immuni.2017.04.014 PMC5439057

[45] Yang L, Sharma SK, Cottrell C, Guenaga J, Tran K, Wilson R, Behrens A-J, Crispin M, de Val N, Wyatt RT, Structure-Guided Redesign Improves NFL HIV Env Trimer Integrity and Identifies an Inter-Protomer Disulfide Permitting Post-Expression Cleavage. Front. Immunol 9, 1631 (2018). 30065725 10.3389/fimmu.2018.01631 PMC6056610

[46] Guenaga J, Dubrovskaya V, de Val N, Sharma SK, Carrette B, Ward AB, Wyatt RT, Structure-Guided Redesign Increases the Propensity of HIV Env To Generate Highly Stable Soluble Trimers. J. Virol 90, 2806–2817 (2015). 26719252 10.1128/JVI.02652-15 PMC4810649

[47] Rawi R, Rutten L, Lai Y-T, Olia AS, Blokland S, Juraszek J, Shen C-H, Tsybovsky Y, Verardi R, Yang Y, Zhang B, Zhou T, Chuang G-Y, Kwong PD, Langedijk JPM, Automated Design by Structure-Based Stabilization and Consensus Repair to Achieve Prefusion-Closed Envelope Trimers in a Wide Variety of HIV Strains. Cell Rep. 33, 108432 (2020). 33238130 10.1016/j.celrep.2020.108432 PMC7714614

[48] Lin Y-C, Pecetta S, Steichen JM, Kratochvil S, Melzi E, Arnold J, Dougan SK, Wu L, Kirsch KH, Nair U, Schief WR, Batista FD, One-step CRISPR/Cas9 method for the rapid generation of human antibody heavy chain knock-in mice. EMBO J. 37, e99243 (2018). 30087111 10.15252/embj.201899243 PMC6138433

[49] Wang X, Ray R, Kratochvil S, Melzi E, Lin Y-C, Giguere S, Xu L, Warner J, Cheon D, Liguori A, Groschel B, Phelps N, Adachi Y, Tingle R, Wu L, Crotty S, Kirsch KH, Nair U, Schief WR, Batista FD, Multiplexed CRISPR/CAS9-mediated engineering of pre-clinical mouse models bearing native human B cell receptors. EMBO J. 40, e105926 (2021). 33258500 10.15252/embj.2020105926 PMC7809789

[50] Melzi E, Willis JR, Ma KM, Lin Y-C, Kratochvil S, Berndsen ZT, Landais EA, Kalyuzhniy O, Nair U, Warner J, Steichen JM, Kalyuzhniy A, Le A, Pecetta S, Perez M, Kirsch K, Weldon SR, Falcone S, Himansu S, Carfi A, Sok D, Ward AB, Schief WR, Batista FD, Membrane-bound mRNA immunogens lower the threshold to activate HIV Env V2 apex-directed broadly neutralizing B cell precursors in humanized mice. Immunity 55, 2168–2186.e6 (2022). 36179690 10.1016/j.immuni.2022.09.003 PMC9671093

[51] Silva M, Kato Y, Melo MB, Phung I, Freeman BL, Li Z, Roh K, Wijnbergen JWV, Watkins H, Enemuo CA, Hartwell BL, Chang JY, Xiao S, Rodrigues KA, Cirelli KM, Li N, Haupt S, Aung A, Cossette B, Abraham W, Kataria S, Bastidas R, Bhiman J, Linde C, Bloom NI, Groschel B, Georgeson E, Phelps N, Thomas A, Bals J, Carnathan DG, Lingwood D, Burton DR, Alter G, Padera TP, Belcher AM, Schief WR, Silvestri G, Ruprecht RM, Crotty S, Irvine DJ, A particulate saponin/TLR agonist vaccine adjuvant alters lymph flow and modulates adaptive immunity. Sci. Immunol 6, eabf1152 (2021). 34860581 10.1126/sciimmunol.abf1152 PMC8763571

[52] Briney BS, Willis JR, James E Crowe J, Human Peripheral Blood Antibodies with Long HCDR3s Are Established Primarily at Original Recombination Using a Limited Subset of Germline Genes. PLoS ONE 7, e36750 (2012). 22590602 10.1371/journal.pone.0036750 PMC3348910

[53] Willis JR, Berndsen ZT, Ma KM, Steichen JM, Schiffner T, Landais E, Liguori A, Kalyuzhniy O, Allen JD, Baboo S, Omorodion O, Diedrich JK, Hu X, Georgeson E, Phelps N, Eskandarzadeh S, Groschel B, Kubitz M, Adachi Y, Mullin T-M, Alavi NB, Falcone S, Himansu S, Carfi A, Wilson IA, Yates JR, Paulson JC, Crispin M, Ward AB, Schief WR, Human immunoglobulin repertoire analysis guides design of vaccine priming immunogens targeting HIV V2-apex broadly neutralizing antibody precursors. Immunity 55, 2149–2167.e9 (2022). 36179689 10.1016/j.immuni.2022.09.001 PMC9671094

[54] Mishra N, Liang B, Roark RS, Ghosh A, Callaghan S, Lee W-H, Li X, Vo AL, Avillion G, Chowdhury RR, Habib RH, Bibollet-Ruche F, Giese G, Oberoi P, Amereh K, Somanathan A, Zhu Y, Zhang Y, Kassab M, Tjio L, Andrabi S, Reyes RA, Allen JD, James NE, Randall KN, van der Maas L, Ben-Akiva E, Kacmarek-Michaels K, Plante S, Martella CL, Skelly AN, Singh A, Hurtado J, Dueker K, Capozzola T, Nedellec R, Ozorowski G, Lewis MM, Falcone S, Carfi A, Himansu S, Shapiro L, Crispin M, Hahn BH, Briney B, Irvine DJ, Burton DR, Ward AB, Batista FD, Kwong PD, Shaw GM, Raiees A, Germline-targeting HIV immunogen induces cross-neutralizing antibodies in outbred macaques. bioRxiv [Preprint] (2025). 10.1101/2025.10.22.684023. PMC13274938 41985438

[55] Melo M, Porter E, Zhang Y, Silva M, Li N, Dobosh B, Liguori A, Skog P, Landais E, Menis S, Sok D, Nemazee D, Schief WR, Weiss R, Irvine DJ, Immunogenicity of RNA Replicons Encoding HIV Env Immunogens Designed for Self-Assembly into Nanoparticles. Mol. Ther 27, 2080–2090 (2019). 31515132 10.1016/j.ymthe.2019.08.007 PMC6904793

[56] Montefiori DC, Roederer M, Morris L, Seaman MS, Neutralization tiers of HIV-1. Curr. Opin. HIV AIDS 13, 128–136 (2018). 29266013 10.1097/COH.0000000000000442 PMC5802254

[57] Wiehe K, Bradley T, Meyerhoff RR, Hart C, Williams WB, Easterhoff D, Faison WJ, Kepler TB, Saunders KO, Alam SM, Bonsignori M, Haynes BF, Functional Relevance of Improbable Antibody Mutations for HIV Broadly Neutralizing Antibody Development. Cell Host Microbe 23, 759–765.e6 (2018). 29861171 10.1016/j.chom.2018.04.018 PMC6002614

[58] Mishra N, Liang B, Roark R, Ghosh A, Callaghan S, Lee W, Li X, Vo A, Avillion G, Chowdhury R, Habib R, Bibollet-Ruche F, Giese G, Oberoi P, Amereh K, Somanathan A, Zhou Y, Zhang Y, Kassab M, Tijo L, Andrabi S, Reyes R, Allen J, James N, Randall K Jr, van der Maas L, Ben-Akiva E, Kacmarek-Michaels K, Plante S, Martella C, Skelly A, Singh A, Hurtado J, Dueker K, Capozzola T, Nedellec R, Ozorowski G, Lewis M, Falcone S, Carfi A, Himansu S, Shapiro L, Crispin M, Hahn B, Briney B, Irvine D, Burton D, Ward A, Batista F, Kwong P, Shaw G, Andrabi R, Germline-targeting HIV immunogen induces cross-neutralizing antibodies in outbred macaques. Immunity (2025). 10.1016/j.immuni.2026.03.012 PMC13274938 41985438

[59] Roark RS, Li H, Williams WB, Chug H, Mason RD, Gorman J, Wang S, Lee F-H, Rando J, Bonsignori M, Hwang K-K, Saunders KO, Wiehe K, Moody MA, Hraber PT, Wagh K, Giorgi EE, Russell RM, Bibollet-Ruche F, Liu W, Connell J, Smith AG, DeVoto J, Murphy AI, Smith J, Ding W, Zhao C, Chohan N, Okumura M, Rosario C, Ding Y, Lindemuth E, Bauer AM, Bar KJ, Ambrozak D, Chao CW, Chuang G-Y, Geng H, Lin BC, Louder MK, Nguyen R, Zhang B, Lewis MG, Raymond DD, Doria-Rose NA, Schramm CA, Douek DC, Roederer M, Kepler TB, Kelsoe G, Mascola JR, Kwong PD, Korber BT, Harrison SC, Haynes BF, Hahn BH, Shaw GM, Recapitulation of HIV-1 Env-antibody coevolution in macaques leading to neutralization breadth. Science 371, eabd2638 (2021). 33214287 10.1126/science.abd2638 PMC8040783

[60] McGuire AT, Gray MD, Dosenovic P, Gitlin AD, Freund NT, Petersen J, Correnti C, Johnsen W, Kegel R, Stuart AB, Glenn J, Seaman MS, Schief WR, Strong RK, Nussenzweig MC, Stamatatos L, Specifically modified Env immunogens activate B-cell precursors of broadly neutralizing HIV-1 antibodies in transgenic mice. Nat Commun 7, 10618 (2016). 26907590 10.1038/ncomms10618 PMC4770077

[61] Steichen JM, Lin YC, Havenar-Daughton C, Pecetta S, Ozorowski G, Willis JR, Toy L, Sok D, Liguori A, Kratochvil S, Torres JL, Kalyuzhniy O, Melzi E, Kulp DW, Raemisch S, Hu X, Bernard SM, Georgeson E, Phelps N, Adachi Y, Kubitz M, Landais E, Umotoy J, Robinson A, Briney B, Wilson IA, Burton DR, Ward AB, Crotty S, Batista FD, Schief WR, A generalized HIV vaccine design strategy for priming of broadly neutralizing antibody responses. Science 366 (2019). 10.1126/science.aax4380 PMC7092357 31672916

[62] Luo S, Jing C, Ye AY, Kratochvil S, Cottrell CA, Koo J-H, Chapdelaine Williams A, Francisco LV, Batra H, Lamperti E, Kalyuzhniy O, Zhang Y, Barbieri A, Manis JP, Haynes BF, Schief WR, Batista FD, Tian M, Alt FW, Humanized V(D)J-rearranging and TdT-expressing mouse vaccine models with physiological HIV-1 broadly neutralizing antibody precursors. Proc. Natl. Acad. Sci 120, e2217883120 (2023). 36574685 10.1073/pnas.2217883120 PMC9910454

[63] Sok D, Briney B, Jardine JG, Kulp DW, Menis S, Pauthner M, Wood A, Lee E-C, Le KM, Jones M, Ramos A, Kalyuzhniy O, Adachi Y, Kubitz M, MacPherson S, Bradley A, Friedrich GA, Schief WR, Burton DR, Priming HIV-1 broadly neutralizing antibody precursors in human Ig loci transgenic mice. Science 353, 1557–1560 (2016). 27608668 10.1126/science.aah3945 PMC5404394

[64] Abbott RK, Lee JH, Menis S, Skog P, Rossi M, Ota T, Kulp DW, Bhullar D, Kalyuzhniy O, Havenar-Daughton C, Schief WR, Nemazee D, Crotty S, Precursor Frequency and Affinity Determine B Cell Competitive Fitness in Germinal Centers, Tested with Germline-Targeting HIV Vaccine Immunogens. Immunity 48, 133–146.e6 (2018). 29287996 10.1016/j.immuni.2017.11.023 PMC5773359

[65] Graham BS, Gilman MSA, McLellan JS, Structure-Based Vaccine Antigen Design. Annu. Rev. Med 70, 91–104 (2019). 30691364 10.1146/annurev-med-121217-094234 PMC6936610

[66] Bhagchandani SH, Yang L, Lam JH, Maiorino L, Ben-Akiva E, Rodrigues KA, Romanov A, Suh H, Aung A, Wu S, Wadhera A, Chakraborty AK, Irvine DJ, Two-dose priming immunization amplifies humoral immunity by synchronizing vaccine delivery with the germinal center response. Sci. Immunol 9, eadl3755 (2024). 39303017 10.1126/sciimmunol.adl3755 PMC11492009

[67] Habib R, Solieva SO, Lin ZJ, Ghosh S, Bayruns K, Singh M, Agostino CJ, Tursi NJ, Sowers KJ, Huang J, Roark RS, Purwar M, Park Y, Ayyanathan K, Li H, Carey JW, Kim A, Park J, McCanna ME, Skelly AN, Chokkalingam N, Kriete S, Shupin N, Huynh A, Walker S, Laenger N, Du J, Cui J, Hahn BH, Patel A, Escolano A, Kwong PD, Shapiro L, Bowman GR, Shaw GM, Weiner DB, Pallesen J, Kulp DW, Deep Mining of the Human Antibody Repertoire Identifies Frequent and Immunogenetically Diverse CDRH3 Topologies Targetable by Vaccination. bioRxiv [Preprint] (2024). 10.1101/2024.10.04.616739. PMC13143044 42054357

[68] Nowosad CR, Spillane KM, Tolar P, Germinal center B cells recognize antigen through a specialized immune synapse architecture. Nat. Immunol 17, 870–877 (2016). 27183103 10.1038/ni.3458 PMC4943528

[69] Xie Z, Lin Y-C, Steichen JM, Ozorowski G, Kratochvil S, Ray R, Torres JL, Liguori A, Kalyuzhniy O, Wang X, Warner JE, Weldon SR, Dale GA, Kirsch KH, Nair U, Baboo S, Georgeson E, Adachi Y, Kubitz M, Jackson AM, Richey ST, Volk RM, Lee JH, Diedrich JK, Prum T, Falcone S, Himansu S, Carfi A, Yates JR, Paulson JC, Sok D, Ward AB, Schief WR, Batista FD, mRNA-LNP HIV-1 trimer boosters elicit precursors to broad neutralizing antibodies. Science 384, eadk0582 (2024). 38753770 10.1126/science.adk0582 PMC11488660

[70] Brochet X, Lefranc M-P, Giudicelli V, IMGT/V-QUEST: the highly customized and integrated system for IG and TR standardized V-J and V-D-J sequence analysis. Nucleic Acids Res. 36, W503–W508 (2008). 18503082 10.1093/nar/gkn316 PMC2447746

[71] Giudicelli V, Brochet X, Lefranc M-P, IMGT/V-QUEST: IMGT standardized analysis of the immunoglobulin (IG) and T cell receptor (TR) nucleotide sequences. Cold Spring Harb. Protoc 2011, 695–715 (2011). 21632778 10.1101/pdb.prot5633

[72] Giudicelli V, Duroux P, Rollin M, Aouinti S, Folch G, Jabado-Michaloud J, Lefranc M-P, Kossida S, IMGT^®^ Immunoinformatics Tools for Standardized V-DOMAIN Analysis. Methods Mol. Biol. Clifton NJ 2453, 477–531 (2022). 10.1007/978-1-0716-2115-8_24 PMC9761511 35622340

[73] Madeira F, Madhusoodanan N, Lee J, Eusebi A, Niewielska A, Tivey ARN, Lopez R, Butcher S, The EMBL-EBI Job Dispatcher sequence analysis tools framework in 2024. Nucleic Acids Res. 52, W521–W525 (2024). 38597606 10.1093/nar/gkae241 PMC11223882

[74] Stamatakis A, RAxML-VI-HPC: maximum likelihood-based phylogenetic analyses with thousands of taxa and mixed models. Bioinformatics 22, 2688–2690 (2006). 16928733 10.1093/bioinformatics/btl446

[75] Suloway C, Pulokas J, Fellmann D, Cheng A, Guerra F, Quispe J, Stagg S, Potter CS, Carragher B, Automated molecular microscopy: the new Leginon system. J Struct Biol 151, 41–60 (2005). 15890530 10.1016/j.jsb.2005.03.010

[76] Punjani A, Rubinstein JL, Fleet DJ, Brubaker MA, cryoSPARC: algorithms for rapid unsupervised cryo-EM structure determination. Nat. Methods 14, 290–296 (2017). 28165473 10.1038/nmeth.4169

[77] Pettersen EF, Goddard TD, Huang CC, Meng EC, Couch GS, Croll TI, Morris JH, Ferrin TE, UCSF ChimeraX: Structure visualization for researchers, educators, and developers. Protein Sci 30, 70–82 (2021). 32881101 10.1002/pro.3943 PMC7737788

[78] Emsley P, Cowtan K, Coot: model-building tools for molecular graphics. Acta Crystallogr. D Biol. Crystallogr 60, 2126–2132 (2004). 15572765 10.1107/S0907444904019158

[79] Adams PD, Gopal K, Grosse-Kunstleve RW, Hung L-W, Ioerger TR, McCoy AJ, Moriarty NW, Pai RK, Read RJ, Romo TD, Sacchettini JC, Sauter NK, Storoni LC, Terwilliger TC, Recent developments in the PHENIX software for automated crystallographic structure determination. J. Synchrotron Radiat 11, 53–55 (2004). 14646133 10.1107/s0909049503024130

[80] Davis IW, Murray LW, Richardson JS, Richardson DC, MOLPROBITY: structure validation and all-atom contact analysis for nucleic acids and their complexes. Nucleic Acids Res. 32, W615–619 (2004). 15215462 10.1093/nar/gkh398 PMC441536

[81] Barad BA, Echols N, Wang RY-R, Cheng Y, DiMaio F, Adams PD, Fraser JS, EMRinger: side chain-directed model and map validation for 3D cryo-electron microscopy. Nat. Methods 12, 943–946 (2015). 26280328 10.1038/nmeth.3541 PMC4589481

[82] Hraber P, Korber B, Wagh K, Montefiori D, Roederer M, A single, continuous metric to define tiered serum neutralization potency against HIV. eLife 7, e31805 (2018). 29350181 10.7554/eLife.31805 PMC5788501

