# Supplementary material for: Rapidly acquired HIV-1 neutralization breadth in a macaquized V2 apex mouse model after a single bolus immunization
Source: Sci Immunol. Author manuscript; Available in PMC 2026 Apr 23. (PMC13104692; doi:10.1126/sciimmunol.adz5064)
Supplement: Supplemental Material — Supplementary Figure 1. Further characterization of Q23-SCT immunogens, related to Figure 1. Supplementary Figure 2. Construct sequences, related to Figure 1. Supplementary Figure 3. Further characterization of Q23-SCT27 antigenicity and expression, related to Figure 1. Supplementary Figure 4. Immunization with Q23-SCT after higher frequency of adoptive transfer leads to recruitment and activation of V033a-UCA I1 B cells, related to Figure 2. Supplementary Figure 5. Sorting strategy of Ag+ V033a UCA I1 B cells post immunization. After gating on lymphocytes and gating out possible doublets, related to figures 3, 6, and 7. Supplementary Figure 6. Macaque antibody features, related to Figure 4. Supplementary Figure 7. Extended SHM analysis in immunized murine models, related to Figure 3. Supplementary Figure 8. Affinity and neutralization breadth of week 4 prime derived antibodies, related to Figure 4. Supplementary Figure 9. Q23-APEX-GT1 primed V033a-UCA I1 B cells can bind N187S escape variant, related to Figures 4 and 7. Supplementary Figure 10. Priming leads to minimal serum neutralization, related to Figures 4 and 7. Supplementary Figure 11. Select comparative structural features of V033a-UCA I1 and prime-derived antibody T6_P_H03 Supplementary Figure 12. Heterologous boost leads to expanded serum neutralization and LC usage in homologous vs N187S boost, related to Figures 6 and 7. Supplementary Figure 13. Neutralization of Q23 and N187S boost-derived antibodies, related to Figures 6, 7, and 8. Supplementary Figure 14. Select comparative structural features of V033-a lineage variants, related to Figures 5 and 8. Supplementary Figure 15. Single-particle cryo-EM validation for murine V033-a antibodies in complex with HIV envelope, related to Figure 5. Supplementary Table 1. Post-boost mutation frequencies. Supplementary Table 2. Cryo-EM statistics. Supplementary Table 3. Reagents used. [file NIHMS2148533-supplement-Supplemental_Material.pdf]

Science
AAAS

# Supplementary Materials for

**Rapidly acquired HIV-1 neutralization breadth in a macaquized V2 apex mouse model after a single bolus immunization**

Amrit Raj Ghosh, Rumi Habib, Nitesh Mishra, Ryan S. Roark, Madhav Akauliya, Ali A. Albowaidey, Joel D. Allen, Khaled Amereh, Gabriel Avillion, Maria Bottermann, Bo Liang, Namit Chaudhary, Sean Callaghan, Jonathan Dye, Xuduo Li, Jordan R. Ellis-Pugh, Rohan Roy Chowdhury, Nicole E. James, Xiaotie Liu, Laura Maiorino, Paula M. Villavicencio, Rebecca Nedellec, Prabhgun Oberoi, Kirsten J. Sowers, Younghoon Park, Thavaleak Prum, Linette Rodriguez, Maria Ssozi, Jonathan L. Torres, Agnes A. Walsh, John E. Warner, Stephanie R. Weldon, Liling Xu, Kevin Wiehe, Max Crispin, Andrew B. Ward, Usha Nair, Beatrice H. Hahn, Dennis R. Burton, Lawrence Shapiro, Peter D. Kwong, Darrell J. Irvine, Raiees Andrabi, George M. Shaw, Facundo D. Batista

*Corresponding Authors (R.A. raiees.andrabi@pennmedicine.upenn.edu; G.M.S. shawg@pennmedicine.upenn.edu; F.D.B. fbatista1@mgh.harvard.edu)

**The PDF file includes:**

Figs. S1 to S15

Tables S1 to S3

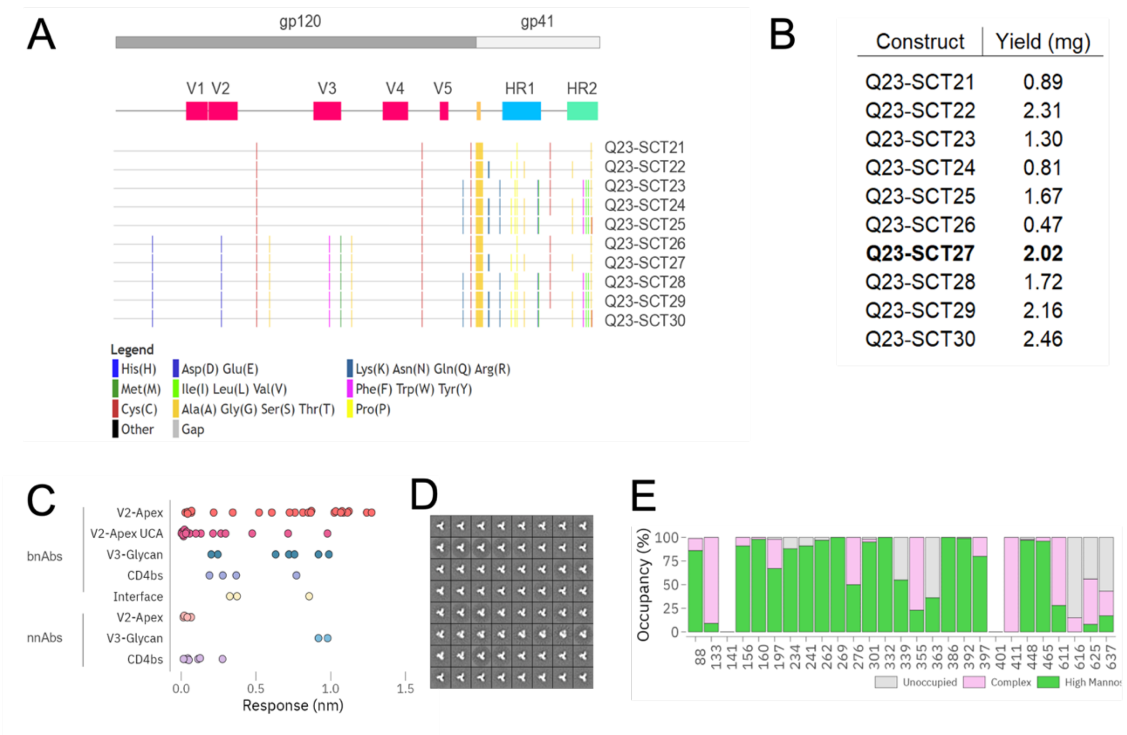


**Supplementary Figure 1. Further characterization of Q23-SCT immunogens, related to Figure 1.**

(**A**) Highlighter plots showing the mutations across all 10 SCTs compared to wildtype Q23 sequence. First box shows the placement of gp120 (colored dark gray) and gp41 (colored light gray) while the second box shows the placement of variable loops and the refolding regions of HR1-2 (Heptad Repeat 1-2). (G4S)2 linker is shown in yellow. Mutations are marked by colored vertical lines.

(**B**) Yield (in mg) of GNL (*Galanthus nivalis* Lectin)-purified proteins prior to SEC (Size Exclusion Chromatography) purification from 100 ml of 293F. Q23-SCT27 is highlighted in bold.

(**C**) BLI (Biolayer Interferometry) binding responses of a large panel of 70 mAbs against all epitope class of HIV-1 Env trimer against Q23-SCT27. mAbs are segregated in two bins of bnAbs and non-nAbs (nnAbs).

(**D**) Negatively stained EM of Q23-SCT27 trimers with 2D-averaged classes.

(**E**) Proteomics-based site-specific glycan analysis (SSGA) of Q23-SCT27. High mannose, green; complex glycan, pink; unoccupied, gray. No signal could be resolved at PNGS 141 and 401.

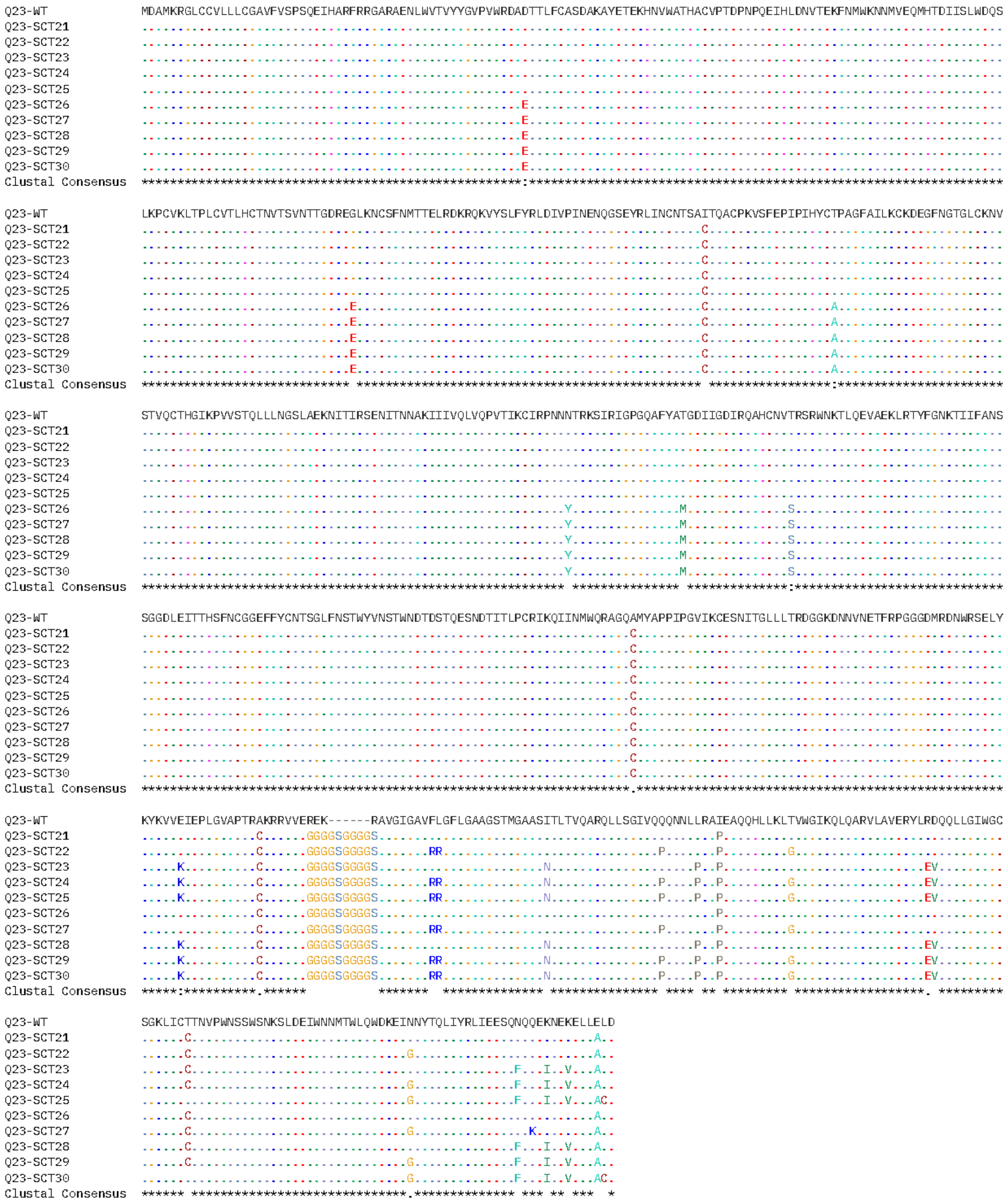

**Supplementary Figure 2. Construct sequences, related to Figure 1.**

Alignment of all designed constructs with individual mutations relative to wildtype Q23 envelope glycoprotein sequence. Mutations are colored according to side-chain chemistry. Unchanged positions are shown by dots.

A

| | Class | mAb | IC50 | BLI Response | Surface |
|---|---|---|---|---|---|
| UCA | V2-Apex UCA | 42056-a.UCA | 10 | 0.01 | 931 |
| | | 40591-a.UCA | 10 | 0.02 | 1541 |
| | | RHA1 UCA | 10 | 0.21 | 3272 |
| | | 6561-a.UCA | 10 | 0.00 | ND |
| | | 6070-a.UCA | 10 | 0.27 | 1356 |
| | | T646-a.UCA | 10 | 0.10 | 961 |
| | | 41328-a.UCA | 10 | 0.47 | 6844 |
| | | V033-a.UCA | 10 | 0.03 | 2000 |
| | | V031-a.UCA | 10 | 0.71 | 6583 |
| | | 42056-b.UCA | 10 | 0.02 | 1390 |
| | | 5695-b.UCA | 10 | 0.04 | 1139 |
| | | PCT64 LMCA | 10 | 0.30 | 5512 |
| | | CH01 iGL | 0.477 | 0.13 | 4929 |
| | | CAP256-VRC26 UCA | 10 | 0.02 | 921 |
| | | PG9 iGL | 10 | 0.02 | 3176 |
| bnAbs | V2-Apex | 42056-a.01 | 0.00957 | 1.12 | 7482 |
| | | 40591-a.01 | 0.02247 | 0.87 | 10825 |
| | | RHA1.01 | 0.0046 | 1.02 | 11859 |
| | | T646-a.01 | 0.0046 | 1.27 | 8932 |
| | | 41328-a.01 | 0.0046 | 1.09 | 10689 |
| | | V033-a.01 | 0.00504 | 1.07 | 4250 |
| | | V031-a.01 | 0.0046 | 1.08 | 2151 |
| | | 42056-b.01 | 10 | 0.03 | 995 |
| | | PG9 | 0.0046 | 1.11 | 11686 |
| | | PG16 | 0.0046 | 0.98 | 11965 |
| | | PGT145 | 1.101 | 1.24 | 9236 |
| | | PGDM1400 | 0.0046 | 0.81 | 11256 |
| | | PCT64.35S | 0.0046 | 0.61 | 8007 |
| | | CH01 | 0.00769 | 0.07 | 8877 |
| | | CAP256.09 | 0.04755 | 0.52 | 8521 |
| | | CAP256.25 | 0.0046 | 0.76 | 9382 |
| | V3 Glycan | PGT121 | 0.0122 | 0.76 | 22496 |
| | | PGT128 | 0.00578 | 0.99 | 26611 |
| | | PGT130 | 0.05691 | 0.25 | 8821 |
| | | 10-1074 | 0.0046 | 0.92 | 22536 |
| | | PGT135 | 0.1166 | 0.20 | 14508 |
| | | 2G12 | 10 | 0.72 | 18989 |
| | CD4bs | VRC01 | 0.1242 | 0.37 | 13147 |
| | | VRC06 | 0.3822 | 0.28 | 15505 |
| | | HJ16 | 10 | 0.02 | 1460 |
| | | CH103 | 3.973 | 0.19 | 4684 |
| | | 3BNC60 | 0.01939 | 0.77 | 16274 |
| | gp120-41 | PGT151 | 0.0046 | 0.33 | 6637 |
| | | 35022 | 0.0046 | 0.37 | 11831 |
| | | VRC34.01 | 0.2124 | 0.86 | 2215 |
| nnAbs | V2i | 2909 | 6.015 | 0.07 | 1033 |
| | | 830A | 10 | 0.04 | 1398 |
| | | 697 | 10 | 0.01 | 1300 |
| | V3 nnAb | 2557 | 10 | 0.92 | 2464 |
| | | 3074 | 10 | 0.98 | 3041 |
| | CD4bs nnAb | b6 | 10 | 0.28 | 2785 |
| | | b12 | 10 | 0.11 | 1069 |
| | | F105 | 10 | 0.13 | 2340 |
| | CD4i | A32 | 10 | 0.04 | 1219 |
| | | 17b | 10 | 0.03 | 9020 |
| | | E51 | 10 | 0.05 | 974 |

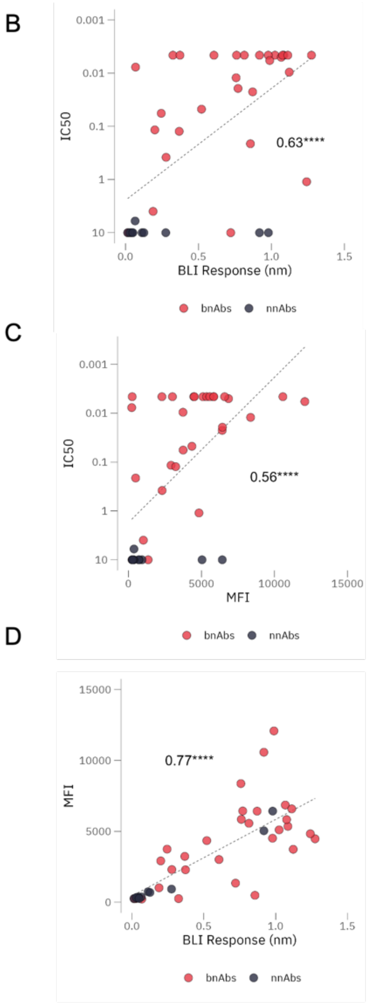


E

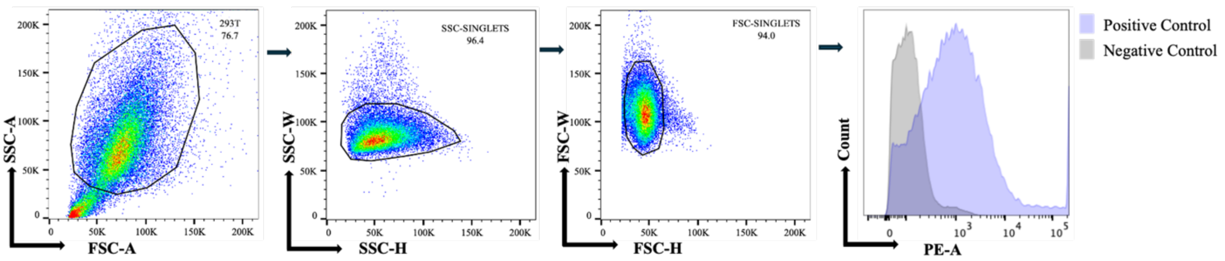


**Supplementary Figure 3. Further characterization of Q23-SCT27 antigenicity and expression, related to Figure 1.**

(**A**) Extended antigenicity data for Q23-SCT27 against a larger panel of bnAbs, nnAbs and V2 apex UCAs (human and rhesus). Neutralization was tested starting at a concentration of 10 µg/ml and $IC_{50}$ value of 10 denotes 50% neutralization was not reached at maximum concentration tested. For BLI, Q23-SCT27 trimer was tested at 500 nm and antibodies were tested at 10 µg/ml. For cell surface binding, Q23-SCT27 was appended to the wild-type transmembrane (TM) from Q23 and tested for binding using flow cytometry.

(**B–C**) Correlation plots for neutralization (IC50) versus soluble (BLI) and cell surface expression (MFI), and (**D**) Soluble (BLI) versus cell surface expression (MFI) show significant positive correlation (Pearson's coefficient > 0.5). ****$P$< 0.0001. **(E)** Representative flow cytometry plots showing the sequential gating strategy used for analysis. Total events were gated based on forward scatter area (FSC-A) versus side scatter area (SSC-A) to exclude debris and select the main cell population. Singlets were identified using SSC-H versus SSC-W to exclude aggregates and doublets. Further singlet refinement was performed using FSC-H versus FSC-W. Histogram showing event counts versus phycoerythrin (PE) area for cells stained with the positive control antibody PGT145 and negative control antibody CC40.8 specific for SARS-CoV-2. Mean fluorescence intensity (MFI) was calculated for each sample from the final gated population.

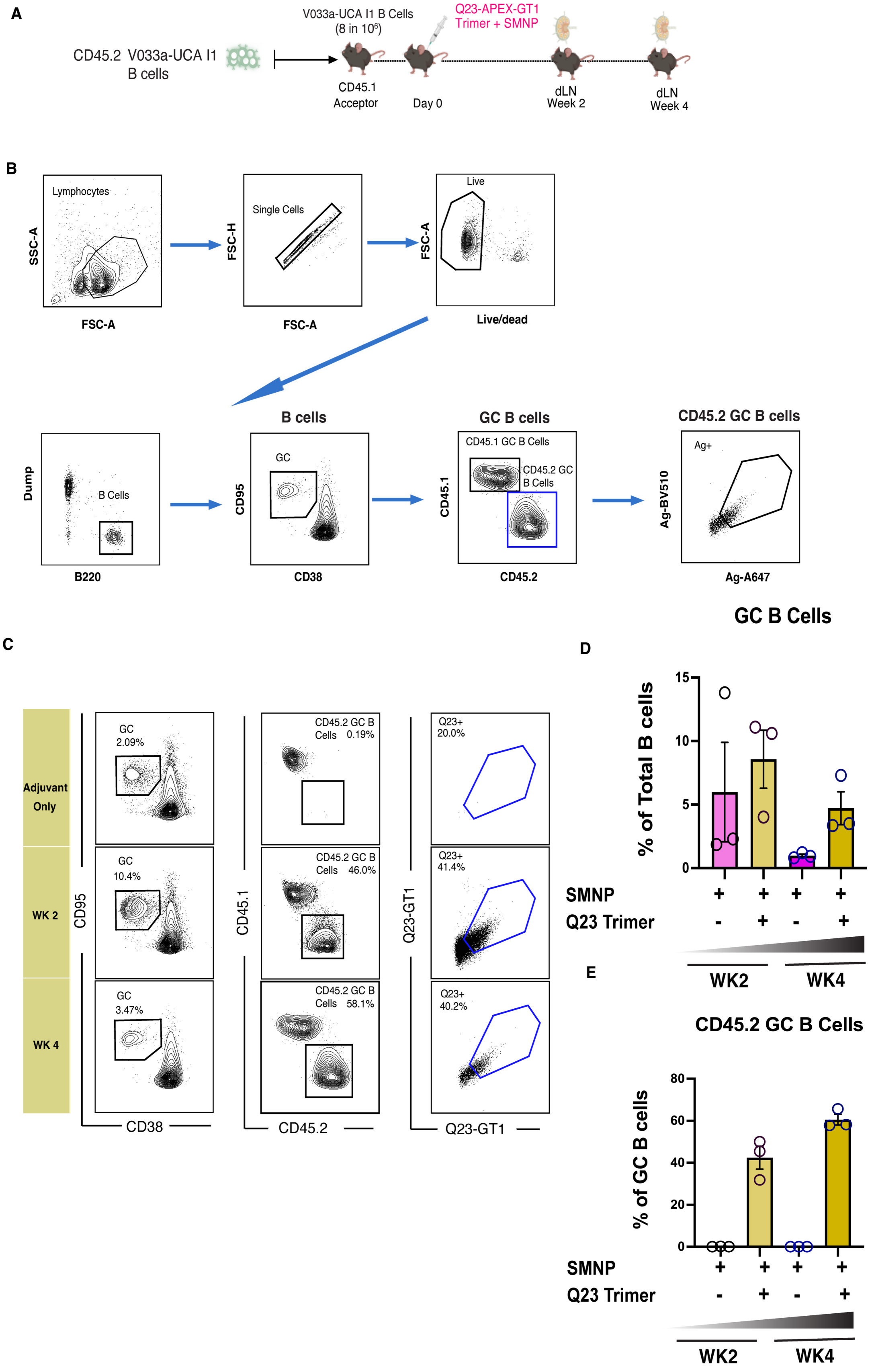

A
CD45.2 V033a-UCA I1
B cells
V033a-UCA I1 B Cells
(8 in 10⁶)
Q23-APEX-GT1
Trimer + SMNP
CD45.1
Acceptor
Day 0
dLN
Week 2
dLN
Week 4
B
Lymphocytes
SSC-A
FSC-A
Single Cells
FSC-H
FSC-A
Live
FSC-A
Live/dead
B cells
GC B cells
CD45.2 GC B cells
Dump
B Cells
B220
GC
CD95
CD38
CD45.1 GC B Cells
CD45.2 GC
B Cells
CD45.1
CD45.2
Ag+
Ag-BV510
Ag-A647
C
Adjuvant
Only
WK 2
WK 4
GC
2.09%
GC
10.4%
GC
3.47%
CD95
CD38
CD45.2 GC B
Cells 0.19%
CD45.2 GC B
Cells 46.0%
CD45.2 GC B
Cells 58.1%
CD45.1
CD45.2
Q23+
20.0%
Q23+
41.4%
Q23+
40.2%
Q23-GT1
Q23-GT1
D
GC B Cells
% of Total B cells
SMNP
Q23 Trimer
WK2
WK4
E
CD45.2 GC B Cells
% of GC B cells
SMNP
Q23 Trimer
WK2
WK4

**Supplementary Figure 4. Immunization with Q23-SCT after higher frequency of adoptive transfer leads to recruitment and activation of V033a-UCA I1 B cells, related to Figure 2.**

(**A**) Schematic of mouse adoptive transfer and immunization experiments. Mice received V033a-UCA I1 B cells through intravenous transfer one day prior to immunization with Q23-APEX-GT1 trimer adjuvanted with SMNP immunization. SMNP-only immunization served as a control (n=3 mice per group, one experiment).

(B) Gating strategy for flow cytometry analysis of single cell suspensions from draining lymph nodes.

(**C**) Representative FACS plots showing germinal centers, CD45.2 B cells in GCs and their binding to Q23-APEX-GT1 during weeks 2 and 4 post-immunization with Q23-APEX-GT1 trimer with SMNP.

(**D,E**) Quantification of (D) GC B cells and (E) CD45.2 B cells in GCs weeks 2 and 4 post-immunization.

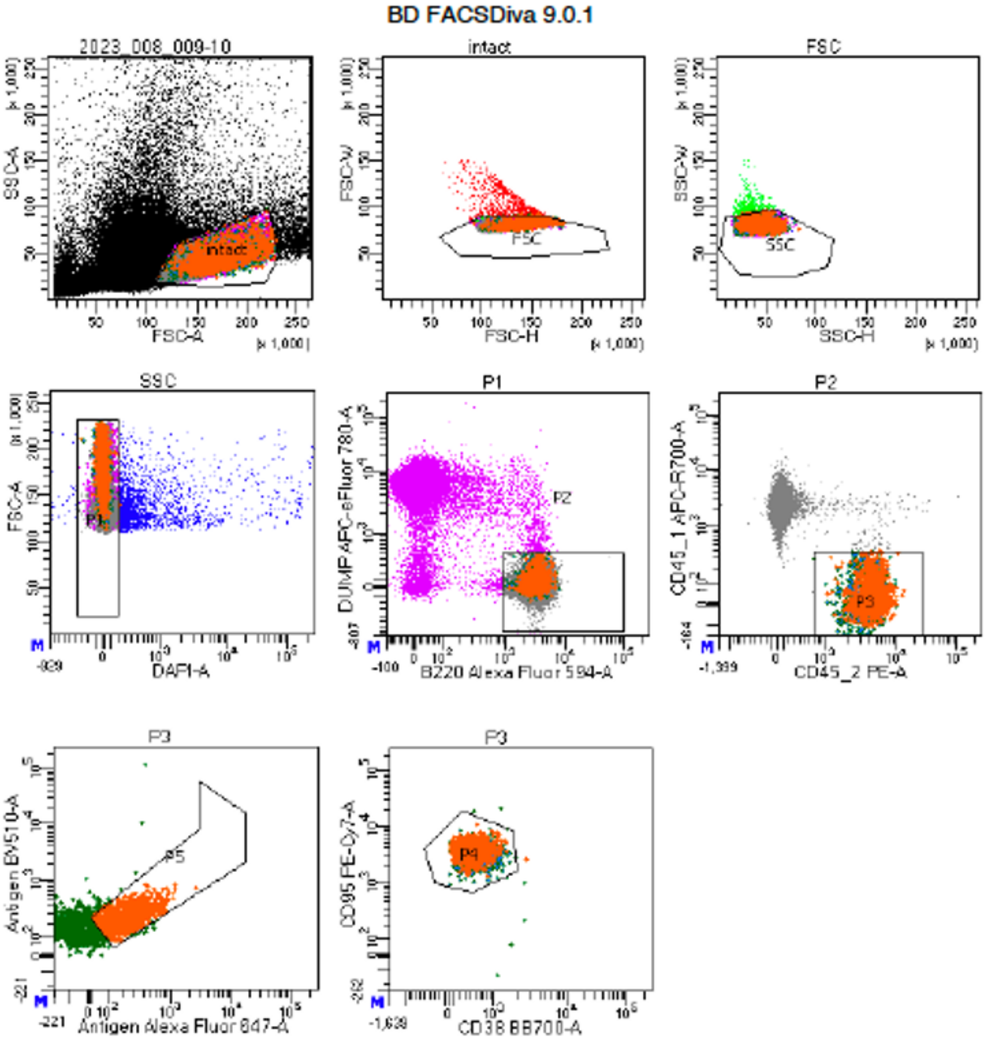


Tube: 10

| Population | #Events | %Parent | %Total |
|---|---|---|---|
| All Events | 310,768 | #### | 100.0 |
| intact | 185,525 | 59.7 | 59.7 |
| FSC | 184,781 | 99.6 | 59.5 |
| SSC | 184,347 | 99.8 | 59.3 |
| P1 | 182,308 | 98.9 | 58.7 |
| P2 | 60,454 | 33.2 | 19.5 |
| P3 | 2,028 | 3.4 | 0.7 |
| P4 | 2,020 | 99.6 | 0.7 |
| P5 | 1,048 | 51.7 | 0.3 |

**Supplementary Figure 5. Sorting strategy of Ag$^{+}$ V033a UCA I1 B cells post immunization. After gating on lymphocytes and gating out possible doublets, related to figures 3, 6, and 7.**

Live B cells were checked for antigen positivity and were sorted. Most of the antigen positive B

cells in the gate P5 were from germinal centers (CD38 $^{lo}$ CD95 $^{hi}$) as can be seen through abundance of P5 (orange) cells in the P4 gate. P5 cells were sorted into tubes.

**A**

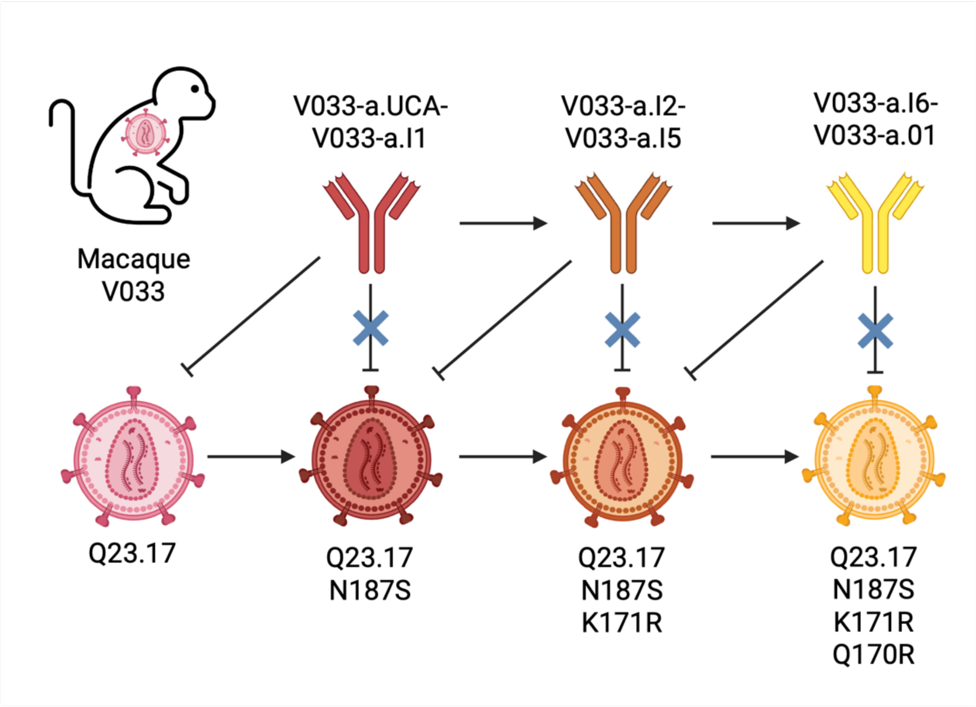


**B**

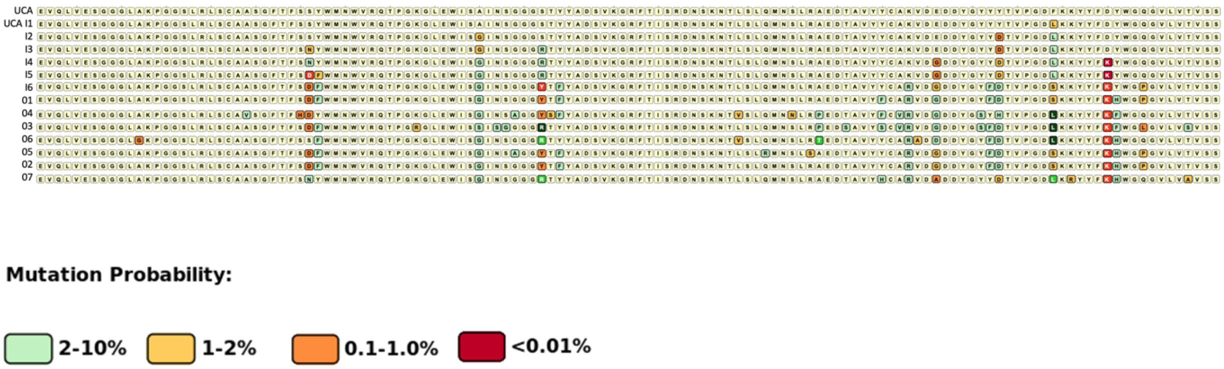


**Supplementary Figure 6. Macaque antibody features, related to Figure 4.**

(**A**) Schematic of SHIV infection and sequence isolation timeline. SHIV infected macaques were tracked and sampled for development of neutralizing antibodies. Concomitant escape mutations were also tracked (*22*, 59) .

(**B**) Probability of rare mutations as predicted by ARMaDILLO in mature macaque antibodies.

A

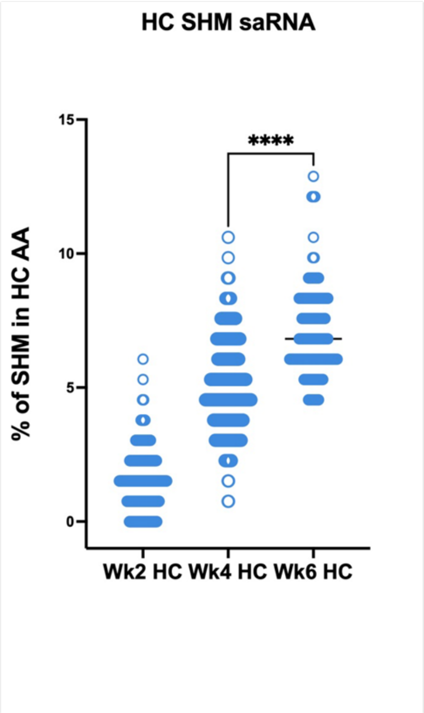


B

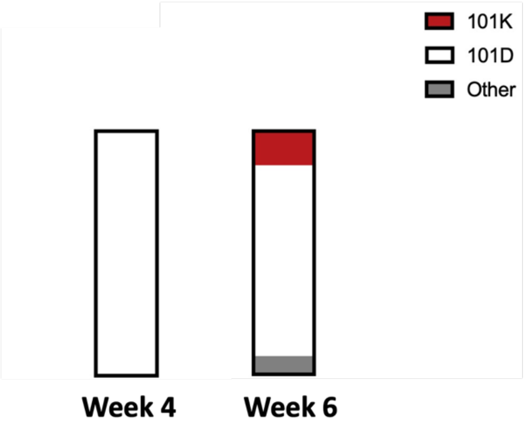


C

| Position | Mutation | Mutation probablity by ARMADiLLO | Frequency (%) at week 6 |
|---|---|---|---|
| HCDR1 | S31N | 1-2% | 100 |
| HCDR2 | S56Y | 0.1-1% | 5.65 |
| HCDR2 | T57R | 1-2% | 38.71 |
| HCDR3 | E97D | 2-10% | 24.19 |
| HCDR3 | $Y100_CF$ | 2-10% | 33.06 |
| HCDR3 | $Y100_DD$ | 2-10% | 44.35 |
| HCDR3 | D101K | <0.01% | 12.90 |

**Supplementary Figure 7. Extended SHM analysis in immunized murine models, related to Figure 3.**

(**A**) Total amino acid (AA) mutations in V033a-UCA I1 IGHV at weeks 2, 4, 6 post Q23-APEX-GT1 saRNA LNP immunization of mice adoptively transferred with V033a-UCA I1 KI B cells.

(**B**) Presence of rare lysine mutation in HCDR3 post priming immunization with Q23-APEX-GT1.

(**C**) Frequency of selected mutations in V033a-UCA I1 after single priming immunization at week 6.

A

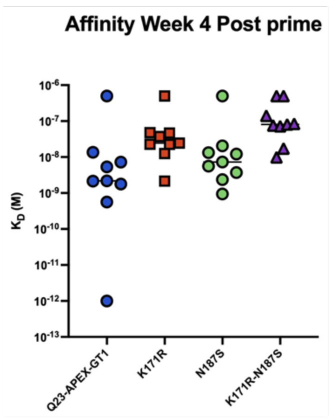


B

| | mAb1 | mAb2 | mAb3 | mAb4 | mAb5 | mAb6 | mAb7 | mAb8 | mAb9 | V033-a.01 |
|---|---|---|---|---|---|---|---|---|---|---|
| 246F3 | >50 | >50 | >50 | 15.01 | >50 | >50 | >50 | >50 | >50 | 0.03 |
| 25710 | 19.09 | 34.17 | >50 | 1.43 | >50 | 16.87 | 21.45 | >50 | >50 | 0.03 |
| BJOX0020000 | 5.80 | 4.82 | 11.88 | 0.66 | 17.08 | 5.48 | 7.17 | >50 | >50 | 0.04 |
| BG505.T332N | 3.59 | 4.36 | 6.96 | 0.77 | 41.10 | 2.14 | 2.63 | 49.31 | >50 | 0.02 |
| BG505.T332N.N160K | 6.53 | 6.67 | 10.12 | 1.20 | >50 | 2.96 | 3.42 | 41.24 | >50 | 0.32 |
| WITO.33 | >50 | >50 | >50 | 18.95 | >50 | 11.48 | 49.56 | >50 | >50 | 0.01 |
| Q23.17 | 0.14 | 0.06 | 0.21 | 0.03 | 0.56 | 0.05 | 0.10 | 0.77 | 5.24 | 0.02 |
| Q23.17 N160K | 2.37 | 1.16 | 24.85 | 0.27 | 4.12 | 0.40 | 0.70 | 3.80 | >50 | 0.00 |
| Q23.17 N187S | 1.65 | 1.41 | 5.09 | 0.27 | 26.07 | 1.27 | 1.32 | >50 | 36.54 | 0.12 |

C

| | | mAb2 | mAb4 | mAb7 | V033-a.01 |
|---|---|---|---|---|---|
| Global panel | 246F3 | >50 | 15 | >50 | 0.034 |
| | X1632 | >50 | >50 | >50 | >50 |
| | 25710 | 34 | 1.4 | 21 | 0.029 |
| | CNE55 | >50 | >50 | >50 | >50 |
| | X2278 | >50 | >50 | >50 | 0.73 |
| | BJOX0020000 | 4.8 | 0.66 | 7.2 | 0.040 |
| | CNE8 | >50 | >50 | >50 | >50 |
| | CE0217 | >50 | >50 | >50 | 0.014 |
| | TRO.11 | >50 | >50 | >50 | >50 |
| | CE1176.A3 | >50 | >50 | >50 | >50 |
| | CH119.10 | >50 | 47 | >50 | 0.11 |
| | BG505.T332N | 4.4 | 0.77 | 2.6 | 0.020 |
| | BG505.T332N.N160K | 6.7 | 1.2 | 3.4 | 0.32 |
| | CAP256SU | >50 | >50 | >50 | >50 |
| | ZM233.6 | >50 | >50 | >50 | >50 |
| | WITO.33 | >50 | 19 | 50 | 0.006 |
| | T250-4 | >50 | >50 | >50 | 0.23 |
| | T250-4 N160K | >50 | >50 | >50 | >50 |
| | Q23.17 | 0.062 | 0.029 | 0.096 | 0.017 |
| | Q23.17 N160K | 1.2 | 0.27 | 0.70 | 0.002 |
| | Q23.17 N187S | 1.4 | 0.27 | 1.3 | 0.12 |
| | CH505 TF | >50 | >50 | >50 | >50 |
| | MT145K (SIVcpz) | >50 | >50 | >50 | 0.041 |
| | CAM13K (SIVcpz) | >50 | >50 | 30 | 0.045 |
| | C1080 | 0.25 | 0.072 | 0.23 | 0.006 |
| Hraber Panel | 9004SS_A3_4 | >50 | >50 | >50 | >50 |
| | Q842.D12 | 18 | 0.82 | 8.5 | 0.027 |
| | 249M_B10 | 44 | 4.8 | 43 | >50 |
| | 7060101641A7(REV-) | >50 | >50 | >50 | >50 |
| | HIV-001428-2.42 | 0.50 | 0.24 | 0.53 | 0.049 |
| | HIV-16055-2.3 | 3.4 | 0.57 | 3.5 | 0.080 |
| | HIV-25925-2.22 | >50 | 21 | >50 | 4.6 |
| | 3728.v2.c6 | >50 | >50 | >50 | >50 |
| | RPW-0510.2 | >50 | >50 | >50 | >50 |
| | H029.12 | >50 | >50 | >50 | >50 |
| | SPK-0525.13 | >50 | 18 | 15 | 0.11 |

**Supplementary Figure 8. Affinity and neutralization breadth of week 4 prime derived antibodies, related to Figure 4.**

(**A**) Biolayer interferometry (BLI) affinity values of antibodies derived from week 4 post-saRNA or trimer prime against the autologous Q23.17 Env and escape variant envelopes.

(**B**) Neutralization breadth and potency of selected antibodies from week 4 post-prime against the autologous Q23.17 Env and escape variant envelopes (Q23.N187S, Q23.K171R.N187S). The mature bNAb from RM V033 is included for comparison (V033-a.01). Antibodies from Trimer+SMNP immunization are labelled in magenta and mAbs from Q23-GT1 saRNA LNP are

labelled in blue.

(**C**) $IC_{50}$ of three selected antibodies against a 37-member panel of HIV-1 strains including 11 from the Tier-2 Global panel and 11 from the Hraber panel (*82*).

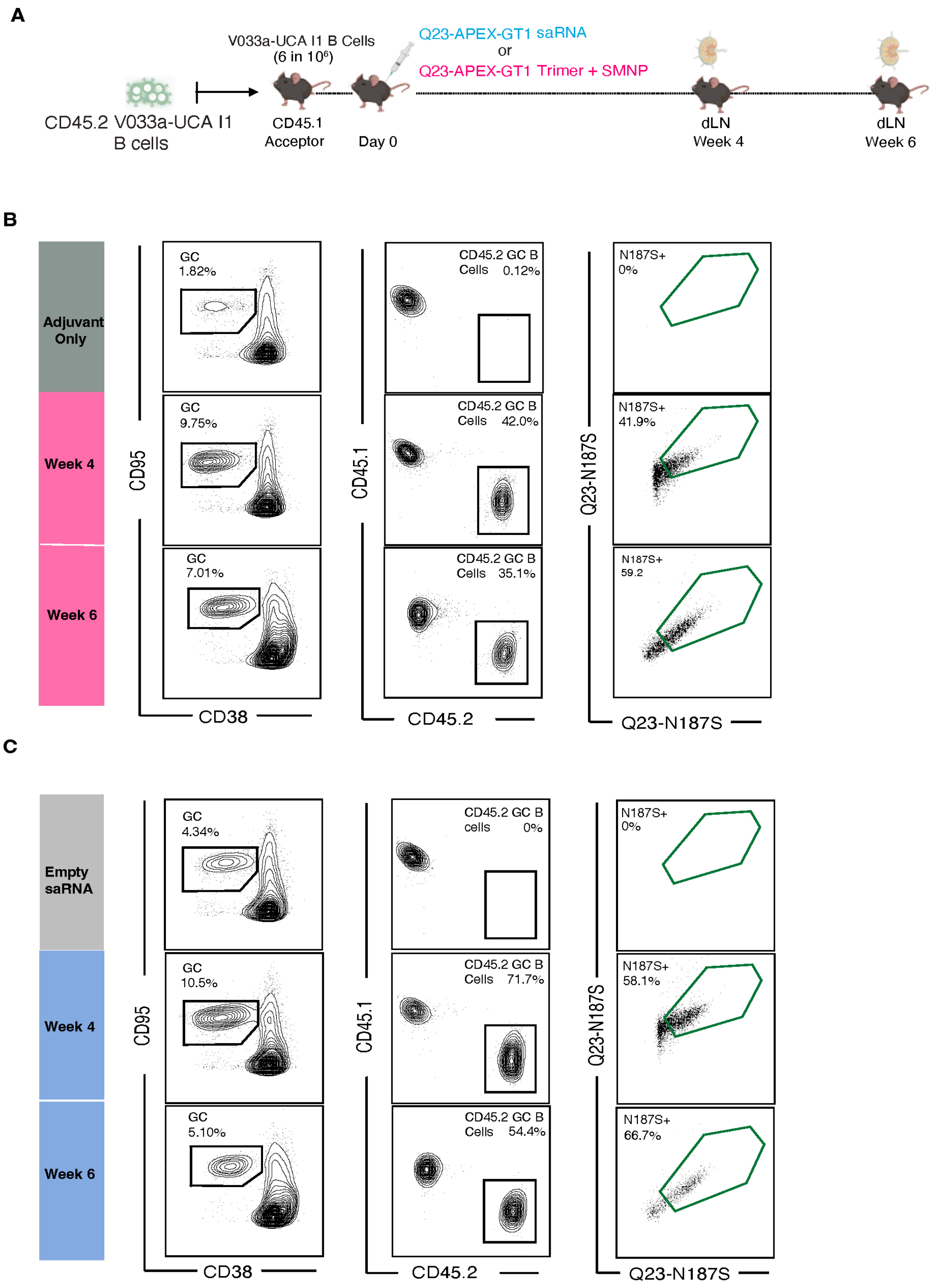


**Supplementary Figure 9. Q23-APEX-GT1 primed V033a-UCA I1 B cells can bind N187S escape variant, related to Figures 4 and 7.**

(**A**) Schematic presentation of mouse adoptive transfer and immunization experiments. SMNP

adjuvant without trimer is used as control for protein immunizations and LNPs containing an unrelated saRNA was used as control for Q23-APEX-GT1 saRNA LNP immunization part of the lymph nodes were used to assess N187S binding (n=3 mice per group, one experiment).

(**B, C**) Representative FACS plots showing germinal centers, CD45.2 B cells in GCs and their binding to the N187S escape variant during weeks 4 and 6 post immunization with (B) Q23-APEX-GT1 trimer with SMNP or (C) LNPs containing Q23-APEX-GT1 saRNA.

A

| | Empty Replicon | | | Q23 SCT Replicon | | | SMNP | | | Q23 SCT Protein | | |
|---|---|---|---|---|---|---|---|---|---|---|---|---|
| MULV | <100 | <100 | <100 | <100 | <100 | <100 | <100 | <100 | <100 | <100 | <100 | <100 |
| Q23.17 WT | <100 | <100 | <100 | <100 | <100 | <100 | <100 | <100 | <100 | 238 | 151 | 465 |
| Q23.17 N160K | <100 | <100 | <100 | <100 | <100 | <100 | <100 | <100 | <100 | <100 | <100 | <100 |

mAb IC50 (ug/mL)

| <0.1 | 0.1-1.0 | 1.0-10 | 10-50 |
|---|---|---|---|

B

| | Empty Replicon WK6 | | | Q23 Replicon WK6 | | | SMNP Only WK6 | | | Q23 Protein + SMNP WK6 | |
|---|---|---|---|---|---|---|---|---|---|---|---|
| | 2R | 1L1R | | 2R | 1L1R | | 2R | 1L1R | | 2R | 1L1R |
| Q23.17 WT | <100 | <100 | | <100 | <100 | | <100 | <100 | | 109 | 115 |
| Q23.17 N160K | <100 | <100 | | <100 | <100 | | <100 | <100 | | <100 | <100 |

**Supplementary Figure 10. Priming leads to minimal serum neutralization, related to Figures 4 and 7.**

(**A–B**) Reciprocal serum $ID_{50}$ values of animals receiving Q23-APEX-GT1 Prime at (A) week 4 3 mice per group and (B) week 6 2 mice per group post-immunization against Q23.17, Q23.17 N160K.

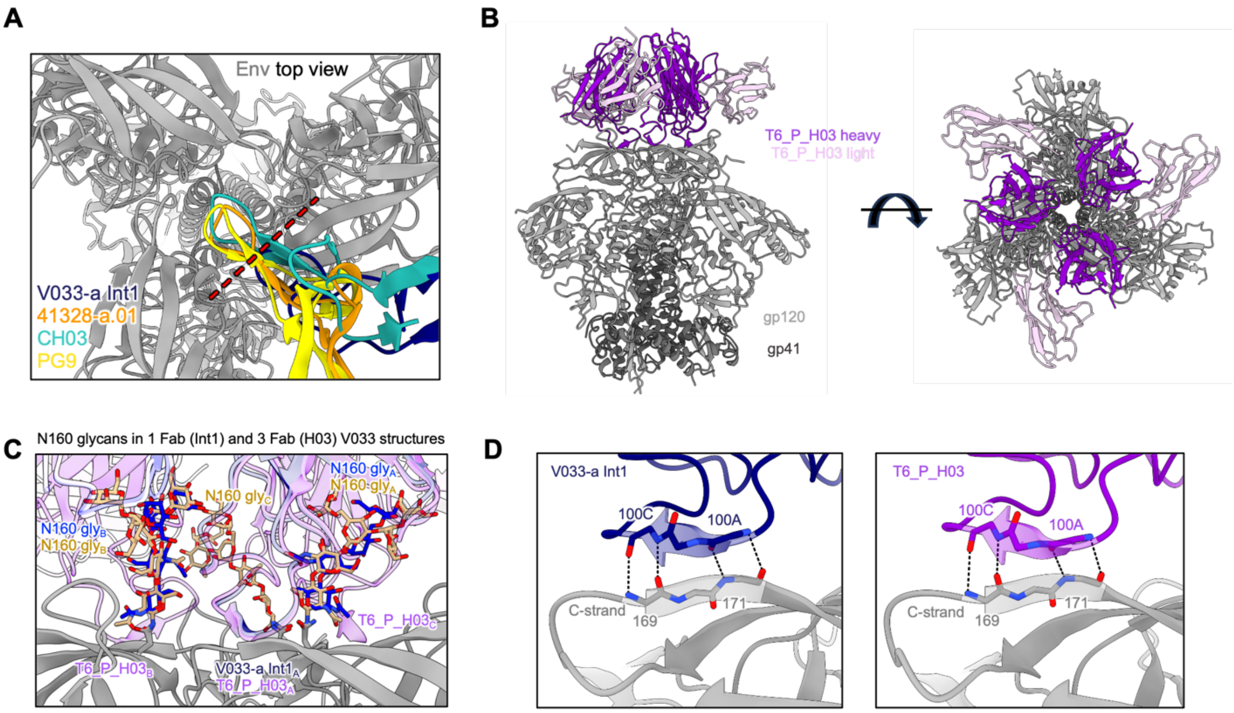


**Supplementary Figure 11. Select comparative structural features of V033a-UCA I1 and prime-derived antibody T6_P_H03**

(**A**) Top envelope trimer view for the gp120 alignment of V033-a I1 (PDB-9OMG) and axe-like V2 apex bnAbs 41328-a.01 (PDB-9BTL), CH03 (PDB-5ESZ), and PG9 (PDB-7T77). Only the Fab HCDR3 from each respective complex structure is shown for clarity. The red dashed line denotes the edge of the V033a UCA I1 HCDR3 tip to highlight the contrast in its relative positioning compared to other axe-like lineages, the latter of which all intersect with the trimer 3-fold axis.

(**B**) Orthogonal views for the cryo-EM structure of T6_P_H03 in complex with Q23-APEX-GT1 envelope trimer. The top view (right) reveals the middle of the trimer to be unencumbered by Fab HCDR3, similar to V033a-UCA I1, thereby allowing 3 Fabs to bind at the V2 apex.

(**C**) Comparing the conformation of N160 glycans recognized by 1 Fab bound (V033-a I1) and 3 Fab bound (T6_P_H03) envelope complexes from V033-a variant cryo-EM structures. The

structures of V033a UCA I1 and T6_P_H03 are superimposed by gp120 alignment of protomer A. The conformation of the two N160 glycans recognized by a single V033a UCA I1 Fab (blue glycans) is compatible with recognition by multiple Fabs (tan glycans).

(**D**) V033a-UCA I1 and T6_P_H03 HCDR3s each recognize the C-strand with four identical antiparallel main chain hydrogen bonds.

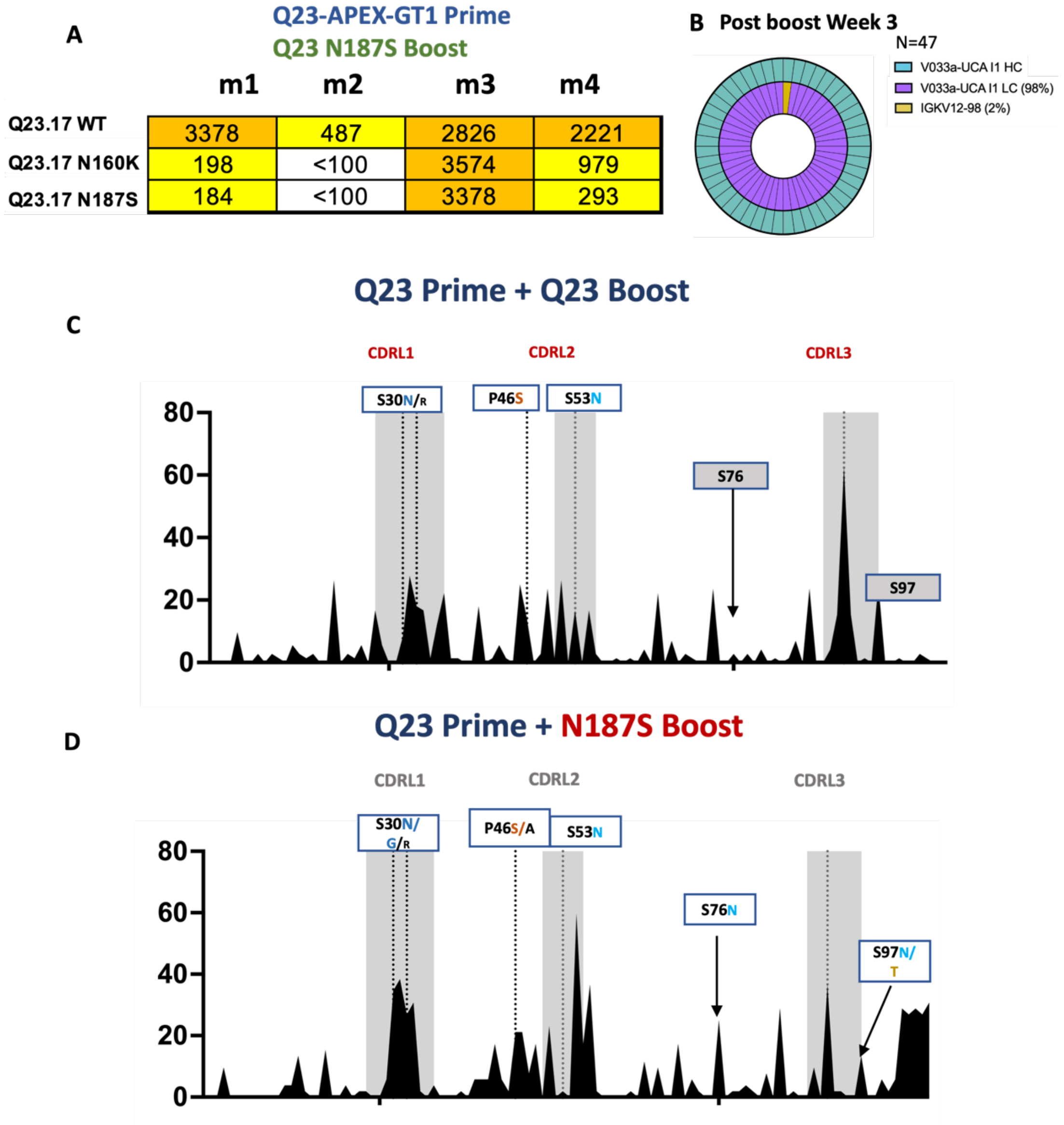


**Supplementary Figure 12. Heterologous boost leads to expanded serum neutralization and LC usage in homologous vs N187S boost, related to Figures 6 and 7.**

(**A**) Reverse ID50 of serum of animals receiving Q23-GT1 Prime and Q23-N187S boost against Q23.17, Q23.17 N160K and Q23.17 N187S escape variant.

(**B**) Light chain usage after boost.

(**C,D**) LC mutation frequencies after week 3 post immunization of V033_UCA I1 KI LC after (C) Q23-

APEX-GT1 boost and (D) Q23-APEX-GT1 N187S boost. Some of the selected mutations present in mature V033 lineage bnAbs are represented in red and intermediate mutations are represented in blue.

**A**

| Panel | Strain | V033 I1KI T3_QB_G12 | V033 I1KI T3_NB_G05 | V033 I1KI T3_NB_B07 | RMV033 a.01 |
|---|---|---|---|---|---|
| Global panel | 246F3 | 8.7 | 6.9 | 8.0 | 0.034 |
| | X1632 | >50 | >50 | >50 | >50 |
| | 25710 | 3.7 | 1.1 | 4.6 | 0.029 |
| | CNE55 | >50 | >50 | >50 | >50 |
| | X2278 | >50 | >50 | >50 | 0.73 |
| | BJOX0020000 | 0.75 | 0.87 | 0.69 | 0.040 |
| | CNE8 | | | | >50 |
| | CE0217 | >50 | 1.7 | 5.9 | 0.014 |
| | TRO.11 | >50 | >50 | >50 | >50 |
| | CE1176.A3 | >50 | >50 | >50 | >50 |
| | CH119.10 | 24 | 18 | >50 | 0.11 |
| | BG505.T332N | 0.31 | 0.62 | 0.68 | 0.020 |
| | BG505.T332N.N160K | >50 | >50 | >50 | 0.32 |
| | CAP256SU | >50 | >50 | >50 | >50 |
| | ZM233.6 | >50 | >50 | >50 | >50 |
| | WITO.33 | 0.40 | 1.5 | 4.2 | 0.006 |
| | T250-4 | 17 | 9.6 | >50 | 0.23 |
| | T250-4 N160K | >50 | >50 | >50 | >50 |
| | Q23.17 | 0.028 | 0.034 | 0.016 | 0.017 |
| | Q23.17 N187S | 0.33 | 0.16 | 1.1 | 0.12 |
| | Q23.K171R.N187S | >50 | 15 | >50 | 0.41 |
| | CH505 TF | >50 | >50 | >50 | >50 |
| | MT145K (SIVcpz) | 40 | 4.4 | 24 | 0.041 |
| | CAM13K (SIVcpz) | 0.62 | 1.6 | 2.2 | 0.045 |
| | C1080 | 0.016 | 0.024 | 0.011 | 0.006 |
| Hraber Panel | 9004SS_A3_4 | 1.9 | 1.4 | 4.4 | >50 |
| | Q842.D12 | 0.95 | 0.46 | 1.1 | 0.027 |
| | 249M_B10 | 7.1 | 3.2 | 2.7 | >50 |
| | 7060101641A7(REV-) | >50 | >50 | >50 | >50 |
| | HIV-001428-2.42 | 0.73 | 0.12 | 0.25 | 0.049 |
| | HIV-16055-2.3 | 0.68 | 0.33 | 0.55 | 0.080 |
| | HIV-25925-2.22 | 21 | 6.7 | >50 | 4.6 |
| | 3728.v2.c6 | >50 | >50 | >50 | >50 |
| | RPW-0510.2 | >50 | >50 | >50 | >50 |
| | H029.12 | >50 | >50 | >50 | >50 |
| | SPK-0525.13 | 0.24 | 0.58 | 1.8 | 0.11 |
| | Geometric Mean IC50: | 1.40 | 1.07 | 1.18 | 0.05 |
| | Median IC50: | 0.85 | 1.42 | 2.03 | 0.04 |
| | Percent Breadth: | 54.55 | 57.58 | 48.48 | 54.55 |

**B**

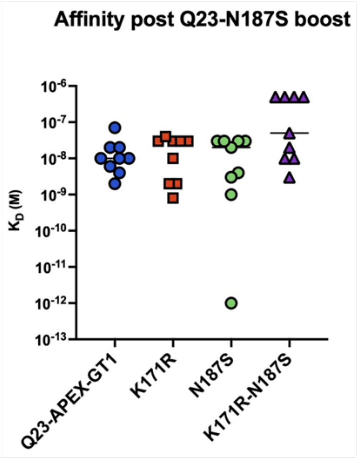


**Supplementary Figure 13. Neutralization of Q23 and N187S boost-derived antibodies, related to Figures 6, 7, and 8.**

(**A**) IC50 values of three antibodies against a 37-member panel of HIV-1 strains including 11 from the Tier-2 Global panel and 11 from the Hraber panel (*82*). Nomenclature: T = time in weeks post-boost; QB= Q23-APEX-GT1 Boost; NB= Q23-APEX-GT1 N187S Boost; final alphanumeric triplet = mAb identity

(**B**) Affinities ($K_D$ (M)) of boost-derived antibodies (10 µg/ml) from panel A against autologous Q23-APEX-GT1 Env and escape-variant Envs (500 nM).

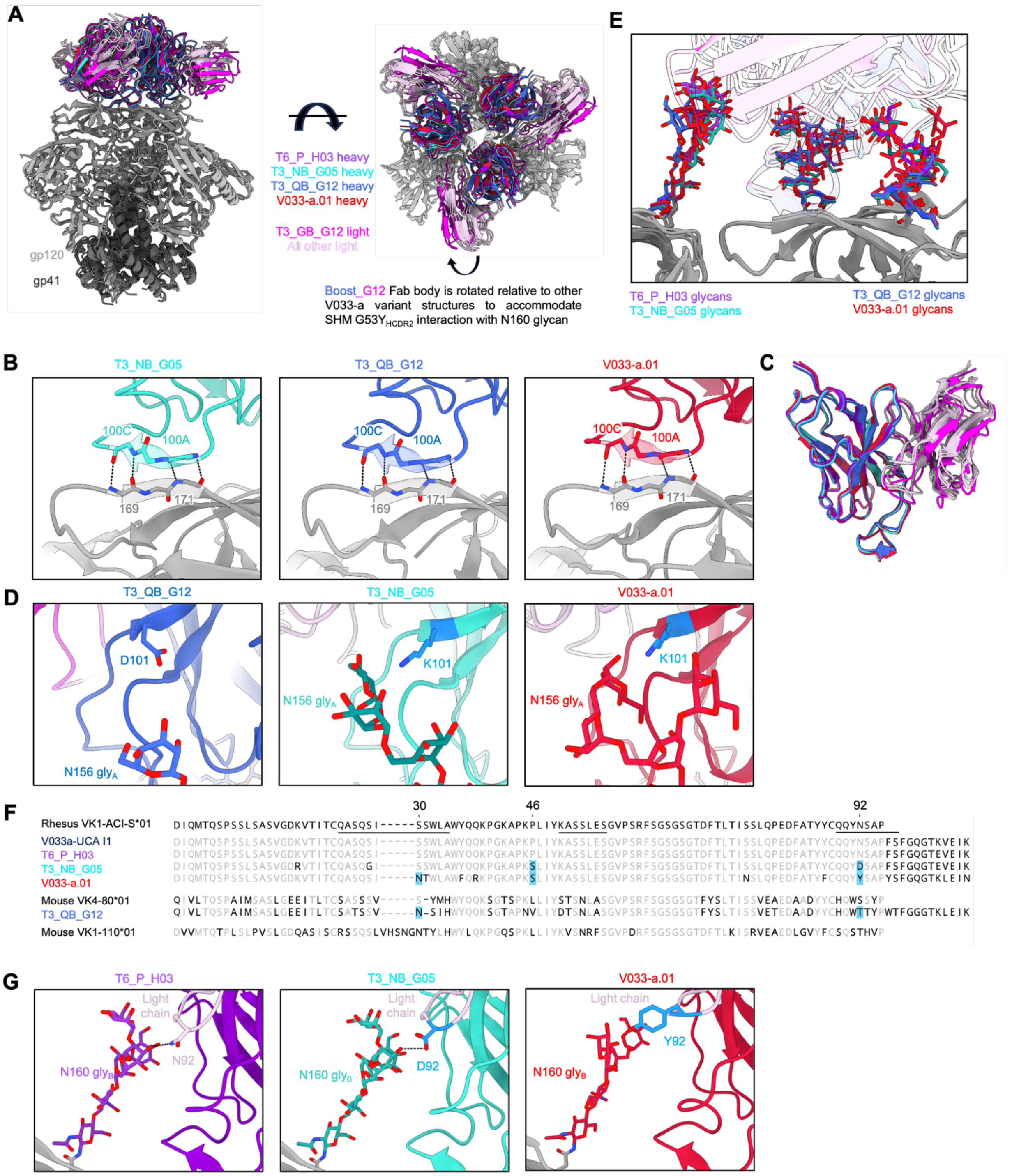


**Supplementary Figure 14. Select comparative structural features of V033-a lineage variants, related to Figures 5 and 8.**

(**A**) Orthogonal views for the gp120 alignment of T6_P_H03, T3_NB_G05, T3_QB_G12, and V033-a.01 cryo-EM structures in complex with Env trimers.

(**B**) Boost-derived antibodies each recognize the C-strand with four antiparallel mainchain hydrogen bonds identical to rhesus V033-a lineage members, including V033-a.01.

(**C**) Superimposition of T6_P_H03, T3_NB_G05, T3_QB_G12, and V033-a.01 Fabs from their respective complex structures. The use of a mouse-derived light chain does not alter the structure of antibody T3_QB_G12 and likely does not contribute to the unique angle of approach relative to all other V033-a lineage variants. Heavy and light chains are colored similarly to (A).

(**D**) The improbable HCDR3 K101 mutation in V033-a lineage variants T3_NB_G05 and V033-a.01 mediates recognition of N156 glycan from protomer A. In the T3_QB_G12 structure bearing the germline residue D101, the visible reconstruction density reveals N156 glycan on protomer A to not extend as far and does not interact with this residue.

(**E**) Comparing the conformation of apical glycans recognized by T6_P_H03, T3_NB_G05, T3_QB_G12, and V033-a.01. A single Fab recognizing the C-strand of protomer A binds to the N160 and N156 glycans from protomer A and N160 glycan from protomer B. All apical glycans adopt highly similar conformations when recognized by V033-a lineage variants. Glycans are colored according to the corresponding antibody heavy chain from panel A. Fab structures are made transparent to better show glycans. Only one Fab per structure is shown for clarity.

(**F**) Light chain amino acid sequences of T6_P_H03, T3_NB_G05, T3_QB_G12, V033-a.01, and V033-a I1 are aligned to germline rhesus VK1-ACI-S*01 gene. Antibody T3_QB_G12 is derived from a mouse light chain, and its germline gene VK4-80*01 is included in the alignment as well to visualize T3_QB_G12 light chain SHM. Many V033-a variant mouse antibodies were also

derived from lights chains expressing VK1-110*01, and this sequence is included in the alignment as well. Residues matching the reference are depicted in light gray and nonmatching residues are depicted in black. The LCDRs are overscored in the V033-a I1 sequence. Positions of somatic hypermutation shared by one or two murine antibodies with V033-a.01 are highlighted in light blue.

(**G**) V033-a lineage variant antibodies bearing rhesus light chains recognize N160 glycan from protomer B using LCDR3 residue 92. The D92 SHM in T3_NB_G05 does not significantly alter the N160 glycan-recognizing paratope over germline N92 present in T6_P_H03; however, the V033-a.01 LCDR3 adopts a slightly different conformation and uses aromatic stacking to recognize N160 glycan via Y92 SHM.

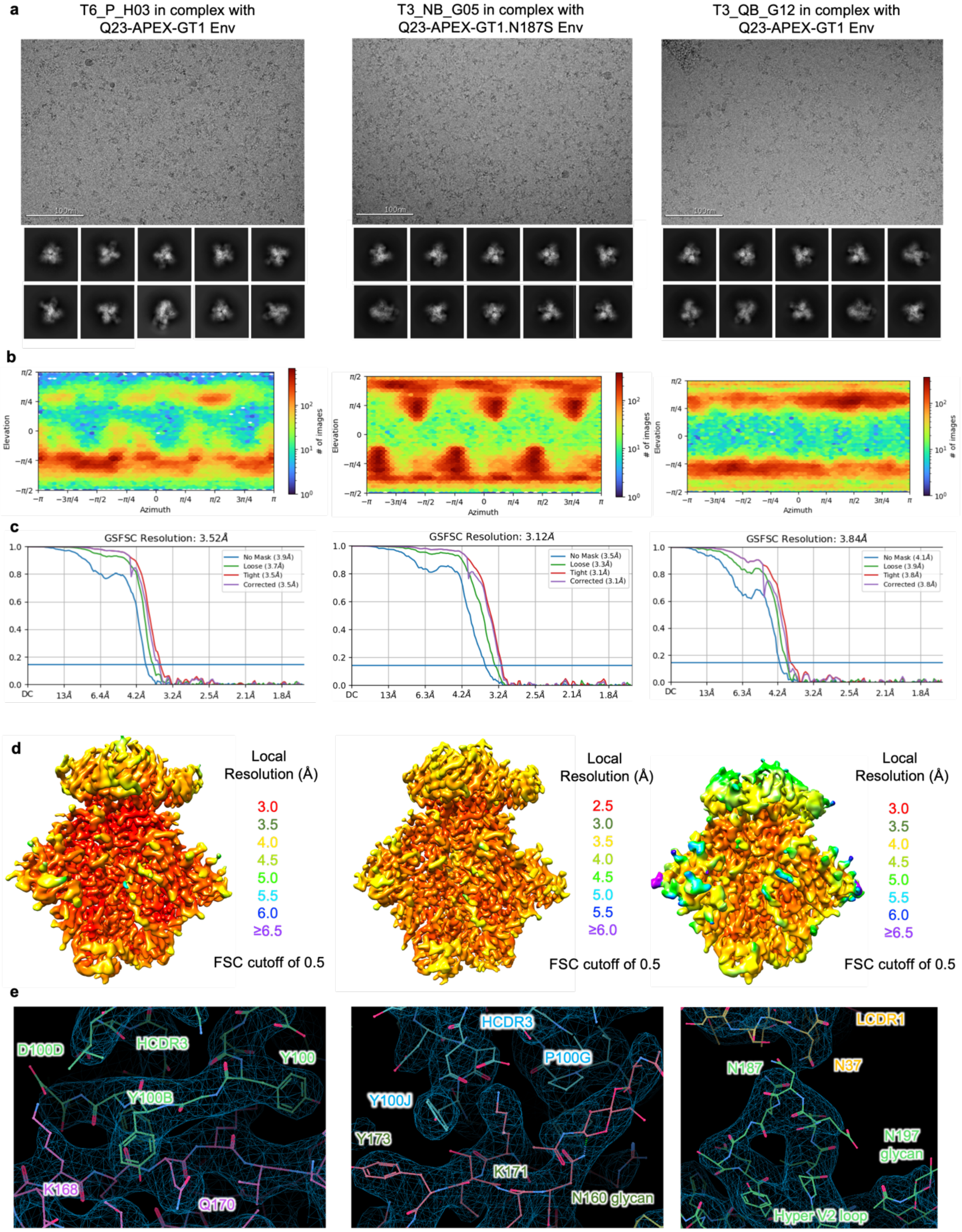


**Supplementary Figure 15. Single-particle cryo-EM validation for murine V033-a antibodies in**

**complex with HIV envelope, related to Figure 5.**

(**A**) Representative raw micrograph with representative 2D class averages of picked particles shown below.

(**B**) Orientations of all particles used in the final refinement are shown as a heatmap.

(**C**) Gold-standard Fourier shell correlation (FSC) curves with auto-tightening using a non-uniform refinement with C3 symmetry.

(**D**) Local resolution estimation of the full map is shown as generated through cryoSPARC using an FSC cutoff of 0.5.

(**E**) Example of high-resolution cryo-EM 3D reconstruction density to highlight regions of the Fab:trimer interface.

**Supplementary Table 1. Post-boost mutation frequencies.** Frequencies of selected mutations and their probability predicted by ARMADiLLO in V033a-UCA I1 at week 3 after Q23-APEX-GT1 boost or Q23-N187S boost after priming immunization with Q23-APEX-GT1.

| Position | Mutation | Mutation probablity by ARMADiLLO | Frequency (%) after Q23-GT1 boost | Frequency (%) after Q23-N187S boost |
|---|---|---|---|---|
| HCDR1 | S31N | 1-2% | 86.67 | |
| HCDR1 | S31D | 0.1-1% | 13.33 | |
| HCDR2 | S56Y | 0.1-1% | 28.48 | |
| HCDR2 | T57R | 1-2% | 44.24 | |
| HCDR3 | E97D | 2-10% | 60.61 | |
| HCDR3 | $Y100_{C}F$ | 2-10% | 61.21 | |
| HCDR3 | $Y100_{D}D$ | 2-10% | 22.42 | |
| HCDR3 | D100K | <0.01% | 0.60 | |

## Supplementary Table 2. Cryo-EM Statistics.

| | T6_P_H03 in complex with Q23-APEX-GT1 Env | T3_NB_G05 in complex with Q23-APEX-GT1.N187S Env | T3_QB_G12 in complex with Q23-APEX-GT1 Env |
|---|---|---|---|
| **PDB** | **9OOM** | **9OOG** | **9OOK** |
| **EMDB** | **70666** | **70663** | **70664** |
| **Data collection & processing** | | | |
| Microscope | FEI Titan Krios | FEI Titan Krios | FEI Titan Krios |
| Camera | Gatan K3 | Gatan K3 | Gatan K3 |
| Magnification | 105,000x | 105,000x | 105,000x |
| Voltage (kV) | 300 | 300 | 300 |
| Electron dose ($e^-/Å^2$) | 58 | 58 | 58 |
| Defocus range (μm) | 0.8 - 2.0 | 0.8 - 2.0 | 1.0 - 2.5 |
| Pixel size (Å) | 0.83 | 0.83 | 0.83 |
| Micrographs collected | 4,956 | 8,638 | 8,970 |
| Software | cryoSPARC v4.1 | cryoSPARC v4.1 | cryoSPARC v4.1 |
| Micrographs used | 4,183 | 7,862 | 7,991 |
| Refined particles | 140,657 | 211,774 | 140,366 |
| Symmetry imposed | C3 | C3 | C3 |
| Map Resolution (Å) | 3.52 | 3.12 | 3.84 |
| FSC threshold | 0.143 | 0.143 | 0.143 |
| **Refinement & validation** | | | |
| Initial model used | 9BNP | 9BNP | 9BNP |
| Software | Phenix 1.21 | Phenix 1.21 | Phenix 1.21 |
| Number of residues | | | |
| Protein | 2,433 | 2,433 | 2,430 |
| Ligand | 145 | 141 | 132 |
| Map CC | 0.83 | 0.85 | 0.80 |
| R.m.s. deviations | | | |
| Bond lengths (Å) | 0.006 | 0.005 | 0.004 |
| Bond angles (˚) | 1.01 | 0.897 | 0.931 |
| EMRinger score | 3.01 | 2.72 | 1.78 |
| MolProbity score | 1.56 | 1.27 | 1.43 |
| Clashscore | 4.29 | 2.08 | 3.14 |
| Rotamer outliers (%) | 0 | 0 | 0 |
| Ramachandran plot | | | |
| Favored (%) | 95.0 | 95.9 | 95.3 |
| Allowed (%) | 5.0 | 4.1 | 4.7 |
| Outliers (%) | 0 | 0 | 0.0 |

**Supplementary Table 3. Reagents used.**

| Reagent | Source | Identifier |
|---|---|---|
| Rat monoclonal anti-mouse-CD16/32 purified | BD Biosciences | Clone 2.4G2<br># 553142; |
| Rat monoclonal anti-mouse CD4 APC-eF780 | Thermo Fisher Scientific | Clone RM4-5<br># 47-0042-80 |
| Rat monoclonal anti-mouse CD8 APC-eF780 | Thermo Fisher Scientific | Clone 53–6.7<br># 47-0081-80 |
| Rat monoclonal anti-mouse F4/80 APC-eF780 | Thermo Fisher Scientific | Clone BM8<br># 47-4801-80 |
| Rat monoclonal anti-mouse Ly-6G APC-eF780 | Thermo Fisher Scientific | Clone RB6-8C5<br># 47-5931-80 |
| Rat monoclonal anti-mouse NK1.1 APC-eF780 | Thermo Fisher Scientific | Clone PK136<br># 47-5941-82 |
| Rat anti-mouse B220 BUV395 or Alexa Fluor 594 or BUV615 | BD Biosciences<br>BioLegend | Clone: RA3-6B2<br># 563793 & #103254 #569724 |
| Hamster anti-mouse CD95 PE-Cy7 | BD Biosciences | Clone Jo2<br># 557653 |
| Rat anti-mouse CD38 BUV563 or BB700 | BD Biosciences | Clone 90<br>#741271 & #742132 |
| Mouse anti-mouse CD45.2 PE or BB700 | BioLegend<br>BD Biosciences | Clone 104<br># 109808 |
| Mouse anti-mouse CD45.1 BV605 or APC-R700 | BD Biosciences | Clone A20<br># 747743 & #565814 |
| Rat anti-mouse CD138 BV650 | BD Biosciences | Clone 281-2<br>#564038 |
| Rat anti-mouse IgD Alexa Fluor 594 | BioLegend | Clone: 11-26 c.2a<br># 405740 |
| Rat anti-mouse IgM BV750 | BD Biosciences | Clone: Il/41<br>#747333 |
| Rat anti-mouse IgG1 BUV805 | BD Biosciences | Clone Rat X56<br># 748402 |
| Rat anti-mouse IgG2a/IgG2b BUV805 | BD Biosciences | Clone Rat 2-40<br>#749169 |
| Rat anti-mouse IgG3 BUV805 | BD Biosciences | Clone R40-82<br># 749007 |
| Rat anti-mouse CD273 BV421 | BD Biosciences | Clone Rat MIH37<br>#567374 |
| Rat monoclonal anti-mouse GL7 Alexa Fluor 488 | Biolegend | Clone GL7<br>#144612 |
| Rat anti-mouse Ig,k light chain BUV395 | BD Biosciences | Clone 187.1<br>#742839 |
| Rat anti-mouse Ig,$\lambda$1, $\lambda$2, $\lambda$3 light chain BV711 | BD Biosciences | Clone R26-46<br>#744527 |

| | | |
|---|---|---|
| Brilliant Stain Buffer | BD Biosciences | 563794 |
| Hashtag-C01 | Biolegend | Clone M1/42; 30-F11 # 155861 |
| Hashtag-C02 | Biolegend | Clone M1/42; 30-F11 # 155863 |
| Hashtag-C03 | Biolegend | Clone M1/42; 30-F11 # 155865 |
| Hashtag-C04 | Biolegend | Clone M1/42; 30-F11 # 155867 |
| Hashtag-C05 | Biolegend | Clone M1/42; 30-F11 # 155869 |
| Hashtag-C06 | Biolegend | Clone M1/42; 30-F11 # 155871 |
| Hashtag-C07 | Biolegend | Clone M1/42; 30-F11 # 155873 |
| Hashtag-C08 | Biolegend | Clone M1/42; 30-F11 # 155875 |
| Hashtag-C09 | Biolegend | Clone M1/42; 30-F11 # 155877 |
| Hashtag-C10 | Biolegend | Clone M1/42; 30-F11 # 155879 |
| | | |
| Streptavidin-BV510 | BD Biosciences | #563261 |
| Streptavidin-AF647 | BioLegend | #405237 |
| Live/Dead Blue Fixable stain | Thermo Fisher | # L23105 |
| Sytox Green | Thermo Fisher Scientific | # S7020 |
| E-Gel 96 2% with SYVR Safe | Fisher Scientific | G720802 |
| 40 K polyethylenimine (PEI) MAX | Kyfora | # 24765-1 |
| FectoPRO | Polyplus | # 116-001 |
| Lipofectamine 2000 | Fisher Scientific | 11668500 |
| Valproic acid sodium salt | Sigma | #P4543-100G |
| D-(+)-Glucose Solution | Gibco | #A2494001 |
| L-glutamine | Corning | # 25-005-CI |
| Ig Elution Buffer | Thermo Fisher Scientific | PI21009 |
| Penicillin-streptomycin | Corning | # 30-002-CI |
| DEAE-dextran | Sigma-Aldrich | # 93556-1G |
| Phosphatase substrate | Sigma-Aldrich | #S0942-200TAB |
| Expi293 Expression Medium | Thermo Fisher Scientific | # A14351-01 |
| Freestyle Media | Thermo Fisher Scientific | #12338-018 |
| OptiMEM | Thermo Fisher Scientific | #31985070 |
| DMEM | Corning | # 10-017-CV |
| EDTA | Invitrogen | #15575-038 |
| RPMI | Corning | MT15040CV |
| FBS | Thermo Fisher Scientific | MT35016CV |
| Trypan Blue | Sigma | T8154 |
| Dnase 1 | Qiagen | #79254 |
| Bovine Serum Albumin | Sigma-Aldrich | A9418-500G |
| Tween20 | Sigma-Aldrich | #1003620819 |
| SuperScript™ IV Reverse Transcriptase | ThermoFisher Scientific | #1750150 |
| ExoSAP-IT™ PCR Product Cleanup Reagent | ThermoFisher Scientific | #78205 |
| HotStarTaq Plus DNA Polymerase | ThermoFisher Scientific | #203603 |
| Q5 Hot Start High-Fidelity 2X Master Mix | New England Biolabs | #M0494S |
| AgeI-HF | New England Biolabs | #R3552S |
| NheI-HF | New England Biolabs | #R3131S |
| BsiWI-HF | New England Biolabs | #R3553S |
| SalI-HF | New England Biolabs | #R3138S |

| | | |
|---|---|---|
| SPRIselect | Beckman Coulter Genomics | #B23318 |
| Magnesium Chloride Hexahydrate | Fisher bioreagents | #BP214-500 |
| Sodium carbonate | Sigma-Aldrich | #S7795-500G |
| Sodium azide | Sigma-Aldrich | #S2002-100G |
| Acetonitrile, 80%, 20% Water with 0.1% Formic Acid, Optima LC/MS | Fisher Scientific | Cat# 15431423 |
| Water with 0.1% Formic Acid (v/v), Optima™ LC/MS Grade | Fisher Scientific | Cat# LS118-212 |
| Acetonitrile | Fisher Scientific | Cat# 10489553 |
| Trifluoroacetic acid | Fisher Scientific | Cat# 10155347 |
| Dithiothreitol | Sigma-Aldrich | Cat# 43819 |
| Iodoacetamide | Sigma-Aldrich | Cat# I1149 |
| Mass spectrometry grade trypsin | Promega | Cat# V5280 |
| Sequencing grade chymotrypsin | Promega | Cat# V1061 |
| α-Lytic protease | New England Biolabs | Cat# P8113S |
| Pierce Fab Preparation Kit | Fisher Scientific | 44985 |
| cryovials | Globe Scientific | 3010 |
| Sterile vacutainers | DB vacutainer | 364606 |
| cryovial | Sarstedt | 72.694.396 |
| ViaStain AOPI solution | Revvity | CS2-0106-25ml |
| Cell strainer | Falcon | 352360 |
| 5 ml round bottom tube | Corning | 352058 |
| ExpiFectamine 293 transfection reagents | Gibco | Cat# A14524 |
| Protein A/Protein G GraviTrap kit | Cytiva | Cat# 28-9852-56 |
| Galanthus Nivalis Lectin (GNL) agrose bound | Vector Laboratorie | Cat# AL-1243-S |

| **Software and Algorithms** | | |
|---|---|---|
| IMGT/V-Quest | International ImMunoGeneTics Information System; Marie-Paule Lefranc (marie-paule.lefranc@igh.cnrs.fr), University of Montpellier, France | www.imgt.org; RRID: SCR_012780 |
| AbStar | Bryan Briney (briney@scripps.edu), The Scripps Research Institute | https://github.com/briney/abstar |
| Cellranger | 10X Genomics | https://support.10xgenomics.com/single-cell-gene-expression/software/downloads/latest |
| Prism 8 | GraphPad | https://www.graphpad.com/scientific-software/prism/ |
| ForteBio Data Analysis software | Sartorius | https://www.sartorius.com/en |
| PyMOL V2.4.2 | PyMOL by Schrödinger | https://pymol.org |
| UCSF Chimera | Pettersen et al., 2004 | http://plato.cgl.ucsf.edu/chimera/; RRID: SCR_004097 |
| FlowJo v.10 | BD Life Sciences | https://www.flowjo.com/solutions/flowjo |
| Cryosparc | V4.1 | https://cryosparc.com |
| Geneious Biologics | Geneious | https://www.geneious.com/features/biologics |
| Geneious Prime | Geneious | https://www.geneious.com/features/prime |

| | | |
|---|---|---|
| Illustrator | Adobe | https://www.adobe.com/products/illustrator.html |
